# Supplementary material for: Burden of chronic obstructive pulmonary disease in adults aged 70 years and older, 1990–2021: Findings from the Global Burden of Disease Study 2021
Source: PLoS One. 2025 Jan 8;20(1):e0316135. doi: 10.1371/journal.pone.0316135 (PMC11709235; doi:10.1371/journal.pone.0316135)
Supplement: S1 File — (DOCX) [file pone.0316135.s001.docx]

**Supplementary material**

**Burden of** **chronic obstructive pulmonary disease in adults aged 70 years and older, 1990-2021: findings from the Global Burden of Disease Study 2021**

**S1 Fig. The prevalence rates of COPD among adults aged ≥70 across 21 regions in 1990 and 2021.**


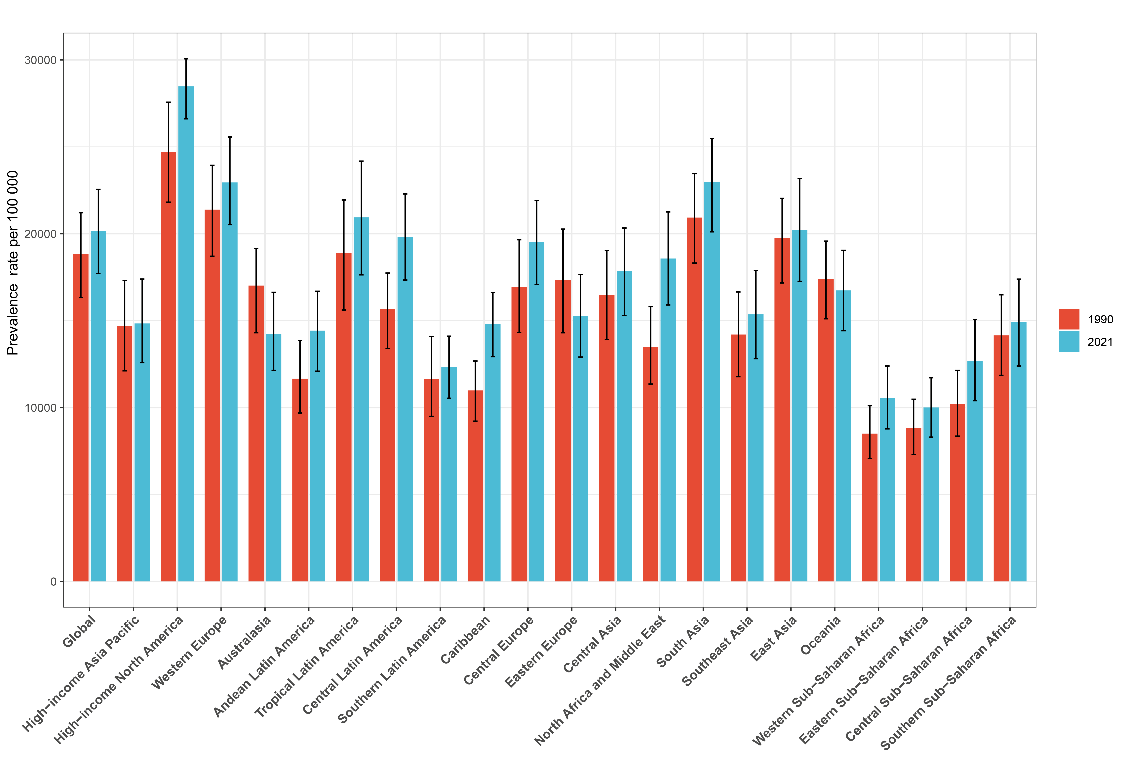


**S2 Fig. The incidence rates of COPD among adults aged ≥70 across 21 regions in 1990 and 2021.**


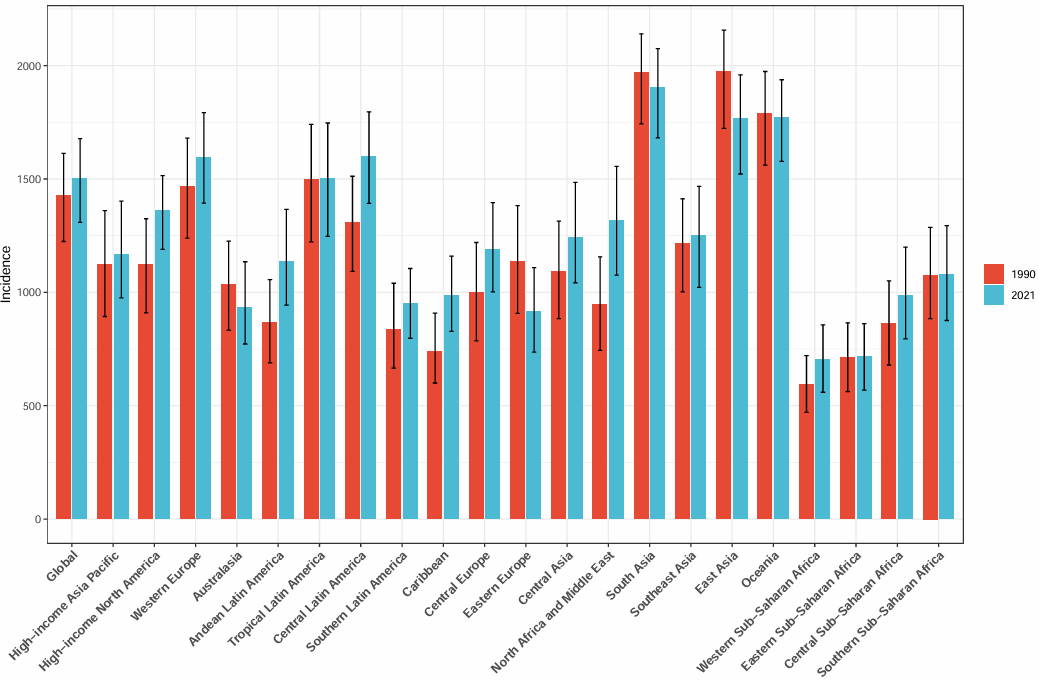


**S3 Fig. The prevalence rates of COPD among adults aged ≥70 across 21 regions grouped by sex.**
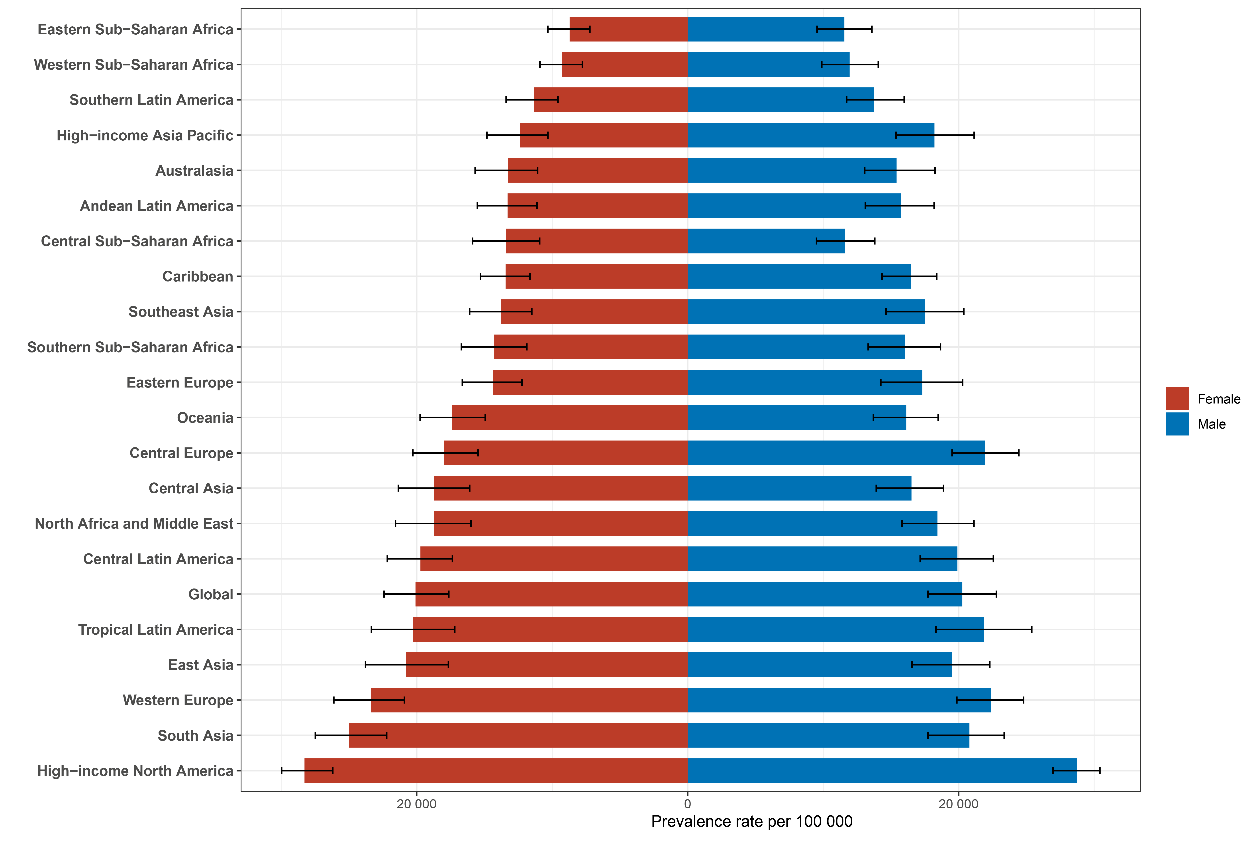


**S4 Fig. The incidence rates of COPD among adults aged ≥70 across 21 regions grouped by sex.**


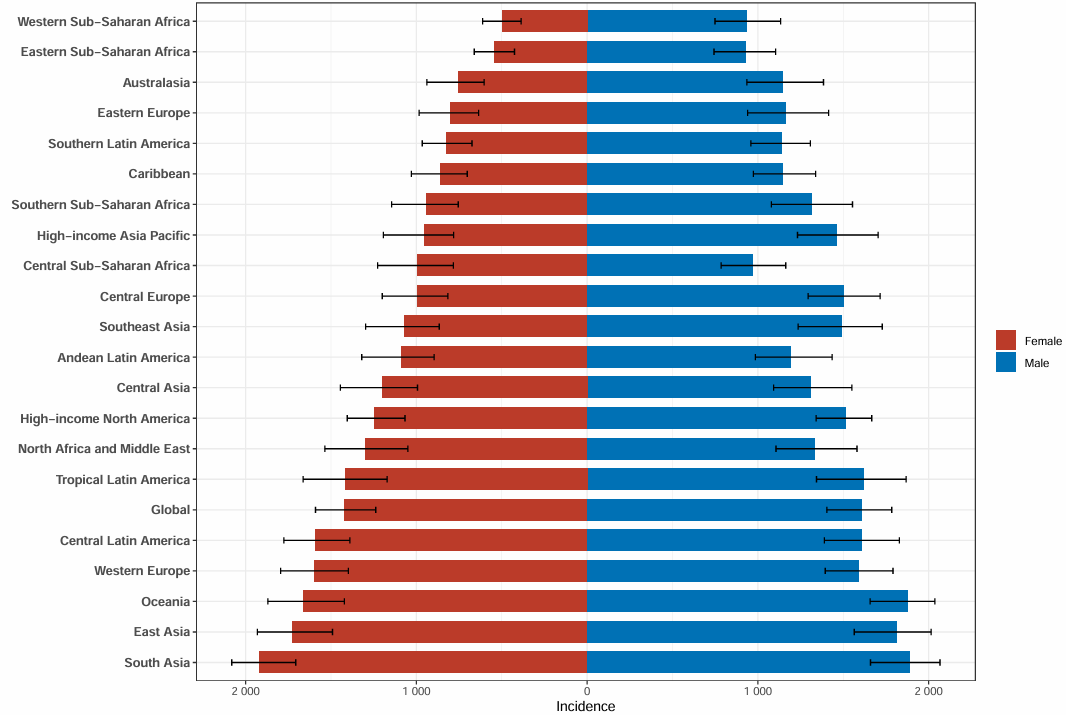


**S5 Fig. The association between SDI and COPD incidence rates among adults aged ≥70 years across 21 regions from 1990 to 2021.**


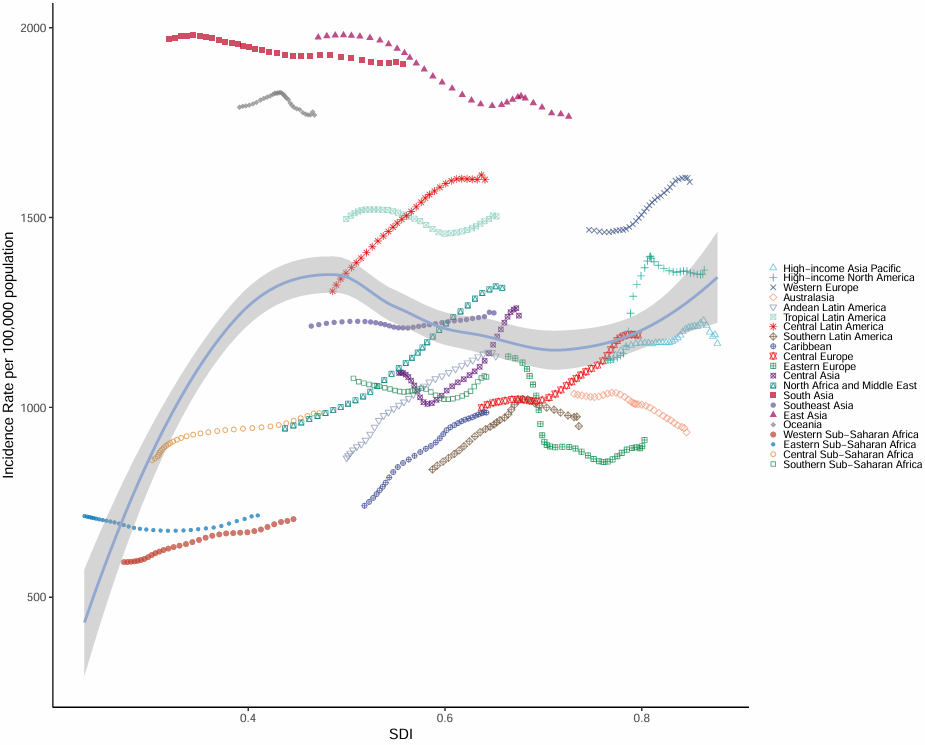


**S6 Fig. The death rates of COPD among adults aged ≥70 across 21 regions in 1990 and 2021.**


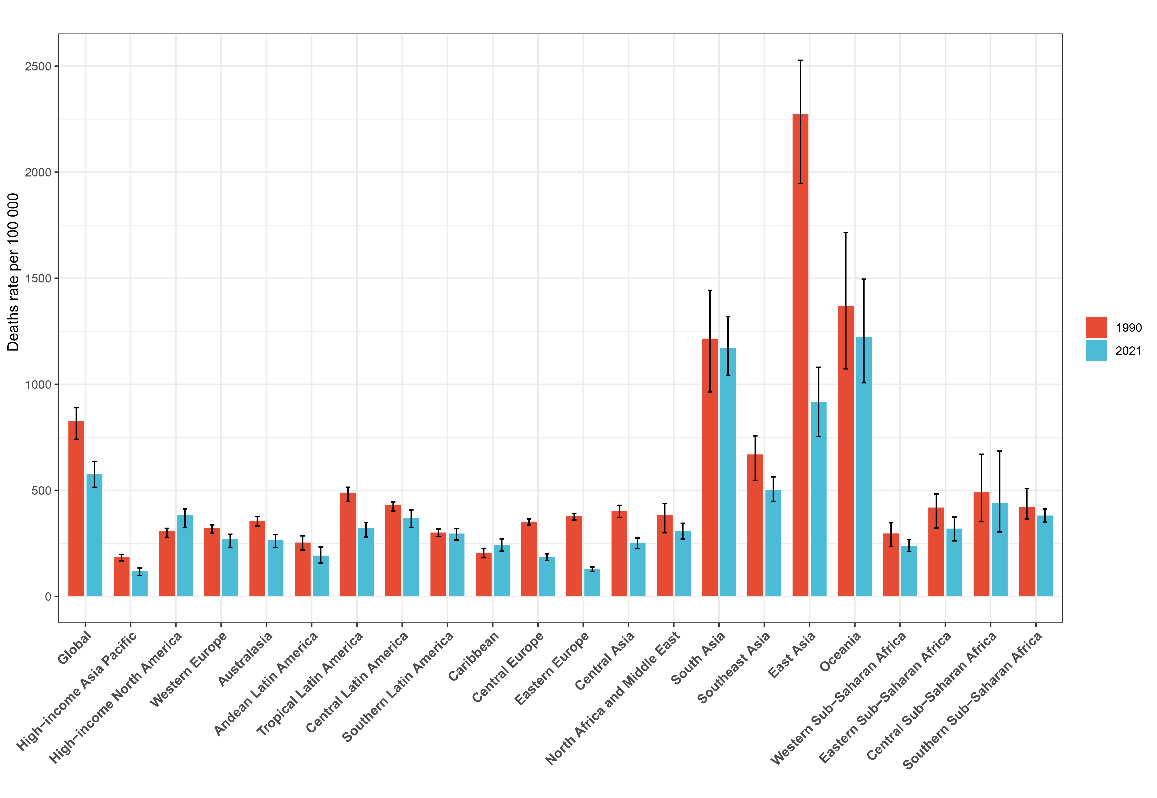


**S7 Fig. The death rates of COPD among adults aged ≥70 across 21 regions grouped by sex.**


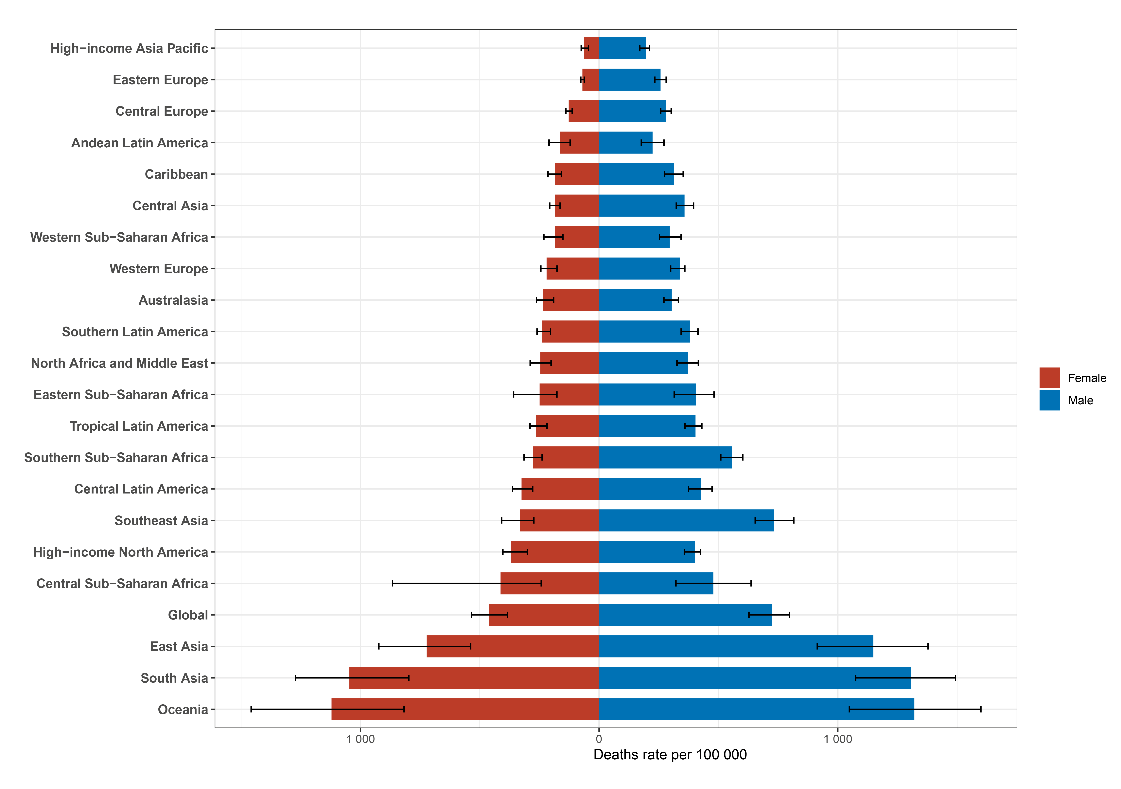


**S8 Fig. The association between SDI and COPD death rates among adults aged ≥70 years across 21 regions from 1990 to 2021.**


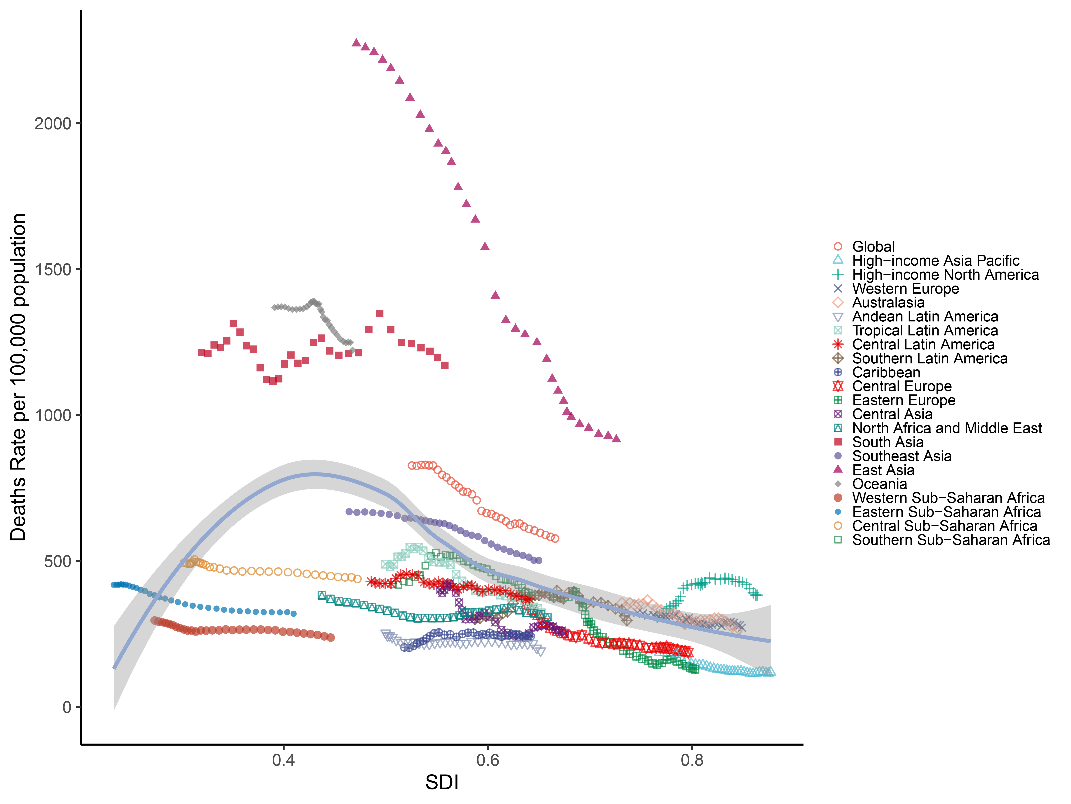


**S9 Fig. The DALYs rates of COPD among adults aged ≥70 across 21 regions in 1990 and 2021.**
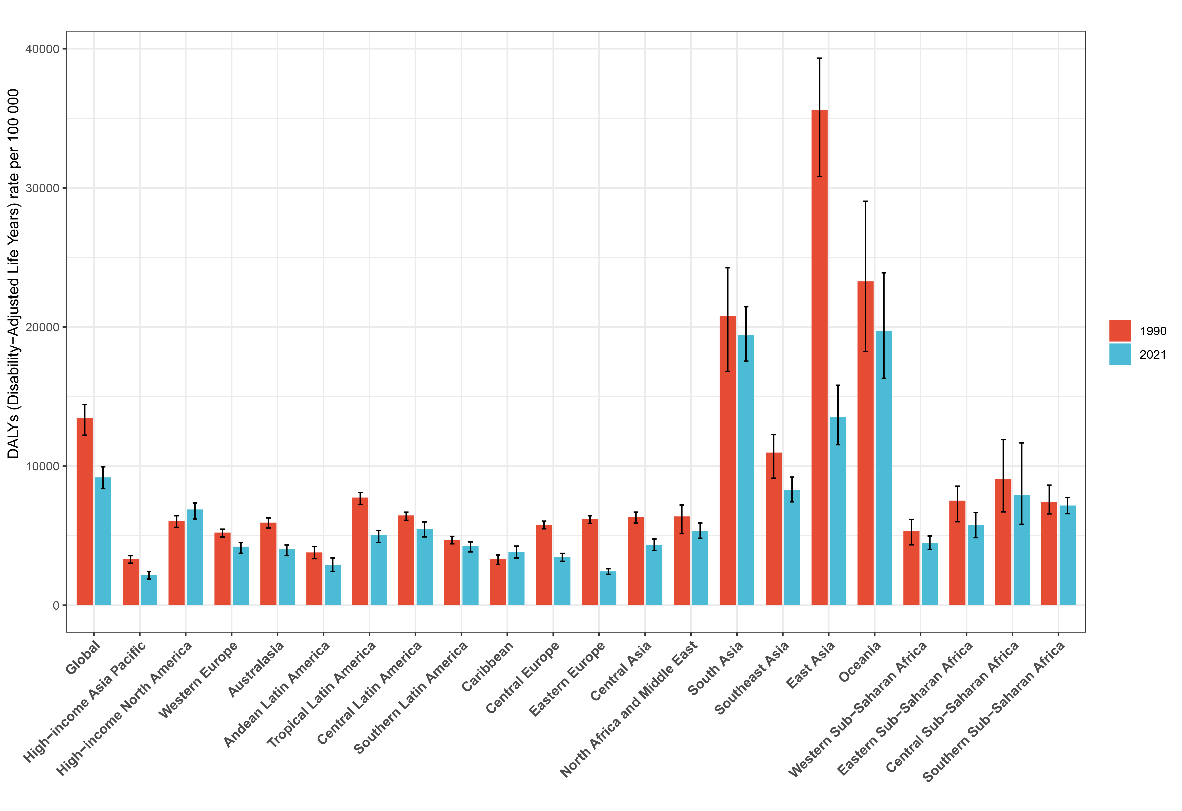


**S10 Fig. The DALYs rates of COPD among adults aged ≥70 across 21 regions grouped by sex.**


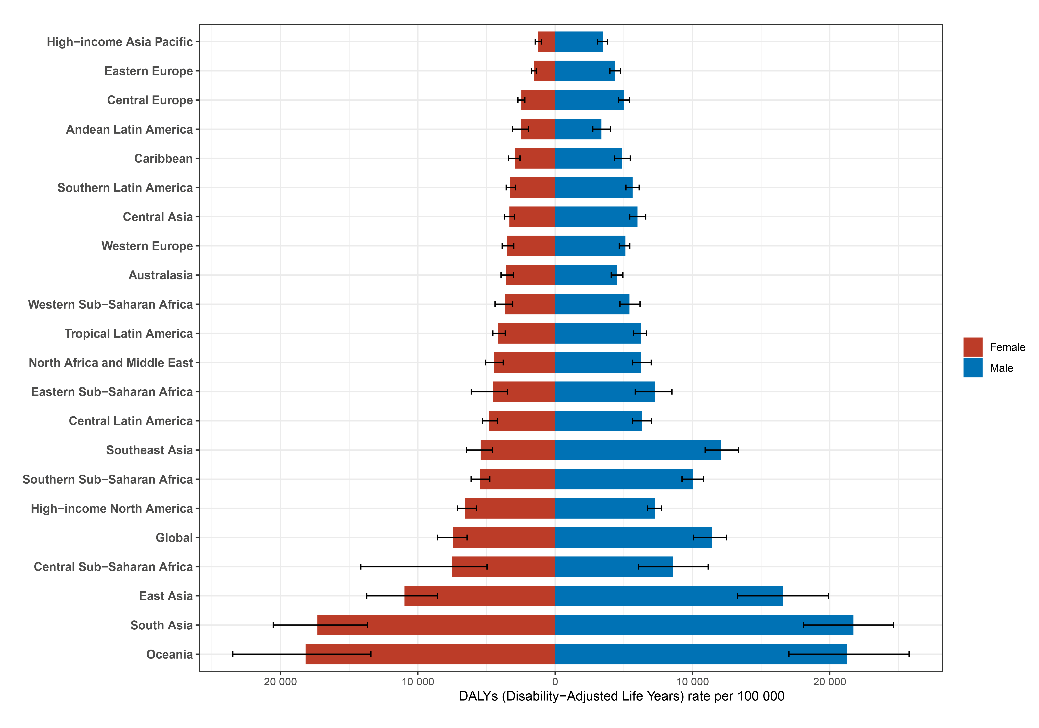


**S11 Fig. The association between SDI and the DALYs rates of COPD among adults aged ≥70 years across 21 regions from 1990 to 2021.**


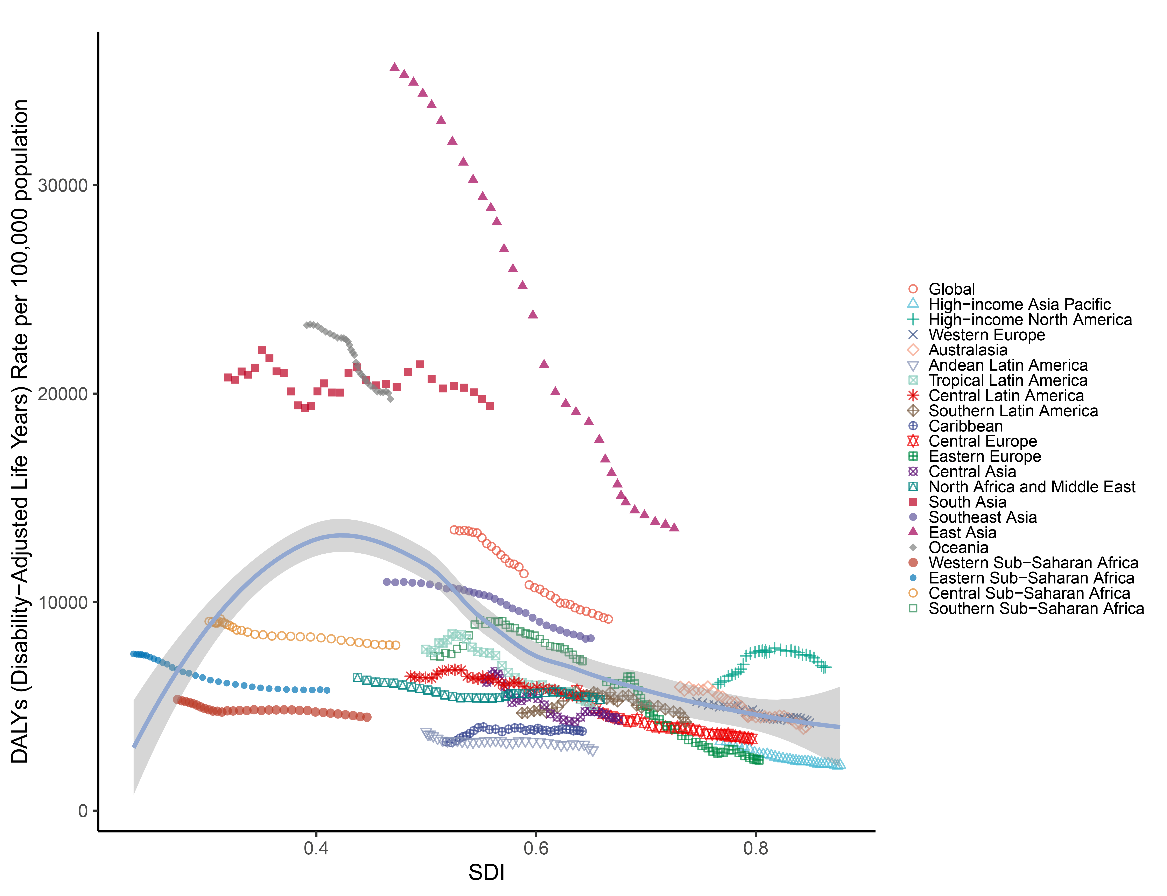


**S12 Fig. The association between SDI and the prevalence rates of COPD among adults aged ≥70 years across 204 countries from 1990 to 2021.**


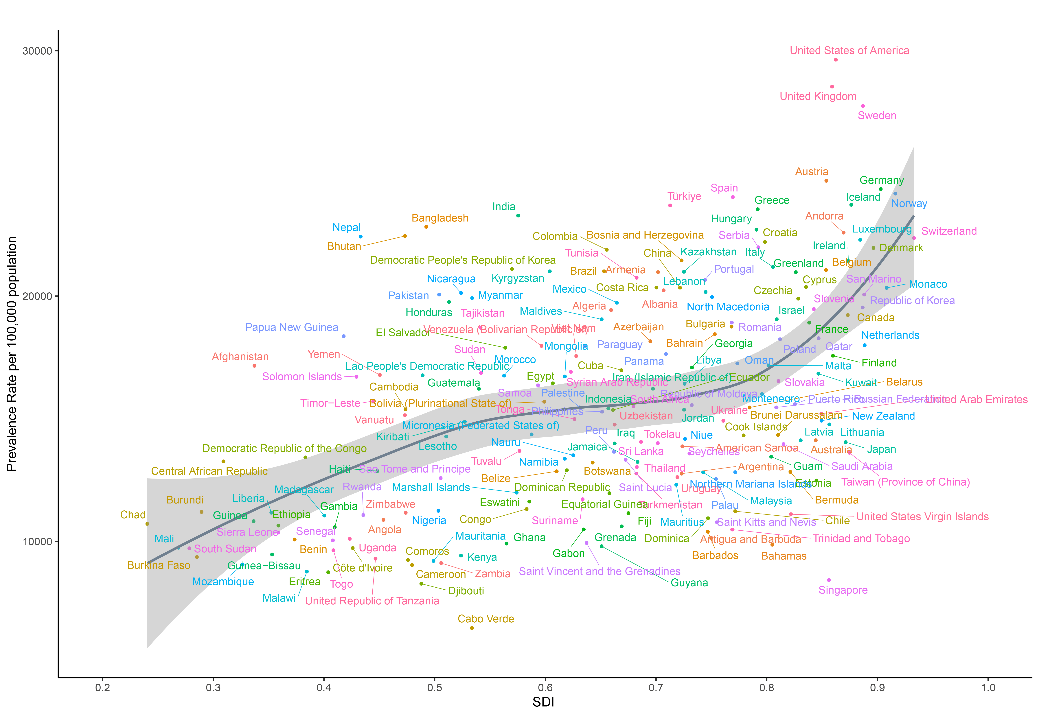


**S13 Fig. The association between SDI and the incidence rates of COPD among adults aged ≥70 years across 204 countries from 1990 to 2021.**


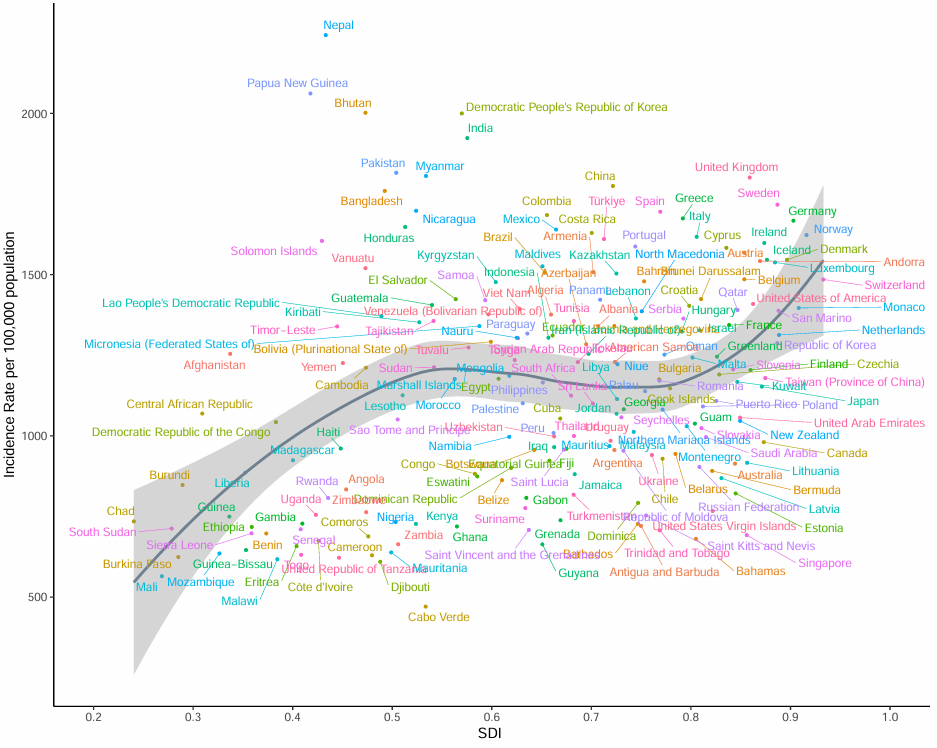


**S14 Fig. The association between SDI and the death rates of COPD among adults aged ≥70 years across 204 countries from 1990 to 2021.**


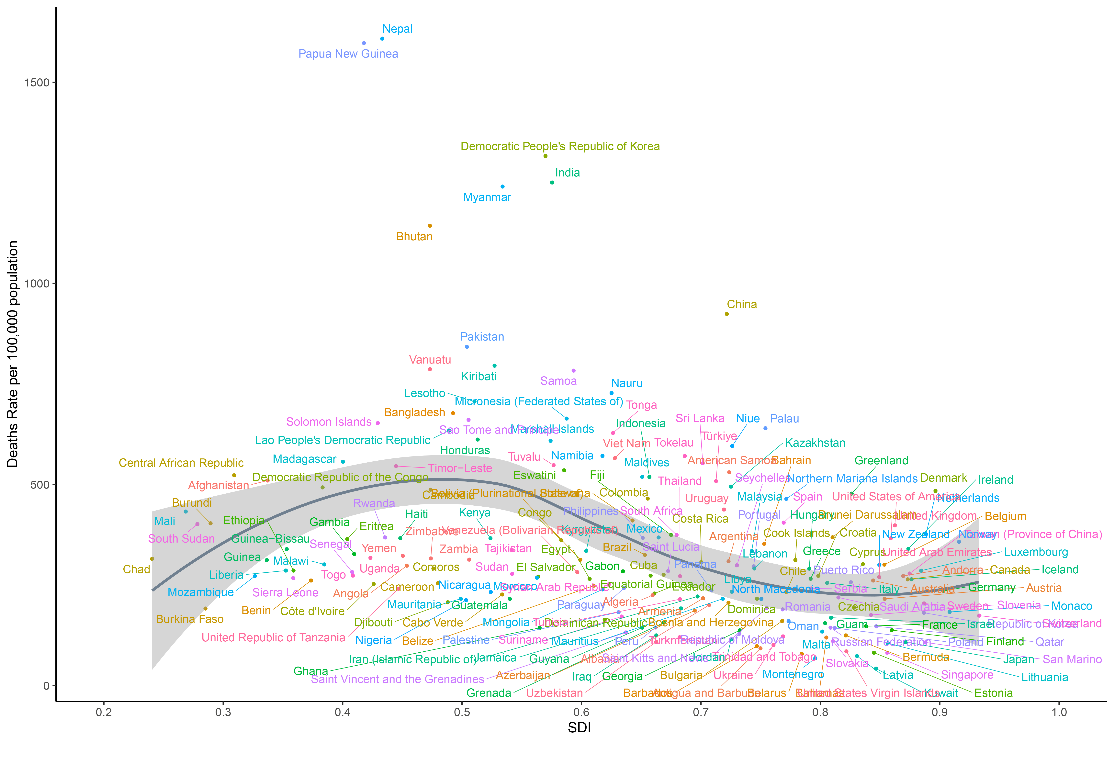


**S15 Fig. The association between SDI and the DALYs rates of COPD among adults aged ≥70 years across 204 countries from 1990 to 2021.**


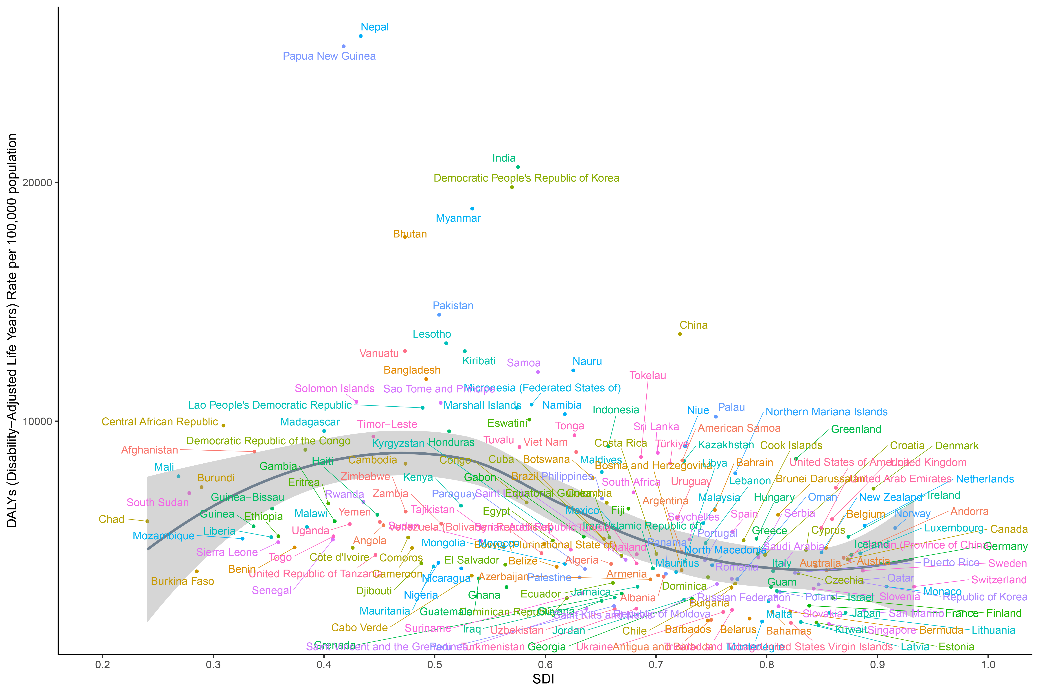


**S16 Fig. Death rates of COPD attributable to each risk factor among adults aged ≥70 years in 2021.**


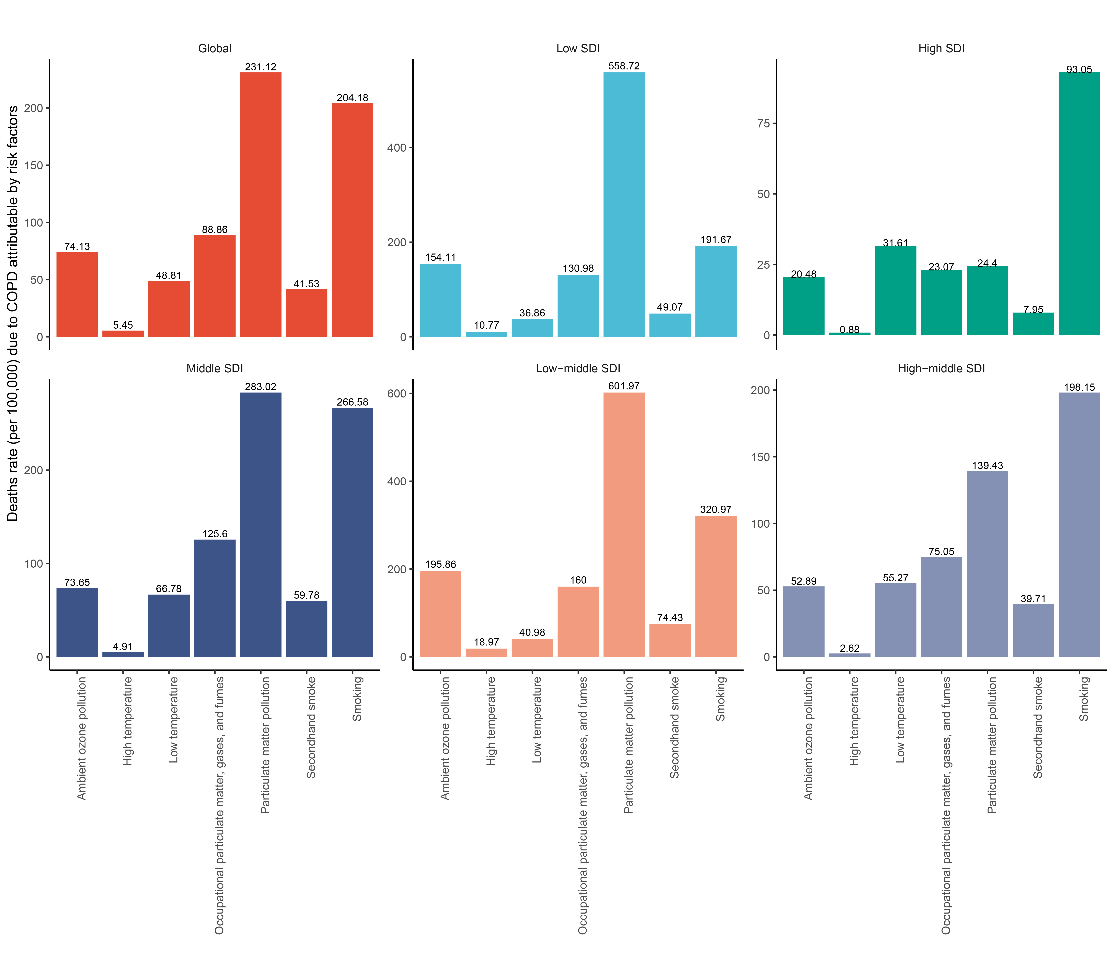


**S1 Table. Incidence and EAPC of chronic obstructive pulmonary disease among adults aged ≥70 years at the global and regional levels, from 1990 to 2021**

|  | **Incidence (95% UI)** | | | |  |
| --- | --- | --- | --- | --- | --- |
|  | **No in 1990, thousands** | **Rate in 1990 (per 100,000)** | **No in 2021, thousands** | **Rate in 2021 (per 100,000)** | **EAPCs (95% CI)** |
| **Global** | 2886.7 (2473-3258.4) | 1429 (1224.2-1613.0) | 7429 (6471.1-8295) | 1502.7 (1309.0-1677.9) | 0.17 (0.16-0.19) |
| **SDI** |  |  |  |  |  |
| High SDI | 867 (717.6-1005.1) | 1255.5 (1039.2-1455.5) | 1946.7 (1698.7-2199.4) | 1356.8 (1184.0-1532.9) | 0.32 (0.27-0.37) |
| High-middle SDI | 746.9 (639.3-848) | 1451.4 (1242.3-1647.8) | 1717.5 (1484-1941.9) | 1462.9 (1264.0-1654.1) | 0.01 (-0.05-0.07) |
| Middle SDI | 729.7 (628-808.4) | 1596.8 (1374.3-1769.2) | 2286.2 (1976.2-2550.1) | 1621.5 (1401.7-1808.7) | 0.02 (-0.02-0.05) |
| Low-middle SDI | 428.2 (373.8-470.5) | 1633 (1425.4-1794.2) | 1186.1 (1051.2-1295.8) | 1692.2 (1499.7-1848.7) | 0.11 (0.11-0.12) |
| Low SDI | 112.4 (97.1-126.1) | 1204.2 (1040.3-1351.3) | 286.8 (248.2-318.3) | 1307.6 (1131.4-1451.4) | 0.25 (0.23-0.26) |
| **Regions** |  |  |  |  |  |
| Andean Latin America | 8.8 (7-10.8) | 866.7 (688.7-1055.7) | 37.2 (31-44.8) | 1135 (943.7-1365.8) | 0.98 (0.92-1.03) |
| Australasia | 15.1 (12.1-17.9) | 1035.7 (833.1-1225.5) | 34 (28.1-41.3) | 934.4 (772.1-1134.9) | -0.31 (-0.36--0.27) |
| Caribbean | 10.9 (8.9-13.4) | 741 (600.0-907.9) | 31.6 (26.5-37.1) | 986.4 (828.2-1160.0) | 0.97 (0.89-1.05) |
| Central Asia | 24.6 (19.9-29.6) | 1090.6 (884.2-1314.4) | 42.2 (35.4-50.5) | 1241.6 (1042.4-1484.7) | 0.6 (0.42-0.78) |
| Central Europe | 78.9 (62.1-96.3) | 999.1 (786.1-1219.7) | 176.7 (148.8-207.2) | 1190.6 (1002.4-1395.5) | 0.67 (0.61-0.73) |
| Central Latin America | 52.8 (44.1-61.1) | 1306.2 (1092.4-1512.0) | 218.6 (190.2-245.4) | 1600 (1392.3-1796.2) | 0.69 (0.63-0.75) |
| Central Sub-Saharan Africa | 7 (5.5-8.5) | 861 (679.4-1050.8) | 18.6 (15-22.6) | 984.8 (794.8-1199.7) | 0.41 (0.39-0.43) |
| East Asia | 768.5 (670.5-839.1) | 1974.8 (1723.0-2156.4) | 2182.4 (1880.7-2421.4) | 1766 (1521.9-1959.4) | -0.43 (-0.47--0.38) |
| Eastern Europe | 171.8 (137.4-209.4) | 1133.8 (907.2-1382.3) | 195.2 (157.3-236.9) | 913.9 (736.5-1109.2) | -0.77 (-0.99--0.55) |
| Eastern Sub-Saharan Africa | 22.2 (17.5-26.9) | 713.6 (561.9-865.0) | 49.3 (39.2-59.4) | 715.3 (568.6-862.1) | -0.08 (-0.15--0.01) |
| High-income Asia Pacific | 126.4 (100.6-153.2) | 1123.4 (893.4-1360.8) | 408.1 (340.7-489.9) | 1168 (975.2-1402.0) | 0.18 (0.13-0.23) |
| High-income North America | 261.2 (211.1-307.6) | 1124.6 (909.2-1324.5) | 589.6 (515.1-656.1) | 1361.5 (1189.3-1514.9) | 0.65 (0.45-0.85) |
| North Africa and Middle East | 68.2 (53.7-83.5) | 944.7 (743.9-1156.0) | 267.4 (218.8-316.3) | 1315 (1075.8-1555.7) | 1.18 (1.14-1.21) |
| Oceania | 1.9 (1.6-2.1) | 1790.7 (1561.1-1974.1) | 4.9 (4.4-5.3) | 1770.1 (1577.6-1937.5) | -0.06 (-0.10--0.02) |
| South Asia | 462.6 (409.5-502.6) | 1969.7 (1743.4-2139.8) | 1394.5 (1230.6-1519) | 1904.7 (1680.9-2074.7) | -0.14 (-0.15--0.13) |
| Southeast Asia | 132.5 (109.4-154.2) | 1214.2 (1001.8-1412.9) | 375.6 (307.4-441.4) | 1248.8 (1022.1-1467.6) | 0.05 (0.02-0.08) |
| Southern Latin America | 22 (17.5-27.4) | 836.4 (665.6-1040.0) | 52.3 (43.8-60.8) | 950.6 (797.4-1105.2) | 0.52 (0.38-0.65) |
| Southern Sub-Saharan Africa | 14 (11.5-16.7) | 1076.4 (884.1-1286.2) | 28.8 (23.4-34.5) | 1080.2 (875.7-1294.0) | -0.02 (-0.08-0.05) |
| Tropical Latin America | 65.3 (53.4-76) | 1496.2 (1222.3-1741.0) | 215.9 (179-250.9) | 1503.1 (1246.6-1746.8) | -0.1 (-0.14--0.05) |
| Western Europe | 548.1 (462.8-627.6) | 1467.6 (1239.1-1680.5) | 1048.6 (916.9-1179.3) | 1593.7 (1393.6-1792.4) | 0.37 (0.34-0.40) |
| Western Sub-Saharan Africa | 23.8 (18.9-29) | 592.4 (470.3-720.8) | 57.5 (45.6-69.8) | 705.5 (559.3-856.2) | 0.6 (0.58-0.62) |

SDI, Sociodemographic Index; EAPC, estimated annual percentage change; UI, uncertainty interval; CI, confidence interval.

**S2 Table. Death and EAPC of chronic obstructive pulmonary disease among adults aged ≥70 years at the global and regional levels, from 1990 to 2021**

|  | **Deaths (95% UI)** | | | |  |
| --- | --- | --- | --- | --- | --- |
|  | **No in 1990, thousands** | **Rate in 1990 (per 100,000)** | **No in 2021, thousands** | **Rate in 2021 (per 100,000)** | **EAPCs (95% CI)** |
| **Global** | 1671.2 (1495.5-1797.3) | 827.3 (740.3-889.7) | 2853.3 (2540.7-3145.8) | 577.2 (513.9-636.3) | -1.33 (-1.40--1.25) |
| **SDI** |  |  |  |  |  |
| High SDI | 221 (202.5-230.1) | 320.1 (293.2-333.2) | 385.4 (328.8-415.6) | 268.6 (229.2-289.6) | -0.58 (-0.67--0.49) |
| High-middle SDI | 486.6 (427.6-529.7) | 945.6 (830.9-1029.3) | 579.4 (494.6-659.3) | 493.5 (421.3-561.6) | -2.55 (-2.73--2.38) |
| Middle SDI | 633.8 (557-689) | 1387.1 (1219.0-1507.8) | 1019.2 (875.3-1163.1) | 722.9 (620.8-824.9) | -2.42 (-2.54--2.30) |
| Low-middle SDI | 255.8 (209.4-298.1) | 975.4 (798.5-1136.9) | 694.5 (619.9-768.9) | 990.8 (884.3-1096.9) | 0.20 (0.07-0.33) |
| Low SDI | 73 (59-85.4) | 782.3 (632.4-914.8) | 173.5 (154.6-196.3) | 790.9 (704.7-894.9) | 0.31 (0.13-0.49) |
| **Regions** |  |  |  |  |  |
| Andean Latin America | 2.6 (2.2-2.9) | 253.5 (218.8-285.3) | 6.3 (5.1-7.6) | 192.1 (157.0-232.7) | -0.37 (-0.52--0.23) |
| Australasia | 5.2 (4.8-5.5) | 356 (331.3-376.0) | 9.7 (8.4-10.6) | 267.1 (229.5-291.9) | -0.9 (-1.06--0.74) |
| Caribbean | 3 (2.7-3.3) | 204 (182.1-224.8) | 7.8 (6.8-8.7) | 242.4 (213.1-270.9) | 0.41 (0.21-0.61) |
| Central Asia | 9.1 (8.4-9.7) | 402.2 (371.4-428.9) | 8.5 (7.6-9.4) | 250.5 (224.9-275.6) | -1.64 (-2.01--1.27) |
| Central Europe | 27.8 (26.5-28.8) | 352.2 (335.9-365.2) | 27.7 (25.2-29.9) | 186.6 (169.7-200.5) | -1.56 (-1.83--1.30) |
| Central Latin America | 17.4 (16.2-18.0) | 430 (401.7-444.7) | 50.6 (44.4-55.7) | 370.4 (325.0-407.7) | -0.51 (-0.62--0.39) |
| Central Sub-Saharan Africa | 4 (2.9-5.4) | 493.4 (352.2-669.8) | 8.3 (5.7-12.9) | 438.8 (303.8-684.7) | -0.42 (-0.46--0.37) |
| East Asia | 884.6 (757.6-983.6) | 2273.2 (1947.0-2527.5) | 1132.5 (931.4-1334.7) | 916.4 (753.7-1080.1) | -3.39 (-3.57--3.20) |
| Eastern Europe | 57.5 (54.5-59.3) | 379.3 (359.7-391.1) | 27.5 (25.2-29.5) | 128.6 (117.8-138.3) | -3.94 (-4.27--3.62) |
| Eastern Sub-Saharan Africa | 13 (10-15.0) | 418.8 (322.7-482.7) | 22 (18.0-25.8) | 319.6 (261.6-373.9) | -1.07 (-1.15--0.99) |
| High-income Asia Pacific | 20.9 (18.8-22.3) | 185.8 (167.2-197.7) | 41.7 (34.1-46.5) | 119.4 (97.7-133.1) | -1.51 (-1.77--1.26) |
| High-income North America | 71.2 (64.3-74.5) | 306.6 (277.0-320.9) | 166 (140.2-178.3) | 383.3 (323.7-411.6) | 0.79 (0.47-1.11) |
| North Africa and Middle East | 27.6 (21.7-31.6) | 382.7 (300.0-438.1) | 626 (54.8-70.0) | 307.9 (269.7-344.4) | -0.33 (-0.54--0.12) |
| Oceania | 1.4 (1.1-1.8) | 1368.2 (1072.8-1715.4) | 3.4 (2.8-4.1) | 1222.6 (1007.2-1495.4) | -0.36 (-0.44--0.27) |
| South Asia | 285.4 (226.3-338.9) | 1215.2 (963.6-1442.9) | 857.1 (763.0-966.0) | 1170.6 (1042.1-1319.4) | 0.04 (-0.13-0.21) |
| Southeast Asia | 73 (59.7-82.6) | 668.9 (546.8-756.5) | 151 (134.6-169.4) | 502.2 (447.4-563.1) | -0.97 (-1.07--0.87) |
| Southern Latin America | 7.9 (7.4-8.4) | 300.7 (281.9-317.0) | 16.3 (14.6-17.5) | 296.1 (264.9-319.2) | 0.18 (-0.19-0.56) |
| Southern Sub-Saharan Africa | 5.4 (4.7-6.6) | 419.6 (365.0-507.7) | 10.2 (9.3-11.0) | 381.1 (350.5-412.1) | -0.43 (-0.78--0.09) |
| Tropical Latin America | 21.3 (19.5-22.5) | 489 (447.2-514.4) | 46.4 (40.3-50.0) | 323.1 (280.3-348.2) | -1.62 (-1.86--1.37) |
| Western Europe | 120.8 (111-125.9) | 323.4 (297.3-337.0) | 178.3 (151.5-192.5) | 271 (230.2-292.6) | -0.46 (-0.55--0.37) |
| Western Sub-Saharan Africa | 11.9 (9.4-14.0) | 296.5 (235.1-347.3) | 19.4 (17.2-21.9) | 237.7 (211.4-268.2) | -0.49 (-0.59--0.39) |

SDI, Sociodemographic Index; EAPC, estimated annual percentage change; UI, uncertainty interval; CI, confidence interval.

**S3 Table. DALYs and EAPC of chronic obstructive pulmonary disease among adults aged ≥70 years at the global and regional levels, from 1990 to 2021**

|  | **DALYs (95% UI)** | | | |  |
| --- | --- | --- | --- | --- | --- |
|  | **No in 1990, thousands** | **Rate in 1990 (per 100,000)** | **No in 2021, thousands** | **Rate in 2021 (per 100,000)** | **EAPCs (95% CI)** |
| **Global** | 27204.2 (24685.7-29148.3) | 13467 (12220.2-14429.4) | 45430.2 (41416.3-49191.5) | 9189.5 (8377.6-9950.4) | -1.41 (-1.48--1.34) |
| **SDI** |  |  |  |  |  |
| High SDI | 3872.2 (3620.5-4052.6) | 5607.3 (5242.8-5868.5) | 6590.5 (5908.1-7038.8) | 4593.3 (4117.7-4905.7) | -0.7 (-0.78--0.62) |
| High-middle SDI | 7566.1 (6728.2-8167.3) | 14702.3 (13074.1-15870.5) | 8744 (7659.4-9830.0) | 7448 (6524.1-8373.0) | -2.65 (-2.83--2.48) |
| Middle SDI | 10154.8 (8993.6-11013.3) | 22223.1 (19681.8-24101.7) | 15742.1 (13927.2-17733.7) | 11165.1 (9877.9-12577.6) | -2.52 (-2.63--2.41) |
| Low-middle SDI | 4324.8 (3591.1-4979.1) | 16491.6 (13693.8-18986.4) | 11376.6 (10300.8-12465.5) | 16230.2 (14695.5-17783.7) | 0.03 (-0.07-0.12) |
| Low SDI | 1271.9 (1061.2-1468.7) | 13629.4 (11372.0-15737.6) | 2955.6 (2661.4-3281.9) | 13475.2 (12133.8-14962.9) | 0.17 (0.04-0.29) |
| **Regions** |  |  |  |  |  |
| Andean Latin America | 38.7 (34.2-43.0) | 3792.1 (3346.8-4211.9) | 95.3 (79.4-111.6) | 2906.2 (2422.9-3403.0) | -0.48 (-0.58--0.37) |
| Australasia | 86.5 (80.9-91.5) | 5937.4 (5552.4-6275.7) | 146.3 (130.0-157.7) | 4015.6 (3566.8-4329.6) | -1.32 (-1.46--1.18) |
| Caribbean | 48.7 (43.4-53.2) | 3302.4 (2937.7-3607.3) | 121.9 (108.6-135.9) | 3810.4 (3393.9-4248.9) | 0.35 (0.18-0.52) |
| Central Asia | 142.4 (133.3-150.8) | 6320.7 (5915.1-6690.9) | 147.612 (133.7-162.0) | 4342.6 (3934.6-4765.2) | -1.38 (-1.65--1.12) |
| Central Europe | 456.4 (434.1-477.1) | 5778.1 (5495.0-6040.5) | 510.8 (468.3-552.7) | 3440.9 (3154.6-3723.4) | -1.35 (-1.53--1.16) |
| Central Latin America | 260.6 (246.6-270.1) | 6450.3 (6104.2-6686.4) | 750.4 (671.0-817.6) | 5492.9 (4911.6-5984.7) | -0.6 (-0.71--0.49) |
| Central Sub-Saharan Africa | 73.6 (54.4-96.5) | 9075.5 (6710.2-11904.7) | 149.6 (109.7-220.0) | 7936.6 (5817.4-11668.2) | -0.5 (-0.54--0.47) |
| East Asia | 13858.9 (11992.2-15302.4) | 35614.3 (30817.3-39324.0) | 16731.9 (14258.9-19542.8) | 13539.4 (11538.2-15813.9) | -3.55 (-3.73--3.38) |
| Eastern Europe | 937.6 (890.4-974.4) | 6189 (5877.1-6431.9) | 519.4 (475.1-560.2) | 2431.6 (2224.2-2622.4) | -3.5 (-3.74--3.26) |
| Eastern Sub-Saharan Africa | 234.1 (187-266.4) | 7518 (6003.9-8552.9) | 397.6 (334.6-458.6) | 5771.2 (4855.8-6656.2) | -1.05 (-1.13--0.97) |
| High-income Asia Pacific | 373.5 (340.2-401.1) | 3318.2 (3022.4-3563.3) | 761.8 (658.3-848.7) | 2180.4 (1884.0-2429.1) | -1.33 (-1.49--1.17) |
| High-income North America | 1407.9 (1299.5-1493.9) | 6063 (5596.0-6433.1) | 2980.1 (2680.2-3180.9) | 6881.2 (6188.7-7344.8) | 0.43 (0.18-0.69) |
| North Africa and Middle East | 461 (371.4-520.4) | 6383.1 (5142.4-7206.5) | 1083.4 (979.0-1202.2) | 5328.1 (4814.5-5912.3) | -0.37 (-0.51--0.24) |
| Oceania | 24.2 (19-30.2) | 23276.6 (18240.4-29039.8) | 54.4 (45.0-66.0) | 19732.7 (16309.0-23895.0) | -0.59 (-0.63--0.56) |
| South Asia | 4879.5 (3947.8-5698.0) | 20774.8 (16808.1-24259.5) | 14210.2 (12843.6-15710.5) | 19409 (17542.5-21458.2) | -0.13 (-0.25--0.00) |
| Southeast Asia | 1197.7 (996.8-1338.4) | 10971.5 (9131.0-12259.7) | 2486.6 (2236.2-2770.7) | 8267.4 (7434.6-9211.6) | -1.01 (-1.11--0.91) |
| Southern Latin America | 123.4 (116.4-130.4) | 4683.6 (4415.9-4948.0) | 233.7 (210.9-250.0) | 4252 (3836.6-4548.7) | -0.15 (-0.45-0.16) |
| Southern Sub-Saharan Africa | 96.2 (85.3-112.0) | 7410.5 (6569.9-8627.4) | 191.1 (175.4-206.4) | 7165.6 (6578.3-7738.0) | -0.25 (-0.55-0.06) |
| Tropical Latin America | 338 (316-354.0) | 7740.3 (7237.4-8107.6) | 724.2 (646.8-772.4) | 5042.2 (4503.1-5377.5) | -1.7 (-1.92--1.47) |
| Western Europe | 1950.8 (1831.4-2040.6) | 5223.7 (4903.9-5464.1) | 2768.3 (2456.9-2958.5) | 4207.2 (3734.1-4496.5) | -0.64 (-0.72--0.56) |
| Western Sub-Saharan Africa | 214.3 (174.5-247.6) | 5333.1 (4342.0-6161.9) | 365.3 (327.1-405.7) | 4482.4 (4014.2-4978.4) | -0.4 (-0.48--0.32) |

SDI, Sociodemographic Index; EAPC, estimated annual percentage change; UI, uncertainty interval; CI, confidence interval.

**S4 Table. Prevalence and EAPC of chronic obstructive pulmonary disease among adults aged ≥70 years in 204 countries and territories, from 1990 to 2021**

|  | **Prevalence (95% UI)** | | | |  |
| --- | --- | --- | --- | --- | --- |
|  | **No in 1990** | **Rate in 1990 (per 100,000)** | **No in 2021** | **Rate in 2021 (per 100,000)** | **EAPCs (95% CI)** |
| Afghanistan | 43669.8 (35905.1-51660.7) | 14052 (11553.5-16623.3) | 69404.9 (57616.9-83316.1) | 17156.4 (14242.5-20595.1) | 0.525 (0.466-0.584) |
| Albania | 17339 (14482.9-20436.4) | 17369.5 (14508.4-20472.3) | 54871.6 (46003.9-63480.4) | 20233.6 (16963.7-23408.1) | 0.614 (0.536-0.693) |
| Algeria | 81646.2 (68388.9-98216) | 13109.7 (10981-15770.3) | 336152.6 (281666.5-390445.8) | 19428.7 (16279.5-22566.7) | 1.259 (1.205-1.314) |
| American Samoa | 133.5 (109.9-157.3) | 15203.6 (12517.4-17914.6) | 307.2 (249.2-367.2) | 13874.4 (11253.1-16582.2) | -0.29 (-0.323--0.257) |
| Andorra | 680.5 (575.1-781) | 21246.1 (17955.4-24383.2) | 2107.7 (1820.9-2412.1) | 22584.2 (19511.5-25845.4) | 0.293 (0.195-0.391) |
| Angola | 13319.4 (10851.2-15841.4) | 10385.8 (8461.2-12352.3) | 43644.1 (35745-51227.7) | 10876 (8907.6-12765.8) | 0.052 (-0.029-0.133) |
| Antigua and Barbuda | 279.8 (227.4-330) | 7690.8 (6249.6-9072.5) | 569.5 (462.4-682.5) | 10139.9 (8231.8-12151.4) | 0.888 (0.799-0.977) |
| Argentina | 217700.8 (176584.4-265558.6) | 11772.4 (9549-14360.3) | 454742.4 (387367.5-527335.7) | 12760.5 (10869.9-14797.5) | 0.414 (0.259-0.569) |
| Armenia | 22574.4 (19038-26268.7) | 18896.7 (15936.4-21989.1) | 51269.3 (42733.8-60471.4) | 20971.4 (17480-24735.5) | 0.634 (0.358-0.912) |
| Australia | 208085.6 (173854.1-234806) | 17198 (14368.8-19406.4) | 435536.2 (372380.7-509289) | 14122.8 (12074.9-16514.3) | -0.586 (-0.705--0.467) |
| Austria | 172072.3 (151871.7-193707.2) | 22570 (19920.4-25407.7) | 307219.8 (269375.3-343724.2) | 24697.5 (21655.2-27632.1) | 0.345 (0.260-0.429) |
| Azerbaijan | 35569.5 (29227.5-41907.1) | 15833.9 (13010.7-18655.1) | 69583 (57121.7-82639.3) | 18159.1 (14907.1-21566.5) | 0.505 (0.277-0.733) |
| Bahrain | 944.6 (775.9-1116.4) | 16510.9 (13562.8-19513.5) | 4646.9 (3891.7-5416) | 18445.4 (15447.6-21498.4) | 0.409 (0.308-0.510) |
| Bangladesh | 471321.8 (408052.3-541768.5) | 20714.6 (17933.9-23810.7) | 1681887.5 (1460307.7-1900459.7) | 22821.8 (19815.2-25787.6) | 0.341 (0.281-0.401) |
| Barbados | 1645.6 (1357.8-1946.3) | 7700.3 (6353.2-9107) | 3331.1 (2750-3877.6) | 10394.7 (8581.3-12100) | 0.928 (0.852-1.004) |
| Belarus | 126444.2 (104728.2-147746.8) | 18174.8 (15053.4-21236.8) | 147491.6 (124746.8-171521.9) | 15457.7 (13073.9-17976.1) | -0.444 (-0.674--0.214) |
| Belgium | 184749.1 (161783.8-212008.8) | 19226.4 (16836.5-22063.3) | 338628 (294361.6-386757.5) | 21060.5 (18307.4-24053.9) | 0.449 (0.406-0.492) |
| Belize | 547.3 (449.5-648.3) | 10390 (8532.6-12306.9) | 1795 (1509.7-2091.1) | 12856.7 (10813-14977.3) | 0.821 (0.769-0.873) |
| Benin | 9191.1 (7572.6-10911) | 8959.7 (7382-10636.3) | 22547.4 (18787.2-26761.4) | 10074.5 (8394.4-11957.4) | 0.415 (0.336-0.493) |
| Bermuda | 323.8 (260.8-383.9) | 9319.5 (7506.9-11050.7) | 1156 (953.2-1394) | 12831.6 (10580.8-15472.5) | 1.035 (0.940-1.131) |
| Bhutan | 1885.4 (1627.6-2139.1) | 21027.7 (18152.3-23857.1) | 7236.3 (6219.4-8186.6) | 22446.8 (19292.5-25394.7) | 0.287 (0.266-0.307) |
| Bolivia | 20761.8 (17070.5-24641.5) | 13831.2 (11372.1-16415.8) | 72617.2 (59358.7-84658.7) | 15692.5 (12827.3-18294.6) | 0.529 (0.471-0.587) |
| Bosnia and Herzegovina | 31993.1 (26385.8-37885.1) | 19082.1 (15737.6-22596.3) | 83359.5 (71000.4-95442.2) | 21451.8 (18271.3-24561.2) | 0.619 (0.513-0.725) |
| Botswana | 2693.2 (2202.9-3155.2) | 11725.1 (9590.7-13736.4) | 7734.9 (6388.9-9036.7) | 13210.4 (10911.6-15433.7) | 0.398 (0.271-0.525) |
| Brazil | 807036.7 (667583-937862.6) | 18987.7 (15706.6-22065.7) | 2953925.3 (2484365-3411173.4) | 21007.3 (17667.9-24259) | 0.218 (0.173-0.262) |
| Brunei | 899.6 (774.2-1020.1) | 20198.8 (17382.6-22904.7) | 1956.4 (1651-2294.4) | 14337 (12099.2-16814.4) | -1.295 (-1.409--1.180) |
| Bulgaria | 114922.5 (96771.7-132501.9) | 17425.6 (14673.4-20091.1) | 193049.9 (161946.9-225959.8) | 18753.3 (15731.9-21950.3) | 0.385 (0.291-0.480) |
| Burkina Faso | 15509.6 (12619.5-18449.4) | 7900.1 (6428-9397.6) | 39435.4 (32890.6-47184.9) | 9362.7 (7808.8-11202.5) | 0.539 (0.489-0.589) |
| Burundi | 10779.3 (8744.1-12954.8) | 9526.8 (7728.1-11449.6) | 20175.5 (16588.5-24034.9) | 11199.7 (9208.5-13342.1) | 0.399 (0.322-0.476) |
| Cambodia | 25553.4 (20962.1-30438.4) | 13702.4 (11240.5-16321.9) | 84134.4 (68814.9-98879.3) | 15379.4 (12579.1-18074.7) | 0.732 (0.547-0.918) |
| Cameroon | 14851.3 (12216.8-17756.2) | 8066.9 (6636-9644.8) | 45207.6 (36743.1-53413.4) | 9036.2 (7344.3-10676.4) | 0.404 (0.359-0.448) |
| Canada | 356469.9 (297865.6-415557.6) | 17723.1 (14809.4-20660.9) | 926367 (800056-1050028.5) | 19224.1 (16602.8-21790.3) | 0.327 (0.200-0.453) |
| Cape Verde | 785.8 (639.4-944.6) | 5255.5 (4276.4-6317.7) | 1325.3 (1084.4-1575.7) | 6469.9 (5294.2-7692.4) | 0.109 (-0.242-0.462) |
| Central African Republic | 4132.2 (3398.3-4928.4) | 11121.7 (9146.5-13264.6) | 8461 (6914.5-10104.4) | 13260.9 (10837.2-15836.6) | 0.525 (0.469-0.580) |
| Chad | 13761 (11190.9-16823.2) | 9136.8 (7430.4-11170) | 25531.6 (20754.9-30015.7) | 10713.1 (8708.8-12594.7) | 0.511 (0.418-0.603) |
| Chile | 60779.6 (50234.7-72737.4) | 11249.2 (9297.5-13462.4) | 174913.8 (146767.3-203889.1) | 11229.4 (9422.4-13089.6) | -0.002 (-0.058-0.053) |
| China | 7419682.4 (6442718.7-8299028.8) | 19770.2 (17167-22113.3) | 24270972.6 (20647891.2-27881420.1) | 20343.8 (17306.9-23370) | 0.194 (0.131-0.256) |
| Colombia | 145503.7 (127588-162665.5) | 17394.5 (15252.7-19446.1) | 697438.5 (613244.8-779051.9) | 21885.4 (19243.5-24446.4) | 0.659 (0.610-0.709) |
| Comoros | 686.6 (558.2-809.9) | 8474.2 (6889.3-9995.4) | 2096.4 (1730.1-2507.6) | 9239.7 (7625.2-11051.9) | 0.21 (0.120-0.300) |
| Congo | 4102.1 (3349.1-4955.3) | 9407.9 (7680.9-11364.8) | 10886.2 (8819.4-13005.6) | 11319.4 (9170.4-13523.2) | 0.577 (0.496-0.657) |
| Cook Islands | 85.4 (70.4-101.4) | 14523.9 (11976.4-17240.5) | 223 (179.5-268.2) | 14319.9 (11524.4-17221.2) | -0.128 (-0.215--0.041) |
| Costa Rica | 15403.4 (12935.7-18089.3) | 16261.6 (13656.4-19097.1) | 63810.6 (54839.2-72740.4) | 20347.7 (17487-23195.2) | 0.719 (0.673-0.765) |
| Cote d'Ivoire | 10940.8 (8976.9-13126) | 8176.7 (6709-9809.9) | 40561.5 (33216.7-49230.6) | 9727.9 (7966.4-11807) | 0.675 (0.560-0.791) |
| Croatia | 47761.6 (39841.8-55837.3) | 16850.5 (14056.3-19699.6) | 136530.1 (119012.9-154702.7) | 22206.2 (19357.1-25162) | 1.22 (1.105-1.335) |
| Cuba | 75315.8 (62884.2-88479.5) | 12036 (10049.3-14139.7) | 210813.3 (179981.8-245773.4) | 16977.2 (14494.3-19792.6) | 1.195 (1.056-1.333) |
| Cyprus | 9764.9 (8199.2-11475.3) | 18935 (15899-22251.6) | 27577.5 (23435.8-31482.7) | 20369.8 (17310.6-23254.4) | 0.214 (0.118-0.310) |
| Czech Republic | 119938.5 (98134.9-141846.5) | 15048.9 (12313.2-17797.7) | 307233.3 (262942.3-345959.7) | 19893.5 (17025.7-22401.1) | 1.122 (1.036-1.208) |
| Democratic Republic of the Congo | 57902.6 (47105-69112.5) | 10232.5 (8324.3-12213.5) | 169722.2 (139671.9-202994) | 13422.7 (11046.1-16054) | 0.886 (0.856-0.916) |
| Denmark | 122705.3 (106362.7-139823) | 21951.1 (19027.5-25013.3) | 188776.4 (162143.3-212264.7) | 21958.8 (18860.8-24691) | -0.163 (-0.246--0.079) |
| Djibouti | 309 (254-375.4) | 7269.8 (5975.9-8831.6) | 1812 (1477.2-2167) | 8273.6 (6745-9894.6) | 0.327 (0.156-0.499) |
| Dominica | 345.9 (282.5-413) | 8969.3 (7326.9-10709.4) | 493.7 (404.3-587.8) | 10940.9 (8959-13025.8) | 0.601 (0.564-0.637) |
| Dominican Republic | 16985.1 (13862.4-20463.1) | 9211.9 (7518.3-11098.1) | 71575.6 (59491.3-85056.1) | 12898.4 (10720.7-15327.7) | 1.097 (1.043-1.151) |
| Ecuador | 33783.5 (27763.8-40264.3) | 12474.9 (10252.1-14868) | 143536 (120826-169113.4) | 15353.2 (12924-18089) | 0.687 (0.652-0.721) |
| Egypt | 110694.5 (89868-132042.6) | 11261.7 (9142.9-13433.6) | 373262.2 (308495.3-442308.8) | 16441.4 (13588.6-19482.8) | 1.183 (1.134-1.231) |
| El Salvador | 21225.9 (17602.5-24929) | 13396.5 (11109.7-15733.8) | 68517.2 (57346.3-80826.9) | 17893 (14975.8-21107.7) | 0.973 (0.940-1.007) |
| Equatorial Guinea | 812.6 (667.1-960.3) | 10829.7 (8890.7-12798.3) | 2290.6 (1846.6-2740.9) | 11964.6 (9645.6-14316.5) | 0.364 (0.185-0.542) |
| Eritrea | 2216.5 (1813.7-2659.5) | 7870.3 (6440.3-9443.5) | 8025.4 (6542.5-9685.1) | 8742 (7126.8-10550) | 0.254 (0.140-0.369) |
| Estonia | 10728.1 (9019.8-12618) | 9041.6 (7601.8-10634.3) | 23876.8 (20422.4-27503.6) | 12478.4 (10673.1-14373.8) | 1.485 (1.349-1.620) |
| Ethiopia | 77706.6 (63357.8-93453) | 10054.5 (8197.9-12092) | 209859 (172767.6-246406.9) | 10644.2 (8762.9-12498) | 0.084 (-0.033-0.201) |
| Federated States of Micronesia | 388.3 (318.1-461.7) | 16531.4 (13544-19658.4) | 362.8 (299.1-435.1) | 14360.2 (11839.1-17224.4) | -0.393 (-0.425--0.361) |
| Fiji | 1912.6 (1567.1-2284.2) | 14143.3 (11588.2-16891.6) | 3587.8 (2941.3-4275.6) | 11151.3 (9142-13289.2) | -0.861 (-0.924--0.799) |
| Finland | 67964 (60082.8-76803.6) | 14860.2 (13137-16792.9) | 164591.9 (140917.6-192592) | 17568.4 (15041.4-20557.1) | 0.487 (0.418-0.555) |
| France | 951069.7 (802020.4-1092618.6) | 18278.4 (15413.8-20998.8) | 1878768.5 (1651633.4-2154498) | 18915.4 (16628.6-21691.4) | 0.321 (0.213-0.429) |
| Gabon | 2421.9 (1954-2869.6) | 8581.5 (6923.5-10167.6) | 4249 (3450.9-5111.3) | 10486.3 (8516.5-12614.4) | 0.681 (0.534-0.829) |
| Georgia | 43052.5 (35761.9-50537.9) | 13041.8 (10833.3-15309.4) | 63734.8 (52827-75186.2) | 17086.8 (14162.5-20156.8) | 0.983 (0.800-1.166) |
| Germany | 1860461.9 (1598766-2127405.4) | 23013.1 (19776-26315.1) | 3302061.2 (2909873.4-3687975.4) | 24359.6 (21466.4-27206.6) | 0.261 (0.054-0.468) |
| Ghana | 19845.3 (16397.3-23821.8) | 7831.4 (6470.8-9400.7) | 68223.3 (56058.2-81408.6) | 9910.3 (8143.2-11825.6) | 0.839 (0.785-0.893) |
| Greece | 190306.8 (159255.8-221303.3) | 20088.9 (16811.1-23360.9) | 407483.8 (355607.8-466824.2) | 23536.6 (20540.2-26964.2) | 0.517 (0.414-0.620) |
| Greenland | 274.7 (242.3-307) | 22132.9 (19523.4-24735.2) | 630.7 (556.2-707.5) | 20972.4 (18492.4-23525.2) | -0.157 (-0.179--0.135) |
| Grenada | 453.5 (378-539.6) | 9115.1 (7597.8-10845.5) | 627.3 (527-729.3) | 10612.1 (8914.5-12337.8) | 0.274 (0.131-0.416) |
| Guam | 350 (276.6-431.5) | 12572.2 (9935.4-15498.8) | 1646.1 (1358.8-1939.7) | 13452.7 (11104.8-15852.3) | 0.393 (0.341-0.444) |
| Guatemala | 18749.9 (15449-22301.2) | 12779.2 (10529.4-15199.5) | 95624.3 (79721.4-112129.2) | 16222.2 (13524.3-19022.2) | 0.846 (0.821-0.871) |
| Guinea | 14462.2 (11752.3-17228.2) | 8721.9 (7087.6-10390) | 28093.9 (22838.5-33164.3) | 10825.4 (8800.4-12779.2) | 0.833 (0.743-0.922) |
| Guinea-Bissau | 1362.5 (1123.3-1659) | 8330.8 (6867.9-10143.7) | 2402.8 (1929.6-2870.3) | 9465.6 (7601.4-11307.2) | 0.433 (0.311-0.555) |
| Guyana | 1375 (1123.4-1633.9) | 7835.2 (6401.8-9310.5) | 2836.4 (2323.6-3319.1) | 9802.7 (8030.5-11471.2) | 0.733 (0.712-0.753) |
| Haiti | 14567.1 (11838.1-17381) | 11015.3 (8951.7-13143.1) | 36573.2 (30044.9-43230.1) | 12868.3 (10571.3-15210.5) | 0.466 (0.408-0.524) |
| Honduras | 15224.3 (12413.5-17955.1) | 15602.9 (12722.3-18401.7) | 62270.8 (51719.8-73226.1) | 19759.9 (16411.8-23236.3) | 0.715 (0.660-0.769) |
| Hungary | 154377.6 (127444.8-180608.5) | 18209.5 (15032.7-21303.6) | 305779.5 (265011.9-345879.5) | 22704.5 (19677.5-25682) | 0.908 (0.848-0.969) |
| Iceland | 4565.7 (3988.4-5149.7) | 24988.5 (21829.2-28185.1) | 8973.7 (7733.5-10139.7) | 23719 (20441-26801) | -0.193 (-0.358--0.029) |
| India | 3817123.6 (3340953.4-4271306.1) | 21192.4 (18548.7-23714) | 13871313.1 (12096354.3-15354507.7) | 23280.3 (20301.4-25769.6) | 0.361 (0.337-0.385) |
| Indonesia | 486701.8 (397693.5-574942.3) | 12780.8 (10443.4-15098) | 1487122.2 (1204091.8-1749065.6) | 15417.1 (12482.9-18132.7) | 0.609 (0.594-0.625) |
| Iran | 95274.2 (78635.2-112843.7) | 9819 (8104.2-11629.7) | 592441.7 (497588.2-695729.5) | 16223.9 (13626.4-19052.4) | 1.912 (1.827-1.997) |
| Iraq | 44417.8 (36362.1-52634.3) | 11119 (9102.4-13175.8) | 138773.7 (116070.2-164536.1) | 13991.9 (11702.8-16589.4) | 0.861 (0.785-0.936) |
| Ireland | 56992.2 (48423.5-63981) | 21209.9 (18021-23810.8) | 111395.6 (96633.2-126986.7) | 21428.4 (18588.7-24427.5) | 0.06 (0.011-0.109) |
| Israel | 59256.4 (50487.7-68359.2) | 19491.2 (16606.9-22485.4) | 157314.4 (132688-181793.3) | 19054.1 (16071.3-22019) | 0.07 (-0.002-0.142) |
| Italy | 1040605.9 (864207.7-1210173.9) | 18786.5 (15601.9-21847.8) | 2227962.2 (1894875.2-2553690) | 21185.3 (18018-24282.5) | 0.465 (0.428-0.503) |
| Jamaica | 12037.8 (9991.9-14094.6) | 10347.6 (8589-12115.5) | 24076 (20872.6-27892.5) | 13237.3 (11476.1-15335.8) | 0.865 (0.774-0.956) |
| Japan | 1424946.8 (1171156.1-1684406) | 14426.6 (11857.1-17053.4) | 4059349.4 (3369218.2-4822024.7) | 14045.2 (11657.3-16684) | 0.031 (-0.036-0.098) |
| Jordan | 6470.4 (5357.3-7583.9) | 13192.6 (10923.1-15462.9) | 47200 (39429.7-55373.6) | 15369.2 (12839-18030.7) | 0.493 (0.436-0.550) |
| Kazakhstan | 104102.5 (85926-124428.3) | 17351.2 (14321.7-20739) | 170712 (145796.3-195887.9) | 20990.1 (17926.6-24085.6) | 0.923 (0.637-1.210) |
| Kenya | 31039 (25507.3-37044.8) | 8376.8 (6883.9-9997.6) | 87927.5 (72017.4-104241.4) | 9423.2 (7718.1-11171.5) | 0.254 (0.158-0.350) |
| Kiribati | 218.4 (176.7-263.6) | 15147.3 (12256.6-18278.5) | 370.2 (304.1-440.3) | 14876.1 (12219.3-17694.6) | -0.007 (-0.053-0.040) |
| Kuwait | 2240.7 (1880-2642) | 11221.7 (9414.9-13231) | 16621 (13777.2-19465.3) | 16834.5 (13954.2-19715.3) | 1.485 (1.348-1.622) |
| Kyrgyzstan | 32410.5 (27808.5-36767.1) | 22412.1 (19229.8-25424.7) | 40320.9 (33701.7-46682.1) | 21007 (17558.4-24321.2) | -0.139 (-0.300-0.022) |
| Laos | 13695.4 (11350.9-16184.3) | 15433.3 (12791.2-18237.9) | 32303 (26799.3-38193.9) | 16769.4 (13912.3-19827.5) | 0.231 (0.191-0.272) |
| Latvia | 25566.8 (20904.6-30448.9) | 12322.6 (10075.6-14675.7) | 39759 (33587-46278.6) | 14119.9 (11928-16435.3) | 0.499 (0.247-0.751) |
| Lebanon | 11949.9 (9992.1-14247.7) | 12495.3 (10448.1-14898) | 77540.8 (64916.9-91635.3) | 20164.7 (16881.8-23830) | 1.792 (1.713-1.872) |
| Lesotho | 5697.5 (4646.6-6760.7) | 13281.9 (10832.1-15760.5) | 6862.6 (5583.4-8210.5) | 14273.9 (11613.3-17077.5) | 0.165 (0.076-0.255) |
| Liberia | 4814.3 (3936.2-5730.2) | 8091.6 (6615.7-9630.9) | 8906.7 (7351.1-10535.8) | 11168.4 (9217.7-13211.1) | 0.959 (0.888-1.029) |
| Libya | 10314.9 (8508.1-12265.4) | 11868.9 (9789.9-14113.2) | 35990.3 (29533.2-42643.2) | 16423.2 (13476.7-19459.1) | 1.144 (1.108-1.180) |
| Lithuania | 38145.1 (31458.5-45020.5) | 15029.3 (12394.8-17738.2) | 59308.9 (51343-67917.4) | 14766.7 (12783.3-16910) | -0.07 (-0.279-0.139) |
| Luxembourg | 7119 (6207-8105.8) | 20846.5 (18175.9-23735.9) | 14816 (12789.7-16975.4) | 22292.8 (19243.8-25541.8) | 0.246 (0.217-0.274) |
| Macedonia | 15919.2 (13181.1-18887.7) | 18063.4 (14956.5-21431.8) | 37915.4 (31778.5-44663.4) | 19956.2 (16726.1-23507.9) | 0.531 (0.443-0.620) |
| Madagascar | 19661.3 (16044.5-23574.2) | 9158.8 (7474-10981.6) | 40199.2 (33367.6-47835.8) | 11051.9 (9173.7-13151.4) | 0.551 (0.515-0.588) |
| Malawi | 10884.5 (8974.5-13040.7) | 6998.4 (5770.3-8384.7) | 27289.4 (22657.5-32090.5) | 8772.2 (7283.3-10315.5) | 0.704 (0.657-0.752) |
| Malaysia | 55405.4 (45771.6-65783.5) | 12732.6 (10518.7-15117.5) | 181186.7 (149157.1-215582.7) | 12813 (10548-15245.4) | -0.109 (-0.168--0.049) |
| Maldives | 473.3 (383.5-564.8) | 15776.3 (12783.3-18823.9) | 2591.4 (2145.1-3005.2) | 19052.8 (15771.6-22095.8) | 0.513 (0.372-0.654) |
| Mali | 12714.8 (10487.6-15284.4) | 7824.8 (6454.1-9406.2) | 35964.2 (29605.5-42793.6) | 9741.5 (8019.2-11591.4) | 0.714 (0.611-0.818) |
| Malta | 4393.6 (3765.6-4968.2) | 17696.5 (15167-20010.8) | 12326.5 (10492.1-14240.7) | 17169.8 (14614.6-19836.2) | -0.128 (-0.194--0.062) |
| Marshall Islands | 108.4 (87.8-130.9) | 14764.4 (11956.9-17827.3) | 137.6 (111.9-166.1) | 11986 (9747.1-14469.3) | -0.703 (-0.740--0.666) |
| Mauritania | 3709.4 (3031.3-4450) | 7109.2 (5809.8-8528.8) | 9367 (7720.9-11213.4) | 9201.6 (7584.6-11015.4) | 0.808 (0.718-0.899) |
| Mauritius | 3509 (2909.3-4075.1) | 10655.8 (8834.6-12374.7) | 12342.5 (10623.2-14223.7) | 12308.2 (10593.6-14184.1) | 0.417 (0.386-0.448) |
| Mexico | 335283.4 (276380.9-393824.4) | 16144.3 (13308.1-18963.2) | 1332906.5 (1125773.3-1547069.4) | 19727 (16661.4-22896.6) | 0.665 (0.573-0.758) |
| Moldova | 34099.7 (28820.8-39460.9) | 16234.4 (13721.2-18786.8) | 54206.7 (45220.1-64275.1) | 15555.1 (12976.3-18444.3) | -0.149 (-0.282--0.016) |
| Monaco | 1017.9 (867.8-1187.6) | 20543.7 (17515.9-23970.5) | 1447.4 (1231.3-1692.9) | 20331.6 (17296.4-23780.1) | 0.053 (-0.073-0.180) |
| Mongolia | 7822.2 (6446.9-9490.9) | 15222 (12545.7-18469.2) | 14068.9 (11695.9-16631) | 16721.4 (13901.1-19766.6) | 0.293 (0.189-0.397) |
| Montenegro | 4464.7 (3722.2-5282.3) | 13950.7 (11630.6-16505.4) | 9308.7 (7711.3-10818.2) | 16001.1 (13255.3-18595.8) | 0.697 (0.587-0.808) |
| Morocco | 77867.5 (63521.4-92359.4) | 10977.1 (8954.7-13020.1) | 276901.1 (228095.6-327361.2) | 16755.9 (13802.6-19809.4) | 1.375 (1.333-1.418) |
| Mozambique | 19094.6 (15593.3-23142.8) | 7856.8 (6416.1-9522.5) | 38901.3 (31810.5-45819.8) | 9049.3 (7399.8-10658.7) | 0.324 (0.258-0.390) |
| Myanmar | 181600.8 (152524.3-210513.8) | 18567.3 (15594.5-21523.5) | 445102.3 (375575.1-506434.3) | 19917.8 (16806.6-22662.4) | 0.299 (0.258-0.339) |
| Namibia | 3171.5 (2599.8-3812.2) | 11943.5 (9790.6-14356.3) | 8003.8 (6594.8-9538.2) | 13369.6 (11015.9-15932.6) | 0.411 (0.318-0.505) |
| Nauru | 22.8 (18.5-27.4) | 14429.4 (11735.8-17338) | 30.6 (24.6-37.2) | 13510.4 (10859.6-16463.3) | -0.229 (-0.250--0.209) |
| Nepal | 76293.4 (67761.1-83656.3) | 20924 (18583.9-22943.3) | 261227.8 (242581.3-277064.3) | 22422.6 (20822-23781.9) | 0.329 (0.269-0.390) |
| Netherlands | 218963.2 (190422.9-246601.9) | 17069 (14844.1-19223.5) | 441955.6 (384505.3-503254.8) | 17995.2 (15656-20491.1) | 0.586 (0.374-0.799) |
| New Zealand | 39923.6 (32353-47972.8) | 16135.3 (13075.6-19388.4) | 83464.4 (66736.9-101937.6) | 14918.7 (11928.8-18220.6) | -0.142 (-0.290-0.007) |
| Nicaragua | 10886.5 (9030-12920.7) | 14792.1 (12269.6-17556.1) | 50242.3 (43485.5-57704.4) | 20122.4 (17416.3-23111) | 1.048 (0.952-1.143) |
| Niger | 8948.4 (7308.9-10767) | 8704.3 (7109.4-10473.3) | 38180.5 (31353.8-45516.7) | 11205.6 (9202-13358.7) | 0.952 (0.857-1.047) |
| Nigeria | 182493.9 (150657.1-216602.3) | 8710.3 (7190.7-10338.3) | 421054.3 (347560.3-491858.9) | 11245.2 (9282.4-13136.2) | 0.898 (0.842-0.954) |
| Niue | 24 (19.4-28.2) | 16346.7 (13237.2-19209.5) | 16.8 (13.8-20.6) | 14180.7 (11594.3-17321.6) | -0.507 (-0.612--0.403) |
| North Korea | 151092 (135229.6-166670.5) | 23076 (20653.3-25455.2) | 369265.3 (318570.2-422178.9) | 21100 (18203.2-24123.5) | -0.353 (-0.377--0.329) |
| Northern Mariana Islands | 64.6 (51.5-78.1) | 13444.3 (10725.6-16260.8) | 271.5 (220.4-329.8) | 12820 (10408.8-15572.2) | -0.03 (-0.147-0.086) |
| Norway | 95757.9 (81129.9-110208.9) | 19598.7 (16604.8-22556.4) | 167363.8 (142331.8-190203.4) | 24175.6 (20559.8-27474.8) | 0.822 (0.593-1.052) |
| Oman | 3810.5 (3134.4-4558.1) | 13203.6 (10860.8-15793.8) | 11912.1 (9799.4-14105.6) | 17252.4 (14192.5-20429.3) | 0.771 (0.705-0.837) |
| Pakistan | 549059.5 (460568.8-638718.2) | 19421.8 (16291.6-22593.3) | 1015788.4 (842567.6-1175472.2) | 20060.1 (16639.3-23213.6) | 0.132 (0.081-0.183) |
| Palau | 68.5 (55.3-82.1) | 14325.2 (11563.3-17189.2) | 114.3 (92.9-137.9) | 12541.3 (10192.2-15123.5) | -0.394 (-0.499--0.288) |
| Palestine | 5401.8 (4448.3-6441.9) | 12808.2 (10547.4-15274.4) | 16051 (13402.8-18947.7) | 15461.6 (12910.7-18252.1) | 0.706 (0.605-0.808) |
| Panama | 10629.8 (8762.4-12521.7) | 13172.6 (10858.5-15517.1) | 46023.4 (39981.8-52016.4) | 17635.1 (15320.1-19931.5) | 0.803 (0.689-0.918) |
| Papua New Guinea | 11177.1 (9737.2-12540.8) | 18616.4 (16218-20887.8) | 31958.3 (27706.2-36115) | 18363.7 (15920.4-20752.2) | -0.024 (-0.094-0.047) |
| Paraguay | 17259.3 (14220-20411.9) | 14884.2 (12263.1-17603) | 54206.8 (45921.8-62877.9) | 17955.8 (15211.4-20828.1) | 0.645 (0.619-0.670) |
| Peru | 64417.1 (53437.4-77619.3) | 10744.6 (8913.2-12946.7) | 257041.9 (215474.9-303443.6) | 13653 (11445.1-16117.7) | 1.007 (0.895-1.119) |
| Philippines | 223202.7 (184873.4-260147) | 17223 (14265.4-20073.7) | 553063.8 (460074.6-644945) | 15288.7 (12718.2-17828.7) | -0.671 (-0.772--0.571) |
| Poland | 391878.2 (325090-456494.9) | 16285.4 (13509.8-18970.7) | 851826.3 (716673.4-978411.9) | 18237.5 (15343.9-20947.7) | 0.501 (0.420-0.582) |
| Portugal | 167484.9 (148736.6-184180.5) | 19483.9 (17302.8-21426.1) | 365492.5 (318629.7-418411.6) | 20654.5 (18006.2-23645) | 0.191 (0.162-0.220) |
| Puerto Rico | 25513.9 (21241.1-29693.8) | 11414.9 (9503.3-13285) | 82122 (69464.9-93104.9) | 15591.6 (13188.5-17676.8) | 0.909 (0.785-1.034) |
| Qatar | 335.8 (278.2-396.6) | 13911.1 (11525.6-16430.7) | 3210.4 (2719.4-3763.3) | 18281.2 (15485.2-21429.4) | 0.972 (0.894-1.050) |
| Romania | 266949.5 (222268-317339.8) | 18707.6 (15576.4-22238.9) | 478912.7 (410026.3-550010.7) | 18918.6 (16197.3-21727.2) | 0.149 (0.021-0.277) |
| Russian Federation | 1583507.5 (1305529.7-1856822.7) | 16399.6 (13520.7-19230.1) | 2211893 (1858485.3-2555694.1) | 15459.9 (12989.8-17862.9) | -0.217 (-0.372--0.062) |
| Rwanda | 10316.4 (8316-12379.7) | 9342.8 (7531.2-11211.4) | 27363.6 (22830-32019.8) | 11074.3 (9239.5-12958.6) | 0.485 (0.396-0.574) |
| Saint Kitts and Nevis | 206.3 (170.6-240.7) | 7632.6 (6310.7-8905.7) | 299.5 (248.1-350.9) | 10778.2 (8929.4-12627.1) | 1.205 (1.002-1.408) |
| Saint Lucia | 546.9 (452.6-650.3) | 10462.2 (8657.9-12440.5) | 1809.9 (1499.8-2094.2) | 13324.6 (11041.3-15417.5) | 0.844 (0.764-0.924) |
| Saint Vincent and the Grenadines | 330.2 (269.9-388.5) | 7568.8 (6185-8903.5) | 790.4 (668.7-920.8) | 9934.9 (8404.8-11572.9) | 0.948 (0.929-0.967) |
| Samoa | 675.7 (568-793.5) | 17831 (14989-20941.7) | 1117.7 (915.7-1318.4) | 16359.1 (13402.8-19296.8) | -0.2 (-0.234--0.167) |
| San Marino | 446.7 (376.7-520.3) | 19124.3 (16129.9-22276) | 1045.1 (877.4-1218.6) | 20060.5 (16840.9-23390) | 0.183 (0.108-0.258) |
| Sao Tome and Principe | 327 (270.3-388.2) | 9896.8 (8181.5-11751.5) | 576.7 (480.4-678.3) | 12580.7 (10478.4-14795.8) | 0.806 (0.783-0.828) |
| Saudi Arabia | 24212.1 (19818.7-29015.4) | 9753.3 (7983.5-11688.2) | 72472.5 (60732.3-85518) | 13964.6 (11702.4-16478.3) | 1.328 (1.239-1.418) |
| Senegal | 12720.3 (10483.1-14921) | 8411.5 (6932.2-9866.8) | 35548.9 (29380.8-42112.4) | 10040.1 (8298-11893.8) | 0.667 (0.573-0.761) |
| Serbia | 82643.1 (70248.4-96409.4) | 16639.7 (14144.1-19411.5) | 237726.6 (201652.4-271494.6) | 21987.8 (18651.2-25111) | 1.198 (1.115-1.281) |
| Seychelles | 372.9 (303.1-449.8) | 10900.2 (8858.4-13146.4) | 750 (615.5-905.9) | 13580.9 (11145.1-16403.6) | 0.595 (0.549-0.641) |
| Sierra Leone | 9472.1 (7796.3-11368.9) | 8767.9 (7216.7-10523.6) | 17763.5 (14735.6-21374.4) | 10359.7 (8593.8-12465.6) | 0.479 (0.415-0.543) |
| Singapore | 12455.3 (10220.9-14835.1) | 11899.7 (9764.9-14173.2) | 40700.2 (34116.1-48007.4) | 8423.3 (7060.7-9935.6) | -1.208 (-1.313--1.102) |
| Slovakia | 46023.6 (38459.2-54674.7) | 13922.2 (11634-16539.2) | 99410.7 (85008.9-115867.8) | 16535.1 (14139.6-19272.4) | 0.799 (0.723-0.874) |
| Slovenia | 23119.7 (19458.7-26887.2) | 16882.6 (14209.3-19633.8) | 58496.4 (50108.6-68175.9) | 19467.9 (16676.4-22689.3) | 0.621 (0.528-0.714) |
| Solomon Islands | 819.4 (693.2-960.5) | 17454.8 (14766.2-20461.4) | 2214.2 (1850.8-2611.4) | 16707.5 (13965.5-19704.8) | -0.106 (-0.136--0.076) |
| Somalia | 7210.1 (5859.7-8683.7) | 10274.1 (8349.8-12373.9) | 22923.9 (18796-27350.9) | 12032.4 (9865.7-14356) | 0.426 (0.316-0.536) |
| South Africa | 152388.6 (128031.7-177621.3) | 15019.9 (12619.2-17506.9) | 343193.8 (285582.8-398980.9) | 15511.8 (12907.9-18033.3) | 0.092 (0.062-0.122) |
| South Korea | 214609.3 (178397.7-250903.8) | 16906.7 (14054-19765.9) | 1082247.4 (958960.7-1202029.3) | 19534.5 (17309.2-21696.5) | 0.571 (0.399-0.744) |
| South Sudan | 10304 (8523.1-12322.3) | 8199.4 (6782.3-9805.5) | 13528.3 (10905.7-16169.7) | 9718.7 (7834.6-11616.2) | 0.387 (0.317-0.457) |
| Spain | 749068.4 (662800.6-845161.8) | 21733.2 (19230.2-24521.2) | 1626246.1 (1429387.8-1836589.8) | 24033.2 (21123.9-27141.7) | 0.38 (0.342-0.418) |
| Sri Lanka | 58217.4 (47094.1-70186.6) | 11805.4 (9549.8-14232.5) | 213074.4 (175450.8-254322.8) | 14004.3 (11531.5-16715.3) | 0.468 (0.414-0.522) |
| Sudan | 65832.1 (54231.5-78986.5) | 13968.6 (11507.1-16759.8) | 144389 (120227.1-170565.7) | 16864.3 (14042.3-19921.7) | 0.505 (0.437-0.573) |
| Suriname | 1199.6 (1007.9-1404.9) | 9821.1 (8251.5-11501.3) | 3899 (3241.9-4647.6) | 11718.7 (9743.5-13968.5) | 0.541 (0.521-0.561) |
| Swaziland | 1373.8 (1122.1-1645.7) | 11620 (9491-13919.9) | 2613.5 (2153.6-3127.2) | 11624.2 (9578.8-13909.1) | -0.141 (-0.269--0.014) |
| Sweden | 278605.6 (242595.4-316642.3) | 25524.1 (22225.1-29008.8) | 442574.4 (384168.8-496918.7) | 27750.7 (24088.5-31158.3) | 0.343 (0.161-0.525) |
| Switzerland | 151850.5 (131560.5-173019.2) | 21769.6 (18860.8-24804.4) | 278998 (243190.1-316573.7) | 22362.3 (19492.2-25374.1) | 0.011 (-0.106-0.128) |
| Syria | 28439.5 (23642-33508) | 12765.4 (10612-15040.4) | 99868 (83582-117896.8) | 16912.1 (14154.2-19965.2) | 1.059 (1.002-1.116) |
| Taiwan | 108702.7 (93248.4-124970.7) | 14904.9 (12785.9-17135.5) | 343729.6 (293044.7-409050.8) | 13610.3 (11603.4-16196.8) | -0.176 (-0.255--0.096) |
| Tajikistan | 22537.5 (18150.7-26952) | 17597.6 (14172.4-21044.6) | 37215.8 (30956.9-43891.3) | 18706.3 (15560.3-22061.7) | 0.348 (0.210-0.486) |
| Tanzania | 36814.1 (30245.7-44576.3) | 7556.5 (6208.3-9149.8) | 103241.8 (85368.3-123011.1) | 9295.3 (7686.1-11075.3) | 0.538 (0.429-0.648) |
| Thailand | 212650.7 (179290.3-243571.3) | 13911.6 (11729.2-15934.5) | 811749.5 (656256.1-968655.2) | 13029.4 (10533.6-15547.9) | -0.157 (-0.221--0.094) |
| The Bahamas | 614.1 (498.1-727.9) | 7596.6 (6161.3-9003.8) | 1911.8 (1604.5-2224) | 9864.2 (8278.6-11474.7) | 0.804 (0.709-0.900) |
| The Gambia | 1358.5 (1122-1628.5) | 8805.2 (7272.3-10554.9) | 4742.1 (3904.1-5663.7) | 10578.6 (8709.1-12634.4) | 0.613 (0.509-0.718) |
| Timor-Leste | 1339.4 (1114.1-1594.9) | 15697.8 (13057-18692.5) | 7110.2 (5920.3-8426.1) | 15723.6 (13092.2-18633.5) | -0.038 (-0.097-0.021) |
| Togo | 4297.4 (3480.6-5085.3) | 8476.8 (6865.7-10030.9) | 13930.1 (11430.6-16590.9) | 9638.3 (7908.9-11479.3) | 0.398 (0.319-0.477) |
| Tokelau | 11.1 (9.1-13.1) | 14693.4 (12086.1-17440.5) | 12.9 (10.3-15.2) | 14059.9 (11289-16644.4) | -0.063 (-0.156-0.029) |
| Tonga | 431 (356.1-503.4) | 16596.3 (13712.6-19382) | 656.6 (531.4-790) | 14986.5 (12128.8-18031.3) | -0.391 (-0.423--0.360) |
| Trinidad and Tobago | 4051.9 (3305.3-4794.3) | 8583.3 (7001.7-10155.9) | 11729.5 (9792.4-13866.8) | 10482.7 (8751.5-12392.8) | 0.616 (0.582-0.650) |
| Tunisia | 35768.6 (29752.7-42190.5) | 14795.5 (12307.1-17452) | 144429.1 (123028.5-166896.4) | 20743.8 (17670.1-23970.6) | 1.163 (1.118-1.208) |
| Turkey | 293510.9 (242583.3-351684) | 19567.1 (16172-23445.3) | 1204448.7 (1047207.1-1370875.8) | 23691.4 (20598.5-26965.1) | 0.748 (0.678-0.818) |
| Turkmenistan | 12254.1 (10078.2-14487.1) | 14473.6 (11903.6-17111.1) | 19998.2 (16762.6-23420) | 12766.5 (10700.9-14950.9) | -0.531 (-0.783--0.277) |
| Tuvalu | 40.9 (33.5-48.7) | 15156.5 (12408.4-18025.3) | 69.7 (56.3-83.2) | 13685.2 (11045.6-16332.8) | -0.321 (-0.382--0.261) |
| Uganda | 27927.9 (22667.5-33481.1) | 9542 (7744.7-11439.4) | 60798.8 (49992.5-72915) | 10104.3 (8308.3-12117.9) | 0.027 (-0.066-0.120) |
| Ukraine | 807219.8 (661711.2-965920.5) | 20138.2 (16508.1-24097.4) | 727380.8 (604316.7-865024.2) | 14915.6 (12392-17738.1) | -1.022 (-1.195--0.849) |
| United Arab Emirates | 1697.6 (1388.8-2014.5) | 13949.8 (11412-16554) | 7338.6 (6082.7-8726.6) | 15200.8 (12599.4-18076) | 0.146 (0.041-0.251) |
| United Kingdom | 1583543.2 (1374066.3-1783991) | 25448.5 (22082.1-28669.9) | 2610913.6 (2318031.1-2882524.7) | 28537.8 (25336.5-31506.5) | 0.487 (0.389-0.585) |
| United States | 5379060.1 (4738013-5992692) | 25362.3 (22339.7-28255.5) | 11405217.8 (10643073.6-12035490.3) | 29634.8 (27654.4-31272.4) | 0.703 (0.494-0.913) |
| Uruguay | 28390.7 (23409.5-33653.9) | 11566.1 (9536.8-13710.3) | 47422.2 (40472.4-55483.6) | 12620 (10770.6-14765.4) | 0.416 (0.311-0.521) |
| Uzbekistan | 90878.2 (74727.4-106541) | 15933.8 (13102.1-18680) | 140736 (116478.6-162326.7) | 14759.9 (12215.9-17024.2) | -0.225 (-0.398--0.052) |
| Vanuatu | 388 (319.8-455.4) | 16856.6 (13894-19785.2) | 1008.1 (819.4-1217.5) | 15142.5 (12308.5-18288.4) | -0.39 (-0.428--0.351) |
| Venezuela | 60742.8 (51654.9-71580.5) | 12795.6 (10881.2-15078.6) | 288545.3 (250404.8-339577) | 17967.4 (15592.5-21145.1) | 1.124 (0.979-1.270) |
| Vietnam | 285426.5 (234224.9-340362.8) | 13998.2 (11487.1-16692.5) | 784298 (657162.9-921683.8) | 17554.9 (14709.2-20629.9) | 0.733 (0.676-0.790) |
| Virgin Islands, U.S. | 325.7 (267.5-385.9) | 8128.5 (6677.5-9632.5) | 1432.3 (1185.9-1696.4) | 11106.5 (9196.2-13154.5) | 0.843 (0.764-0.921) |
| Yemen | 29382.4 (24261.9-35268.7) | 15061.5 (12436.7-18078.9) | 99836 (80777.7-119266.3) | 16783.4 (13579.5-20049.8) | 0.381 (0.302-0.459) |
| Zambia | 9133.6 (7458.8-10936.1) | 7885 (6439.1-9441) | 24529 (20070.2-28972.5) | 9115.7 (7458.7-10767.1) | 0.379 (0.280-0.479) |
| Zimbabwe | 18381.7 (15003.1-21962) | 10249.8 (8365.9-12246.2) | 29638.7 (24333.8-36116.9) | 11165.1 (9166.8-13605.6) | 0.259 (0.152-0.366) |

EAPC, estimated annual percentage change; UI, uncertainty interval; CI, confidence interval.

**S5 Table. Incidence and EAPC of chronic obstructive pulmonary disease among adults aged ≥70 years in 204 countries and territories, from 1990 to 2021**

|  | Incidence (95% UI) | | | |  |
| --- | --- | --- | --- | --- | --- |
|  | No in 1990 | Rate in 1990 (per 100,000) | No in 2021 | Rate in 2021 (per 100,000) | EAPCs (95% CI) |
| Afghanistan | 3146.5 (2439.7-3907.0) | 1012.5 (785.1-1257.2) | 5075 (3984.2-6282.7) | 1254.5 (984.9-1553.0) | 0.53 (0.45-0.62) |
| Albania | 1129.5 (888.4-1392.9) | 1131.5 (890.0-1395.3) | 3634.3 (2906.3-4389.3) | 1340.1 (1071.7-1618.5) | 0.66 (0.60-0.72) |
| Algeria | 6023.6 (4482.1-7739.3) | 967.2 (719.7-1242.7) | 23809.1 (19178.0-28729.8) | 1376.1 (1108.4-1660.5) | 1.1 (1.05-1.14) |
| American Samoa | 12.5 (10.2-14.8) | 1420.3 (1156.4-1691.2) | 27.2 (22.4-32.8) | 1229.3 (1009.7-1483.4) | -0.5 (-0.53--0.47) |
| Andorra | 47.1 (38.6-55.1) | 1470.2 (1204.3-1720.3) | 143.9 (122.9-168.5) | 1542 (1316.5-1806.0) | 0.26 (0.17-0.35) |
| Angola | 1115.8 (881.0-1359.9) | 870.1 (687.0-1060.4) | 3346.6 (2607.9-4095.6) | 834 (649.9-1020.6) | -0.28 (-0.36--0.19) |
| Antigua and Barbuda | 19.6 (15.8-23.9) | 538 (435.6-657.9) | 40.5 (31.3-51.6) | 721 (557.6-918.3) | 0.92 (0.84-0.99) |
| Argentina | 15652.3 (12352.0-19629.3) | 846.4 (667.9-1061.5) | 34081.1 (28049.4-39702.4) | 956.3 (787.1-1114.1) | 0.5 (0.34-0.66) |
| Armenia | 1564.6 (1248.9-1882.1) | 1309.7 (1045.4-1575.5) | 3685.7 (3077.0-4261.4) | 1507.6 (1258.6-1743.1) | 0.72 (0.54-0.89) |
| Australia | 12399.3 (9892.6-14703.7) | 1024.8 (817.6-1215.2) | 28188.4 (23153.7-34394.1) | 914 (750.8-1115.3) | -0.38 (-0.41--0.34) |
| Austria | 11104.6 (9160.8-13113.8) | 1456.5 (1201.6-1720.1) | 19495.7 (16633.8-22470.0) | 1567.3 (1337.2-1806.4) | 0.3 (0.25-0.35) |
| Azerbaijan | 2340.7 (1831.7-2882.7) | 1042 (815.4-1283.2) | 4920.3 (3933.2-5980.0) | 1284.1 (1026.4-1560.6) | 0.86 (0.67-1.06) |
| Bahamas | 41.3 (32.3-52.2) | 510.4 (400.1-645.8) | 131.9 (106.8-161.2) | 680.5 (551.1-831.5) | 0.86 (0.78-0.95) |
| Bahrain | 80.6 (61.9-98.1) | 1408.3 (1082.6-1715.0) | 372.6 (300.8-450.8) | 1479.1 (1193.9-1789.4) | 0.15 (0.05-0.24) |
| Bangladesh | 42383 (36836.0-47434.4) | 1862.7 (1618.9-2084.7) | 129682.5 (111112.9-145992.5) | 1759.7 (1507.7-1981.0) | -0.26 (-0.30--0.22) |
| Barbados | 114.6 (91.1-143.6) | 536.2 (426.1-672.1) | 233.1 (184.0-284.4) | 727.5 (574.1-887.4) | 1.01 (0.92-1.09) |
| Belarus | 8592.7 (6714.1-10201.0) | 1235.1 (965.1-1466.3) | 9007.9 (7128.9-11039.8) | 944.1 (747.1-1157.0) | -0.85 (-1.11--0.59) |
| Belgium | 13240.5 (10998.3-15602.0) | 1377.9 (1144.6-1623.7) | 23886.9 (19916.8-27692.0) | 1485.6 (1238.7-1722.3) | 0.41 (0.35-0.47) |
| Belize | 36.1 (29.1-44.4) | 685.4 (552.5-842.7) | 120.4 (97.6-145.3) | 862.6 (699.0-1040.5) | 0.92 (0.86-0.98) |
| Benin | 704.4 (569.3-838.4) | 686.7 (555.0-817.3) | 1560.9 (1253.1-1900.3) | 697.4 (559.9-849.1) | 0.05 (-0.01-0.11) |
| Bermuda | 22 (17.5-27.9) | 634.3 (503.8-803.2) | 80.3 (64.6-101.1) | 891.4 (717.1-1122.2) | 1.15 (1.05-1.24) |
| Bhutan | 173.2 (146.6-191.4) | 1931.4 (1635.4-2134.2) | 645.4 (571.5-705.8) | 2001.9 (1772.8-2189.2) | 0.17 (0.15-0.19) |
| Bolivia (Plurinational State of) | 1632.8 (1261.3-2016.0) | 1087.7 (840.3-1343.0) | 5978.2 (4808.7-7219.6) | 1291.9 (1039.2-1560.2) | 0.66 (0.62-0.70) |
| Bosnia and Herzegovina | 1889 (1461.0-2362.1) | 1126.7 (871.4-1408.9) | 5211 (4236.2-6296.6) | 1341 (1090.1-1620.4) | 0.76 (0.70-0.82) |
| Botswana | 218 (170.7-262.5) | 949 (743.3-1142.9) | 560.1 (453.8-688.5) | 956.6 (775.0-1175.9) | -0.04 (-0.17-0.09) |
| Brazil | 64096.3 (52430.1-74526.4) | 1508 (1233.6-1753.4) | 211919.1 (175694.3-246336.8) | 1507.1 (1249.5-1751.9) | -0.12 (-0.17--0.07) |
| Brunei Darussalam | 83.3 (72.8-91.7) | 1870.1 (1635.7-2059.2) | 194.3 (162.8-222.4) | 1424.3 (1193.4-1630.0) | -1 (-1.10--0.90) |
| Bulgaria | 7143.9 (5496.4-9103.3) | 1083.2 (833.4-1380.3) | 12099.6 (9629.4-14807.3) | 1175.4 (935.4-1438.4) | 0.37 (0.28-0.45) |
| Burkina Faso | 1090.3 (830.4-1342.4) | 555.4 (423.0-683.8) | 2629.3 (2095.6-3284.8) | 624.2 (497.5-779.9) | 0.35 (0.31-0.39) |
| Burundi | 964.1 (747.9-1201.3) | 852.1 (661.0-1061.7) | 1527.1 (1208.7-1852.7) | 847.7 (670.9-1028.4) | -0.1 (-0.14--0.05) |
| Cabo Verde | 58.5 (47.3-72.3) | 391.2 (316.3-483.2) | 96.4 (78.9-119.4) | 470.7 (385.3-582.9) | 0.64 (0.45-0.83) |
| Cambodia | 2001.7 (1562.1-2413.9) | 1073.4 (837.6-1294.4) | 6628.2 (5222.9-8099.1) | 1211.6 (954.7-1480.5) | 0.42 (0.37-0.47) |
| Cameroon | 1161.3 (924.1-1382.8) | 630.8 (501.9-751.1) | 3150.5 (2441.2-3916.9) | 629.7 (488.0-782.9) | -0.11 (-0.21--0.01) |
| Canada | 18751.5 (14631.6-23231.1) | 932.3 (727.5-1155.0) | 47255.4 (37436.7-57108.7) | 980.6 (776.9-1185.1) | -0.04 (-0.28-0.21) |
| Central African Republic | 356.4 (271.6-433.9) | 959.1 (731.0-1167.8) | 682.3 (539.5-829.8) | 1069.3 (845.6-1300.6) | 0.27 (0.22-0.31) |
| Chad | 984.3 (771.0-1197.1) | 653.5 (511.9-794.8) | 1751.5 (1387.5-2149.7) | 734.9 (582.2-902.0) | 0.3 (0.23-0.37) |
| Chile | 4375.7 (3536.9-5297.1) | 809.9 (654.6-980.4) | 14472.8 (12024.3-16850.1) | 929.1 (772.0-1081.8) | 0.53 (0.45-0.61) |
| China | 745840.9 (649118.0-815026.1) | 1987.3 (1729.6-2171.7) | 2117598.7 (1820377.8-2354084.6) | 1775 (1525.8-1973.2) | -0.43 (-0.48--0.38) |
| Colombia | 11326.8 (9462.7-13079.0) | 1354.1 (1131.2-1563.5) | 53680.2 (46638.4-60178.5) | 1684.5 (1463.5-1888.4) | 0.76 (0.73-0.80) |
| Comoros | 59.6 (46.9-72.2) | 735.2 (579.3-891.6) | 156.2 (127.1-189.9) | 688.3 (560.1-837.0) | -0.3 (-0.37--0.23) |
| Congo | 357.3 (284.4-433.6) | 819.4 (652.4-994.4) | 848.6 (662.6-1058.7) | 882.3 (688.9-1100.9) | 0.19 (0.13-0.25) |
| Cook Islands | 7.4 (6.0-9.0) | 1257 (1012.2-1525.7) | 17.9 (14.4-22.6) | 1147.5 (923.3-1451.0) | -0.39 (-0.50--0.29) |
| Costa Rica | 1254.1 (1048.1-1480.3) | 1324 (1106.5-1562.7) | 5109.5 (4322.9-5833.2) | 1629.3 (1378.5-1860.1) | 0.67 (0.60-0.74) |
| Croatia | 2638.3 (2057.1-3262.7) | 930.8 (725.7-1151.1) | 8628.3 (7139.5-10203.5) | 1403.4 (1161.2-1659.6) | 1.6 (1.52-1.68) |
| Cuba | 4755.1 (3797.9-5964.2) | 759.9 (606.9-953.1) | 13085.8 (10671.7-15853.5) | 1053.8 (859.4-1276.7) | 1.18 (1.07-1.29) |
| Cyprus | 753.9 (592.0-897.8) | 1461.9 (1147.9-1741.0) | 2143.5 (1787.4-2511.3) | 1583.3 (1320.3-1854.9) | 0.24 (0.16-0.32) |
| Czechia | 6379.1 (4899.1-7954.7) | 800.4 (614.7-998.1) | 18385 (14992.7-22161.3) | 1190.4 (970.8-1435.0) | 1.47 (1.39-1.55) |
| C么te d'Ivoire | 829.7 (652.1-1012.8) | 620.1 (487.3-757.0) | 2813.2 (2231.4-3508.8) | 674.7 (535.2-841.5) | 0.28 (0.21-0.36) |
| Democratic People's Republic of Korea | 13774.7 (12560.5-14752.9) | 2103.8 (1918.3-2253.2) | 35001 (30849.3-38436.0) | 2000 (1762.7-2196.2) | -0.28 (-0.32--0.24) |
| Democratic Republic of the Congo | 4871.6 (3812.6-5991.9) | 860.9 (673.7-1058.9) | 13186 (10656.5-16044.3) | 1042.8 (842.8-1268.9) | 0.62 (0.60-0.65) |
| Denmark | 7783.3 (6571.6-9158.7) | 1392.4 (1175.6-1638.4) | 13291.6 (11274.3-15492.4) | 1546.1 (1311.5-1802.1) | 0.28 (0.21-0.34) |
| Djibouti | 25.8 (20.6-31.3) | 607.7 (484.3-737.0) | 133.5 (104.9-165.8) | 609.7 (479.0-757.0) | -0.08 (-0.19-0.03) |
| Dominica | 24.0 (18.8-29.9) | 621.5 (487.5-774.6) | 35.8 (28.3-45.1) | 792.3 (627.7-999.1) | 0.73 (0.68-0.77) |
| Dominican Republic | 1194.1 (939.9-1509.8) | 647.6 (509.8-818.8) | 4999.7 (4050.2-6269.1) | 901 (729.9-1129.7) | 1.11 (1.07-1.15) |
| Ecuador | 2641.1 (2108.0-3210.2) | 975.3 (778.4-1185.4) | 12259.3 (10152.9-14645.8) | 1311.3 (1086.0-1566.6) | 1.07 (1.00-1.15) |
| Egypt | 7803.1 (6138.9-9822.8) | 793.9 (624.6-999.3) | 26728 (20946.7-32759.6) | 1177.3 (922.7-1443.0) | 1.23 (1.20-1.27) |
| El Salvador | 1694.4 (1386.1-2068.1) | 1069.4 (874.9-1305.3) | 5452.4 (4511.3-6477.3) | 1423.9 (1178.1-1691.5) | 0.98 (0.96-1.00) |
| Equatorial Guinea | 67.4 (53.4-82.1) | 898.6 (711.3-1093.6) | 176.8 (139.9-218.9) | 923.2 (730.7-1143.5) | 0.12 (-0.03-0.26) |
| Eritrea | 186.7 (146.0-228.7) | 662.8 (518.3-812.1) | 604.3 (480.2-742.2) | 658.3 (523.0-808.4) | -0.13 (-0.22--0.04) |
| Estonia | 655.1 (515.4-825.4) | 552.1 (434.4-695.7) | 1572.1 (1235.8-1907.4) | 821.6 (645.9-996.8) | 1.66 (1.51-1.82) |
| Eswatini | 112.5 (90.6-135.8) | 951.3 (766.5-1148.5) | 196.6 (154.5-239.6) | 874.3 (687.3-1065.7) | -0.46 (-0.57--0.36) |
| Ethiopia | 5895.2 (4417.2-7513.6) | 762.8 (571.5-972.2) | 14150 (11188.3-17145.4) | 717.7 (567.5-869.6) | -0.3 (-0.39--0.20) |
| Fiji | 171.0 (135.6-205.0) | 1264.7 (1003.0-1515.6) | 308.9 (245.8-383.0) | 960.1 (763.9-1190.4) | -1.02 (-1.09--0.94) |
| Finland | 4235.8 (3424.2-5173.5) | 926.2 (748.7-1131.2) | 11281 (9271.8-13913.5) | 1204.1 (989.7-1485.1) | 0.89 (0.81-0.97) |
| France | 70177.6 (57673.7-82484.3) | 1348.7 (1108.4-1585.2) | 133444.1 (111903.7-156746.5) | 1343.5 (1126.6-1578.1) | 0.2 (0.08-0.33) |
| Gabon | 210.8 (171.7-253.4) | 746.9 (608.5-898.0) | 327.3 (258.4-404.7) | 807.9 (637.8-998.8) | 0.27 (0.16-0.38) |
| Gambia | 97.6 (75.6-119.6) | 632.8 (490.2-775.0) | 326.3 (254.9-405.1) | 727.9 (568.7-903.8) | 0.44 (0.35-0.54) |
| Georgia | 2712.7 (2124.6-3403.9) | 821.7 (643.6-1031.1) | 4037.9 (3157.9-4958.9) | 1082.5 (846.6-1329.4) | 1.04 (0.84-1.25) |
| Germany | 125032.8 (104397.6-147096.7) | 1546.6 (1291.4-1819.5) | 226012.1 (189612.4-256867.0) | 1667.3 (1398.8-1894.9) | 0.32 (0.16-0.49) |
| Ghana | 1521.3 (1198.7-1858.7) | 600.4 (473.0-733.5) | 4950.5 (3872.7-6133.8) | 719.1 (562.6-891.0) | 0.62 (0.56-0.68) |
| Greece | 13433.3 (10848.9-15922.2) | 1418 (1145.2-1680.8) | 28988.8 (24470.8-33024.0) | 1674.4 (1413.5-1907.5) | 0.62 (0.55-0.70) |
| Greenland | 16.9 (13.4-20.1) | 1360.4 (1083.1-1617.1) | 37.5 (29.6-45.4) | 1245.8 (982.6-1508.1) | -0.37 (-0.45--0.29) |
| Grenada | 30.2 (24.0-37.0) | 606.4 (482.6-743.8) | 43.6 (34.2-54.2) | 738.4 (578.0-916.7) | 0.41 (0.28-0.54) |
| Guam | 30.4 (23.7-38.4) | 1090.7 (852.4-1378.3) | 127 (106.1-154.0) | 1038.1 (866.8-1258.6) | -0.07 (-0.10--0.03) |
| Guatemala | 1626.2 (1298.8-1974.5) | 1108.3 (885.2-1345.7) | 8286.7 (6697.5-9529.6) | 1405.8 (1136.2-1616.6) | 0.91 (0.86-0.97) |
| Guinea | 1092.2 (863.6-1336.9) | 658.7 (520.8-806.2) | 1945.8 (1551.7-2385.4) | 749.8 (597.9-919.2) | 0.57 (0.49-0.65) |
| Guinea-Bissau | 105.2 (84.5-129.4) | 642.9 (516.4-790.9) | 164 (129.4-201.5) | 646 (509.9-793.9) | -0.02 (-0.13-0.08) |
| Guyana | 94 (73.0-116.0) | 535.9 (415.8-661.3) | 192 (151.1-238.8) | 663.6 (522.2-825.3) | 0.71 (0.68-0.73) |
| Haiti | 1087.5 (829.8-1371.6) | 822.3 (627.5-1037.2) | 2730.9 (2159.2-3342.7) | 960.9 (759.7-1176.1) | 0.49 (0.43-0.54) |
| Honduras | 1286 (1028.6-1547.8) | 1318 (1054.2-1586.3) | 5192.8 (4244.8-6044.0) | 1647.8 (1347.0-1917.9) | 0.7 (0.63-0.76) |
| Hungary | 9084.7 (7030.2-11283.3) | 1071.6 (829.2-1330.9) | 17824.7 (15177.1-21377.2) | 1323.5 (1126.9-1587.3) | 0.81 (0.77-0.85) |
| Iceland | 281.6 (232.4-331.4) | 1541.4 (1272.1-1813.5) | 584.9 (493.9-674.3) | 1545.9 (1305.6-1782.2) | 0 (-0.11-0.11) |
| India | 359315.9 (319337.9-388973.1) | 1994.9 (1772.9-2159.5) | 1146072 (1016780.1-1245560.7) | 1923.5 (1706.5-2090.4) | -0.14 (-0.15--0.13) |
| Indonesia | 41374.3 (33329.2-49361.9) | 1086.5 (875.2-1296.2) | 125825.7 (100612.1-150543.9) | 1304.4 (1043.1-1560.7) | 0.58 (0.56-0.60) |
| Iran (Islamic Republic of) | 7274.5 (5795.8-8970.2) | 749.7 (597.3-924.5) | 45807.9 (37403.6-55112.6) | 1254.4 (1024.3-1509.2) | 2.02 (1.93-2.11) |
| Iraq | 2825.4 (2255.1-3513.0) | 707.3 (564.5-879.4) | 9571.6 (7502.8-12017.3) | 965.1 (756.5-1211.6) | 1.11 (1.00-1.21) |
| Ireland | 4165.7 (3491.9-4845.8) | 1550.3 (1299.5-1803.4) | 8308.5 (7112.0-9520.4) | 1598.2 (1368.1-1831.4) | 0.14 (0.09-0.18) |
| Israel | 4158.3 (3358.1-4976.3) | 1367.8 (1104.6-1636.8) | 10959.1 (8742.1-12957.4) | 1327.4 (1058.8-1569.4) | 0.07 (-0.00-0.15) |
| Italy | 78431.9 (64060.2-92972.6) | 1416 (1156.5-1678.5) | 170081.6 (144832.2-194603.3) | 1617.3 (1377.2-1850.4) | 0.52 (0.46-0.58) |
| Jamaica | 777.1 (625.6-970.6) | 668 (537.7-834.4) | 1602.8 (1313.2-1942.2) | 881.2 (722.0-1067.8) | 0.99 (0.90-1.09) |
| Japan | 108467 (86237.0-132203.4) | 1098.2 (873.1-1338.5) | 333191.7 (274402.4-405022.2) | 1152.8 (949.4-1401.4) | 0.21 (0.16-0.27) |
| Jordan | 433 (341.2-531.1) | 882.8 (695.7-1082.9) | 3282.8 (2618.0-4194.3) | 1069 (852.5-1365.8) | 0.61 (0.54-0.67) |
| Kazakhstan | 6967.6 (5576.5-8531.3) | 1161.3 (929.5-1421.9) | 12227.3 (10110.7-14602.7) | 1503.4 (1243.2-1795.5) | 1.22 (0.96-1.47) |
| Kenya | 2506.8 (1958.3-3009.8) | 676.5 (528.5-812.3) | 6784.6 (5315.8-8219.7) | 727.1 (569.7-880.9) | 0.19 (0.10-0.29) |
| Kiribati | 20.7 (16.7-24.8) | 1435.8 (1156.9-1718.0) | 33.6 (27.2-40.4) | 1352.3 (1093.8-1622.3) | -0.18 (-0.22--0.13) |
| Kuwait | 146.6 (117.4-181.3) | 734.2 (588.2-907.8) | 1152.6 (943.6-1378.7) | 1167.4 (955.8-1396.4) | 1.7 (1.58-1.83) |
| Kyrgyzstan | 2386.1 (1997.2-2770.6) | 1650 (1381.1-1915.9) | 2834.5 (2306.7-3361.9) | 1476.8 (1201.8-1751.5) | -0.38 (-0.47--0.29) |
| Lao People's Democratic Republic | 1238 (1006.0-1459.9) | 1395.1 (1133.7-1645.1) | 2641.8 (2134.5-3132.7) | 1371.4 (1108.1-1626.3) | -0.13 (-0.18--0.07) |
| Latvia | 1476.8 (1138.6-1862.5) | 711.8 (548.8-897.7) | 2447.1 (1932.0-3050.5) | 869 (686.1-1083.3) | 0.79 (0.52-1.06) |
| Lebanon | 818.6 (638.1-1027.5) | 855.9 (667.2-1074.4) | 5245.9 (4172.4-6264.0) | 1364.2 (1085.1-1629.0) | 1.69 (1.61-1.77) |
| Lesotho | 458.5 (369.4-554.1) | 1069 (861.1-1291.7) | 541.3 (418.7-662.2) | 1125.9 (870.8-1377.3) | 0.06 (-0.03-0.15) |
| Liberia | 426.5 (336.6-522.1) | 716.9 (565.7-877.5) | 706.6 (569.5-868.8) | 886 (714.1-1089.4) | 0.6 (0.50-0.69) |
| Libya | 669.1 (531.6-824.9) | 769.9 (611.6-949.2) | 2441.6 (1960.4-3070.0) | 1114.1 (894.6-1400.9) | 1.31 (1.25-1.37) |
| Lithuania | 2265.3 (1761.3-2809.9) | 892.5 (693.9-1107.1) | 3681.7 (3011.6-4448.9) | 916.7 (749.8-1107.7) | 0.16 (-0.07-0.38) |
| Luxembourg | 488 (409.7-583.4) | 1429 (1199.9-1708.3) | 1022.2 (860.7-1174.8) | 1538 (1295.0-1767.7) | 0.32 (0.28-0.36) |
| Madagascar | 1799.1 (1406.0-2209.4) | 838.1 (654.9-1029.2) | 3361.9 (2628.8-4066.3) | 924.3 (722.7-1117.9) | 0.33 (0.28-0.38) |
| Malawi | 907.2 (712.3-1107.3) | 583.3 (458.0-711.9) | 1921.3 (1509.7-2369.5) | 617.6 (485.3-761.7) | 0.14 (0.10-0.18) |
| Malaysia | 5053.1 (4170.6-5988.2) | 1161.2 (958.4-1376.1) | 14316.2 (11666.4-17533.6) | 1012.4 (825.0-1239.9) | -0.59 (-0.67--0.50) |
| Maldives | 42.5 (33.6-51.9) | 1415.4 (1121.6-1729.9) | 207.5 (177.0-241.6) | 1525.8 (1301.5-1776.4) | 0.2 (0.06-0.35) |
| Mali | 774.4 (594.4-978.7) | 476.6 (365.8-602.3) | 2085.5 (1607.3-2653.1) | 564.9 (435.4-718.6) | 0.51 (0.38-0.63) |
| Malta | 301.2 (249.6-355.9) | 1213.1 (1005.2-1433.6) | 892.3 (717.6-1054.8) | 1242.9 (999.6-1469.2) | 0.11 (0.05-0.17) |
| Marshall Islands | 11.4 (9.1-13.6) | 1556.3 (1241.1-1853.2) | 13.8 (11.0-16.5) | 1204.5 (960.0-1441.2) | -0.93 (-1.01--0.85) |
| Mauritania | 268.2 (214.0-326.6) | 514 (410.1-626.0) | 650.1 (514.6-814.5) | 638.6 (505.5-800.1) | 0.69 (0.62-0.75) |
| Mauritius | 289.2 (232.6-347.9) | 878.1 (706.3-1056.4) | 971.3 (795.8-1166.8) | 968.6 (793.6-1163.6) | 0.29 (0.27-0.31) |
| Mexico | 29200.6 (24029.8-34220.2) | 1406 (1157.1-1647.7) | 110798.5 (93822.4-126530.7) | 1639.8 (1388.6-1872.7) | 0.52 (0.45-0.58) |
| Micronesia (Federated States of) | 39.9 (33.2-46.9) | 1698.3 (1412.0-1998.0) | 33.9 (27.3-41.2) | 1340.5 (1079.9-1629.5) | -0.81 (-0.84--0.78) |
| Monaco | 67.6 (53.9-80.6) | 1364.9 (1088.5-1626.1) | 99.4 (81.9-119.7) | 1396.8 (1150.8-1681.0) | 0.18 (0.05-0.31) |
| Mongolia | 581.3 (460.8-711.8) | 1131.2 (896.7-1385.2) | 998.2 (767.6-1221.6) | 1186.5 (912.3-1451.9) | 0.17 (0.06-0.28) |
| Montenegro | 248.9 (194.9-308.7) | 777.7 (609.1-964.7) | 599.2 (473.6-748.9) | 1030 (814.1-1287.3) | 1.16 (1.08-1.24) |
| Morocco | 5520 (4377.0-6693.3) | 778.2 (617.0-943.6) | 19442.8 (15416.2-24315.8) | 1176.5 (932.9-1471.4) | 1.34 (1.25-1.43) |
| Mozambique | 1490.7 (1174.6-1796.1) | 613.4 (483.3-739.0) | 2731.5 (2109.7-3362.1) | 635.4 (490.8-782.1) | 0.01 (-0.04-0.06) |
| Myanmar | 17028.4 (14107.3-19600.4) | 1741 (1442.4-2004.0) | 40358.8 (35119.9-44980.2) | 1806 (1571.6-2012.8) | 0.21 (0.17-0.25) |
| Namibia | 256.3 (202.2-310.8) | 965.2 (761.4-1170.4) | 596.9 (475.9-736.9) | 997.1 (794.9-1230.9) | 0.06 (-0.02-0.13) |
| Nauru | 2.4 (1.9-3.0) | 1548.8 (1229.9-1873.8) | 2.9 (2.4-3.5) | 1304.4 (1047.2-1552.4) | -0.61 (-0.68--0.55) |
| Nepal | 8022 (7433.0-8465.1) | 2200.1 (2038.5-2321.6) | 26128.5 (24934.8-27173.7) | 2242.7 (2140.3-2332.5) | 0.1 (0.08-0.11) |
| Netherlands | 14122.3 (11736.4-16566.2) | 1100.9 (914.9-1291.4) | 32246.6 (26776.8-37830.7) | 1313 (1090.3-1540.4) | 0.87 (0.66-1.07) |
| New Zealand | 2694.9 (2146.0-3231.6) | 1089.2 (867.3-1306.1) | 5855 (4642.7-7266.9) | 1046.5 (829.8-1298.9) | 0.01 (-0.12-0.14) |
| Nicaragua | 870.6 (699.3-1086.8) | 1182.9 (950.1-1476.7) | 4239.7 (3588.2-4834.7) | 1698.1 (1437.1-1936.3) | 1.26 (1.13-1.39) |
| Niger | 661.8 (512.6-819.5) | 643.8 (498.6-797.1) | 2548.8 (1918.3-3159.8) | 748 (563.0-927.4) | 0.53 (0.46-0.59) |
| Nigeria | 11996.1 (9455.5-14556.8) | 572.6 (451.3-694.8) | 27445.9 (22020.0-33482.5) | 733 (588.1-894.2) | 0.9 (0.84-0.97) |
| Niue | 2.2 (1.8-2.6) | 1475.5 (1232.2-1746.1) | 1.4 (1.2-1.8) | 1222.1 (998.4-1509.3) | -0.69 (-0.78--0.60) |
| North Macedonia | 961 (754.4-1188.4) | 1090.4 (856.1-1348.4) | 2633.8 (2085.2-3255.6) | 1386.3 (1097.5-1713.5) | 0.9 (0.81-0.99) |
| Northern Mariana Islands | 5.5 (4.4-6.9) | 1143.3 (906.4-1439.6) | 22.9 (18.2-28.9) | 1081.7 (860.8-1364.3) | -0.07 (-0.17-0.03) |
| Norway | 6087 (4917.6-7355.6) | 1245.8 (1006.5-1505.5) | 11233.6 (9457.4-12926.8) | 1622.7 (1366.1-1867.3) | 0.96 (0.77-1.15) |
| Oman | 252.4 (195.9-318.8) | 874.7 (679.0-1104.8) | 864.5 (664.5-1078.5) | 1252 (962.3-1562.0) | 1.09 (1.04-1.14) |
| Pakistan | 52739.4 (44527.5-59977.4) | 1865.5 (1575.1-2121.6) | 91954.2 (77021.7-104162.5) | 1815.9 (1521.1-2057.0) | -0.13 (-0.17--0.08) |
| Palau | 6.7 (5.5-8.0) | 1409.4 (1141.9-1675.8) | 10.4 (8.3-12.6) | 1138.6 (905.0-1386.0) | -0.72 (-0.82--0.63) |
| Palestine | 373.4 (290.9-467.3) | 885.3 (689.8-1108.0) | 1143.2 (913.9-1430.4) | 1101.2 (880.4-1377.9) | 0.8 (0.69-0.92) |
| Panama | 858.0 (687.0-1062.4) | 1063.2 (851.4-1316.6) | 3712.1 (3166.1-4283.1) | 1422.4 (1213.2-1641.2) | 0.84 (0.71-0.96) |
| Papua New Guinea | 1201.6 (1052.7-1308.9) | 2001.3 (1753.3-2180.0) | 3588.1 (3216.7-3884.0) | 2061.8 (1848.4-2231.8) | 0.14 (0.08-0.20) |
| Paraguay | 1231.1 (972.6-1518.4) | 1061.7 (838.8-1309.4) | 3976.7 (3282.9-4772.5) | 1317.3 (1087.5-1580.9) | 0.76 (0.73-0.79) |
| Peru | 4570.2 (3587.0-5638.8) | 762.3 (598.3-940.5) | 18994.7 (15167.1-23792.2) | 1008.9 (805.6-1263.7) | 1.01 (0.93-1.08) |
| Philippines | 18921.5 (15526.6-22011.7) | 1460 (1198.1-1698.5) | 42159.7 (34195.7-50638.7) | 1165.5 (945.3-1399.8) | -1.01 (-1.14--0.88) |
| Poland | 21802.9 (17234.6-26911.0) | 906.1 (716.2-1118.3) | 50976.2 (41154.0-61387.3) | 1091.4 (881.1-1314.3) | 0.63 (0.54-0.72) |
| Portugal | 11822 (9884.8-13558.1) | 1375.3 (1149.9-1577.2) | 28088.2 (24341.8-32200.4) | 1587.3 (1375.6-1819.7) | 0.53 (0.46-0.59) |
| Puerto Rico | 1914.1 (1544.9-2323.8) | 856.4 (691.2-1039.7) | 5838.3 (4831.8-7018.9) | 1108.5 (917.4-1332.6) | 0.72 (0.61-0.82) |
| Qatar | 23.5 (18.5-28.8) | 974.4 (766.2-1192.2) | 244.2 (196.9-288.3) | 1390.5 (1121.1-1641.4) | 1.31 (1.24-1.38) |
| Republic of Korea | 16907.5 (13163.5-20062.0) | 1332 (1037.0-1580.5) | 71350 (59920.4-82895.6) | 1287.9 (1081.6-1496.3) | -0.1 (-0.19--0.01) |
| Republic of Moldova | 2200.9 (1697.7-2729.9) | 1047.8 (808.3-1299.7) | 3324.9 (2645.2-4181.6) | 954.1 (759.1-1199.9) | -0.38 (-0.53--0.23) |
| Romania | 17693.7 (13742.6-21588.0) | 1240 (963.1-1512.9) | 29649.3 (24314.3-35462.5) | 1171.2 (960.5-1400.9) | -0.18 (-0.28--0.07) |
| Russian Federation | 100557.1 (79566.5-123671.9) | 1041.4 (824.0-1280.8) | 129298.2 (103904.1-158363.0) | 903.7 (726.2-1106.9) | -0.52 (-0.73--0.31) |
| Rwanda | 947.7 (725.4-1186.1) | 858.2 (656.9-1074.2) | 1995.4 (1580.1-2481.7) | 807.6 (639.5-1004.4) | -0.29 (-0.37--0.21) |
| Saint Kitts and Nevis | 14.7 (11.6-18.3) | 544.1 (427.5-675.4) | 20.9 (16.8-25.7) | 753.1 (603.5-923.7) | 1.16 (0.96-1.35) |
| Saint Lucia | 41.0 (32.0-51.2) | 784 (612.7-978.9) | 130.1 (107.6-155.8) | 957.5 (791.9-1146.8) | 0.67 (0.57-0.76) |
| Saint Vincent and the Grenadines | 22.8 (17.4-28.9) | 523.5 (398.5-663.4) | 56.4 (44.8-68.8) | 708.5 (562.6-865.2) | 1.05 (1.02-1.09) |
| Samoa | 64.0 (53.8-74.3) | 1688.3 (1418.6-1959.5) | 97 (78.8-116.2) | 1420.4 (1153.7-1700.7) | -0.57 (-0.60--0.54) |
| San Marino | 30.2 (24.7-36.1) | 1291.3 (1056.2-1547.6) | 72.3 (59.6-85.1) | 1387.8 (1144.5-1632.9) | 0.27 (0.20-0.34) |
| Sao Tome and Principe | 29 (23.5-35.1) | 878.7 (712.6-1061.9) | 48.2 (39.2-57.6) | 1050.9 (855.9-1257.3) | 0.61 (0.54-0.68) |
| Saudi Arabia | 1869.1 (1500.6-2305.9) | 752.9 (604.5-928.9) | 5170 (3988.7-6502.6) | 996.2 (768.6-1253.0) | 1.02 (0.94-1.10) |
| Senegal | 988.6 (778.3-1195.4) | 653.8 (514.7-790.5) | 2515.3 (2003.8-3116.4) | 710.4 (565.9-880.2) | 0.32 (0.26-0.39) |
| Serbia | 4795.6 (3825.1-5961.0) | 965.6 (770.2-1200.2) | 14746 (11953.1-17961.0) | 1363.9 (1105.6-1661.2) | 1.25 (1.18-1.32) |
| Seychelles | 30 (23.9-36.8) | 875.9 (699.3-1075.2) | 58.4 (47.1-71.8) | 1057.5 (852.1-1299.5) | 0.5 (0.45-0.55) |
| Sierra Leone | 699.7 (557.2-849.9) | 647.7 (515.8-786.7) | 1196.5 (937.6-1486.8) | 697.8 (546.8-867.1) | 0.12 (0.07-0.17) |
| Singapore | 991.5 (786.3-1193.6) | 947.3 (751.2-1140.4) | 3345 (2658.3-4073.3) | 692.3 (550.2-843.0) | -1.11 (-1.24--0.98) |
| Slovakia | 2556.8 (1952.4-3221.4) | 773.4 (590.6-974.5) | 6157.5 (4919.7-7648.5) | 1024.2 (818.3-1272.2) | 1.08 (1.01-1.15) |
| Slovenia | 1337.4 (1069.2-1646.2) | 976.6 (780.8-1202.1) | 3624.4 (2949.4-4359.7) | 1206.2 (981.6-1450.9) | 0.85 (0.80-0.91) |
| Solomon Islands | 82.2 (67.3-94.3) | 1751 (1432.6-2008.7) | 212.7 (178.2-249.5) | 1604.6 (1344.9-1882.7) | -0.31 (-0.33--0.29) |
| Somalia | 621 (487.6-761.0) | 884.8 (694.8-1084.4) | 1640.7 (1251.2-2017.0) | 861.2 (656.7-1058.7) | -0.16 (-0.27--0.05) |
| South Africa | 11640.3 (9537.9-13882.0) | 1147.3 (940.1-1368.3) | 24887.4 (20314.4-29779.7) | 1124.9 (918.2-1346.0) | -0.11 (-0.17--0.04) |
| South Sudan | 887.1 (696.0-1088.1) | 705.9 (553.9-865.9) | 992.1 (788.0-1216.3) | 712.7 (566.1-873.7) | -0.15 (-0.25--0.06) |
| Spain | 54344.2 (46043.0-62831.0) | 1576.7 (1335.9-1823.0) | 114711.1 (101273.4-130057.0) | 1695.2 (1496.6-1922.0) | 0.32 (0.28-0.36) |
| Sri Lanka | 4574.3 (3609.4-5581.1) | 927.6 (731.9-1131.7) | 16742.8 (13281.2-20885.0) | 1100.4 (872.9-1372.7) | 0.46 (0.39-0.52) |
| Sudan | 4715.8 (3596.9-5951.5) | 1000.6 (763.2-1262.8) | 10383.3 (8217.9-12677.0) | 1212.7 (959.8-1480.6) | 0.49 (0.40-0.57) |
| Suriname | 78.9 (61.8-97.4) | 645.8 (505.8-797.1) | 258.2 (206.6-328.1) | 776.1 (621.1-986.2) | 0.58 (0.56-0.61) |
| Sweden | 16373.2 (13543.7-18952.0) | 1500 (1240.8-1736.3) | 27383.4 (23375.5-30828.5) | 1717 (1465.7-1933.0) | 0.48 (0.37-0.58) |
| Switzerland | 9650.1 (7903.0-11458.3) | 1383.5 (1133.0-1642.7) | 18527.3 (15574.5-21335.6) | 1485 (1248.3-1710.1) | 0.21 (0.14-0.28) |
| Syrian Arab Republic | 1945.8 (1544.2-2371.5) | 873.4 (693.1-1064.5) | 7295 (5756.7-8772.8) | 1235.4 (974.9-1485.6) | 1.31 (1.25-1.38) |
| Taiwan (Province of China) | 8863 (7174.1-10534.7) | 1215.3 (983.7-1444.5) | 29786.3 (25499.6-34223.0) | 1179.4 (1009.7-1355.1) | 0.01 (-0.06-0.08) |
| Tajikistan | 1393.6 (1086.9-1744.3) | 1088.1 (848.7-1362.0) | 2698.3 (2165.7-3309.9) | 1356.3 (1088.6-1663.7) | 0.93 (0.82-1.04) |
| Thailand | 17160.7 (14154.3-20127.3) | 1122.7 (926.0-1316.7) | 62292.3 (50403.3-75790.3) | 999.9 (809.0-1216.5) | -0.42 (-0.52--0.31) |
| Timor-Leste | 113.2 (92.9-134.1) | 1327.2 (1089.1-1571.1) | 605.7 (490.9-729.6) | 1339.4 (1085.5-1613.4) | 0.04 (-0.02-0.10) |
| Togo | 316.4 (248.8-385.3) | 624.2 (490.7-759.9) | 912.3 (696.1-1124.5) | 631.2 (481.6-778.0) | -0.06 (-0.12--0.00) |
| Tokelau | 1.1 (0.9-1.3) | 1447.5 (1202.2-1685.8) | 1.1 (0.9-1.4) | 1228.9 (1016.7-1479.7) | -0.52 (-0.61--0.43) |
| Tonga | 39 (32.3-45.3) | 1503.2 (1242.2-1744.2) | 57.1 (47.7-68.7) | 1302.9 (1088.0-1569.2) | -0.52 (-0.57--0.48) |
| Trinidad and Tobago | 278.1 (218.9-344.3) | 589.2 (463.7-729.3) | 791 (619.2-1010.6) | 706.9 (553.4-903.2) | 0.54 (0.53-0.55) |
| Tunisia | 2447.5 (1861.4-3042.4) | 1012.4 (770.0-1258.5) | 9441.7 (7635.2-11226.4) | 1356.1 (1096.6-1612.4) | 0.93 (0.89-0.96) |
| Turkey | 19621.6 (15563.0-23862.6) | 1308.1 (1037.5-1590.8) | 81873.1 (68627.3-94045.7) | 1610.4 (1349.9-1849.9) | 0.81 (0.74-0.87) |
| Turkmenistan | 816.2 (642.7-1010.8) | 964.1 (759.1-1193.9) | 1280.8 (1021.4-1561.7) | 817.7 (652.1-996.9) | -0.63 (-0.92--0.35) |
| Tuvalu | 4.3 (3.5-5.1) | 1585 (1282.0-1899.0) | 6.5 (5.3-7.8) | 1274.2 (1034.6-1535.3) | -0.77 (-0.84--0.70) |
| Uganda | 2393.9 (1883.5-2923.5) | 817.9 (643.5-998.9) | 4544.5 (3647.7-5578.9) | 755.3 (606.2-927.2) | -0.41 (-0.48--0.33) |
| Ukraine | 56020.6 (44843.2-67722.0) | 1397.6 (1118.7-1689.5) | 45886.3 (36069.6-56669.7) | 940.9 (739.6-1162.1) | -1.41 (-1.62--1.20) |
| United Arab Emirates | 123.4 (96.1-152.7) | 1014.1 (789.9-1254.8) | 510.2 (386.2-662.3) | 1056.8 (800.0-1371.9) | -0.07 (-0.19-0.06) |
| United Kingdom | 101507 (86017.3-115496.7) | 1631.3 (1382.4-1856.1) | 164792.4 (144332.5-181661.3) | 1801.2 (1577.6-1985.6) | 0.36 (0.30-0.43) |
| United Republic of Tanzania | 2743.7 (2179.9-3316.8) | 563.2 (447.4-680.8) | 6910.6 (5387.7-8463.8) | 622.2 (485.1-762.0) | 0.21 (0.12-0.31) |
| United States of America | 242380.2 (194920.5-285058.1) | 1142.8 (919.0-1344.0) | 542332.4 (474844.4-602845.3) | 1409.2 (1233.8-1566.4) | 0.73 (0.53-0.93) |
| United States Virgin Islands | 22.4 (17.5-28.5) | 559.5 (437.9-710.8) | 98.9 (76.7-123.4) | 767 (594.8-956.5) | 0.9 (0.85-0.95) |
| Uruguay | 2010.4 (1598.9-2482.9) | 819 (651.4-1011.5) | 3700.6 (3065.2-4358.3) | 984.8 (815.7-1159.8) | 0.75 (0.61-0.88) |
| Uzbekistan | 5811.6 (4702.7-7046.9) | 1019 (824.5-1235.5) | 9519.4 (7639.8-11919.8) | 998.4 (801.2-1250.1) | -0.01 (-0.17-0.15) |
| Vanuatu | 40.6 (33.0-47.4) | 1762.5 (1434.7-2060.2) | 101.2 (84.1-118.0) | 1520 (1263.5-1772.9) | -0.59 (-0.65--0.52) |
| Venezuela (Bolivarian Republic of) | 4651.8 (3743.8-5800.4) | 979.9 (788.6-1221.9) | 22109.9 (18809.0-26156.2) | 1376.8 (1171.2-1628.7) | 1.12 (0.99-1.25) |
| Viet Nam | 24529 (19861.7-29443.6) | 1203 (974.1-1444.0) | 62273.2 (51248.0-73104.8) | 1393.9 (1147.1-1636.3) | 0.49 (0.43-0.55) |
| Yemen | 2069 (1623.0-2579.8) | 1060.6 (831.9-1322.4) | 7291.7 (5648.7-8999.9) | 1225.8 (949.6-1513.0) | 0.5 (0.41-0.59) |
| Zambia | 780.2 (616.7-932.2) | 673.5 (532.4-804.7) | 1786.4 (1405.7-2205.3) | 663.9 (522.4-819.6) | -0.13 (-0.19--0.07) |
| Zimbabwe | 1287.2 (1002.9-1575.3) | 717.7 (559.2-878.4) | 2026.3 (1537.8-2569.8) | 763.3 (579.3-968.1) | 0.14 (0.03-0.25) |

EAPC, estimated annual percentage change; UI, uncertainty interval; CI, confidence interval.

**S6 Table. Death and EAPC of chronic obstructive pulmonary disease among adults aged ≥70 years in 204 countries and territories, from 1990 to 2021**

|  | **Prevalence (95% UI)** | | | |  |
| --- | --- | --- | --- | --- | --- |
|  | **No in 1990** | **Rate in 1990 (per 100,000)** | **No in 2021** | **Rate in 2021 (per 100,000)** | **EAPCs (95% CI)** |
| Afghanistan | 43669.8 (35905.1-51660.7) | 14052 (11553.5-16623.3) | 69404.9 (57616.9-83316.1) | 17156.4 (14242.5-20595.1) | 0.525 (0.466-0.584) |
| Albania | 17339 (14482.9-20436.4) | 17369.5 (14508.4-20472.3) | 54871.6 (46003.9-63480.4) | 20233.6 (16963.7-23408.1) | 0.614 (0.536-0.693) |
| Algeria | 81646.2 (68388.9-98216) | 13109.7 (10981-15770.3) | 336152.6 (281666.5-390445.8) | 19428.7 (16279.5-22566.7) | 1.259 (1.205-1.314) |
| American Samoa | 133.5 (109.9-157.3) | 15203.6 (12517.4-17914.6) | 307.2 (249.2-367.2) | 13874.4 (11253.1-16582.2) | -0.29 (-0.323--0.257) |
| Andorra | 680.5 (575.1-781) | 21246.1 (17955.4-24383.2) | 2107.7 (1820.9-2412.1) | 22584.2 (19511.5-25845.4) | 0.293 (0.195-0.391) |
| Angola | 13319.4 (10851.2-15841.4) | 10385.8 (8461.2-12352.3) | 43644.1 (35745-51227.7) | 10876 (8907.6-12765.8) | 0.052 (-0.029-0.133) |
| Antigua and Barbuda | 279.8 (227.4-330) | 7690.8 (6249.6-9072.5) | 569.5 (462.4-682.5) | 10139.9 (8231.8-12151.4) | 0.888 (0.799-0.977) |
| Argentina | 217700.8 (176584.4-265558.6) | 11772.4 (9549-14360.3) | 454742.4 (387367.5-527335.7) | 12760.5 (10869.9-14797.5) | 0.414 (0.259-0.569) |
| Armenia | 22574.4 (19038-26268.7) | 18896.7 (15936.4-21989.1) | 51269.3 (42733.8-60471.4) | 20971.4 (17480-24735.5) | 0.634 (0.358-0.912) |
| Australia | 208085.6 (173854.1-234806) | 17198 (14368.8-19406.4) | 435536.2 (372380.7-509289) | 14122.8 (12074.9-16514.3) | -0.586 (-0.705--0.467) |
| Austria | 172072.3 (151871.7-193707.2) | 22570 (19920.4-25407.7) | 307219.8 (269375.3-343724.2) | 24697.5 (21655.2-27632.1) | 0.345 (0.260-0.429) |
| Azerbaijan | 35569.5 (29227.5-41907.1) | 15833.9 (13010.7-18655.1) | 69583 (57121.7-82639.3) | 18159.1 (14907.1-21566.5) | 0.505 (0.277-0.733) |
| Bahrain | 944.6 (775.9-1116.4) | 16510.9 (13562.8-19513.5) | 4646.9 (3891.7-5416) | 18445.4 (15447.6-21498.4) | 0.409 (0.308-0.510) |
| Bangladesh | 471321.8 (408052.3-541768.5) | 20714.6 (17933.9-23810.7) | 1681887.5 (1460307.7-1900459.7) | 22821.8 (19815.2-25787.6) | 0.341 (0.281-0.401) |
| Barbados | 1645.6 (1357.8-1946.3) | 7700.3 (6353.2-9107) | 3331.1 (2750-3877.6) | 10394.7 (8581.3-12100) | 0.928 (0.852-1.004) |
| Belarus | 126444.2 (104728.2-147746.8) | 18174.8 (15053.4-21236.8) | 147491.6 (124746.8-171521.9) | 15457.7 (13073.9-17976.1) | -0.444 (-0.674--0.214) |
| Belgium | 184749.1 (161783.8-212008.8) | 19226.4 (16836.5-22063.3) | 338628 (294361.6-386757.5) | 21060.5 (18307.4-24053.9) | 0.449 (0.406-0.492) |
| Belize | 547.3 (449.5-648.3) | 10390 (8532.6-12306.9) | 1795 (1509.7-2091.1) | 12856.7 (10813-14977.3) | 0.821 (0.769-0.873) |
| Benin | 9191.1 (7572.6-10911) | 8959.7 (7382-10636.3) | 22547.4 (18787.2-26761.4) | 10074.5 (8394.4-11957.4) | 0.415 (0.336-0.493) |
| Bermuda | 323.8 (260.8-383.9) | 9319.5 (7506.9-11050.7) | 1156 (953.2-1394) | 12831.6 (10580.8-15472.5) | 1.035 (0.940-1.131) |
| Bhutan | 1885.4 (1627.6-2139.1) | 21027.7 (18152.3-23857.1) | 7236.3 (6219.4-8186.6) | 22446.8 (19292.5-25394.7) | 0.287 (0.266-0.307) |
| Bolivia | 20761.8 (17070.5-24641.5) | 13831.2 (11372.1-16415.8) | 72617.2 (59358.7-84658.7) | 15692.5 (12827.3-18294.6) | 0.529 (0.471-0.587) |
| Bosnia and Herzegovina | 31993.1 (26385.8-37885.1) | 19082.1 (15737.6-22596.3) | 83359.5 (71000.4-95442.2) | 21451.8 (18271.3-24561.2) | 0.619 (0.513-0.725) |
| Botswana | 2693.2 (2202.9-3155.2) | 11725.1 (9590.7-13736.4) | 7734.9 (6388.9-9036.7) | 13210.4 (10911.6-15433.7) | 0.398 (0.271-0.525) |
| Brazil | 807036.7 (667583-937862.6) | 18987.7 (15706.6-22065.7) | 2953925.3 (2484365-3411173.4) | 21007.3 (17667.9-24259) | 0.218 (0.173-0.262) |
| Brunei | 899.6 (774.2-1020.1) | 20198.8 (17382.6-22904.7) | 1956.4 (1651-2294.4) | 14337 (12099.2-16814.4) | -1.295 (-1.409--1.180) |
| Bulgaria | 114922.5 (96771.7-132501.9) | 17425.6 (14673.4-20091.1) | 193049.9 (161946.9-225959.8) | 18753.3 (15731.9-21950.3) | 0.385 (0.291-0.480) |
| Burkina Faso | 15509.6 (12619.5-18449.4) | 7900.1 (6428-9397.6) | 39435.4 (32890.6-47184.9) | 9362.7 (7808.8-11202.5) | 0.539 (0.489-0.589) |
| Burundi | 10779.3 (8744.1-12954.8) | 9526.8 (7728.1-11449.6) | 20175.5 (16588.5-24034.9) | 11199.7 (9208.5-13342.1) | 0.399 (0.322-0.476) |
| Cambodia | 25553.4 (20962.1-30438.4) | 13702.4 (11240.5-16321.9) | 84134.4 (68814.9-98879.3) | 15379.4 (12579.1-18074.7) | 0.732 (0.547-0.918) |
| Cameroon | 14851.3 (12216.8-17756.2) | 8066.9 (6636-9644.8) | 45207.6 (36743.1-53413.4) | 9036.2 (7344.3-10676.4) | 0.404 (0.359-0.448) |
| Canada | 356469.9 (297865.6-415557.6) | 17723.1 (14809.4-20660.9) | 926367 (800056-1050028.5) | 19224.1 (16602.8-21790.3) | 0.327 (0.200-0.453) |
| Cape Verde | 785.8 (639.4-944.6) | 5255.5 (4276.4-6317.7) | 1325.3 (1084.4-1575.7) | 6469.9 (5294.2-7692.4) | 0.109 (-0.242-0.462) |
| Central African Republic | 4132.2 (3398.3-4928.4) | 11121.7 (9146.5-13264.6) | 8461 (6914.5-10104.4) | 13260.9 (10837.2-15836.6) | 0.525 (0.469-0.580) |
| Chad | 13761 (11190.9-16823.2) | 9136.8 (7430.4-11170) | 25531.6 (20754.9-30015.7) | 10713.1 (8708.8-12594.7) | 0.511 (0.418-0.603) |
| Chile | 60779.6 (50234.7-72737.4) | 11249.2 (9297.5-13462.4) | 174913.8 (146767.3-203889.1) | 11229.4 (9422.4-13089.6) | -0.002 (-0.058-0.053) |
| China | 7419682.4 (6442718.7-8299028.8) | 19770.2 (17167-22113.3) | 24270972.6 (20647891.2-27881420.1) | 20343.8 (17306.9-23370) | 0.194 (0.131-0.256) |
| Colombia | 145503.7 (127588-162665.5) | 17394.5 (15252.7-19446.1) | 697438.5 (613244.8-779051.9) | 21885.4 (19243.5-24446.4) | 0.659 (0.610-0.709) |
| Comoros | 686.6 (558.2-809.9) | 8474.2 (6889.3-9995.4) | 2096.4 (1730.1-2507.6) | 9239.7 (7625.2-11051.9) | 0.21 (0.120-0.300) |
| Congo | 4102.1 (3349.1-4955.3) | 9407.9 (7680.9-11364.8) | 10886.2 (8819.4-13005.6) | 11319.4 (9170.4-13523.2) | 0.577 (0.496-0.657) |
| Cook Islands | 85.4 (70.4-101.4) | 14523.9 (11976.4-17240.5) | 223 (179.5-268.2) | 14319.9 (11524.4-17221.2) | -0.128 (-0.215--0.041) |
| Costa Rica | 15403.4 (12935.7-18089.3) | 16261.6 (13656.4-19097.1) | 63810.6 (54839.2-72740.4) | 20347.7 (17487-23195.2) | 0.719 (0.673-0.765) |
| Cote d'Ivoire | 10940.8 (8976.9-13126) | 8176.7 (6709-9809.9) | 40561.5 (33216.7-49230.6) | 9727.9 (7966.4-11807) | 0.675 (0.560-0.791) |
| Croatia | 47761.6 (39841.8-55837.3) | 16850.5 (14056.3-19699.6) | 136530.1 (119012.9-154702.7) | 22206.2 (19357.1-25162) | 1.22 (1.105-1.335) |
| Cuba | 75315.8 (62884.2-88479.5) | 12036 (10049.3-14139.7) | 210813.3 (179981.8-245773.4) | 16977.2 (14494.3-19792.6) | 1.195 (1.056-1.333) |
| Cyprus | 9764.9 (8199.2-11475.3) | 18935 (15899-22251.6) | 27577.5 (23435.8-31482.7) | 20369.8 (17310.6-23254.4) | 0.214 (0.118-0.310) |
| Czech Republic | 119938.5 (98134.9-141846.5) | 15048.9 (12313.2-17797.7) | 307233.3 (262942.3-345959.7) | 19893.5 (17025.7-22401.1) | 1.122 (1.036-1.208) |
| Democratic Republic of the Congo | 57902.6 (47105-69112.5) | 10232.5 (8324.3-12213.5) | 169722.2 (139671.9-202994) | 13422.7 (11046.1-16054) | 0.886 (0.856-0.916) |
| Denmark | 122705.3 (106362.7-139823) | 21951.1 (19027.5-25013.3) | 188776.4 (162143.3-212264.7) | 21958.8 (18860.8-24691) | -0.163 (-0.246--0.079) |
| Djibouti | 309 (254-375.4) | 7269.8 (5975.9-8831.6) | 1812 (1477.2-2167) | 8273.6 (6745-9894.6) | 0.327 (0.156-0.499) |
| Dominica | 345.9 (282.5-413) | 8969.3 (7326.9-10709.4) | 493.7 (404.3-587.8) | 10940.9 (8959-13025.8) | 0.601 (0.564-0.637) |
| Dominican Republic | 16985.1 (13862.4-20463.1) | 9211.9 (7518.3-11098.1) | 71575.6 (59491.3-85056.1) | 12898.4 (10720.7-15327.7) | 1.097 (1.043-1.151) |
| Ecuador | 33783.5 (27763.8-40264.3) | 12474.9 (10252.1-14868) | 143536 (120826-169113.4) | 15353.2 (12924-18089) | 0.687 (0.652-0.721) |
| Egypt | 110694.5 (89868-132042.6) | 11261.7 (9142.9-13433.6) | 373262.2 (308495.3-442308.8) | 16441.4 (13588.6-19482.8) | 1.183 (1.134-1.231) |
| El Salvador | 21225.9 (17602.5-24929) | 13396.5 (11109.7-15733.8) | 68517.2 (57346.3-80826.9) | 17893 (14975.8-21107.7) | 0.973 (0.940-1.007) |
| Equatorial Guinea | 812.6 (667.1-960.3) | 10829.7 (8890.7-12798.3) | 2290.6 (1846.6-2740.9) | 11964.6 (9645.6-14316.5) | 0.364 (0.185-0.542) |
| Eritrea | 2216.5 (1813.7-2659.5) | 7870.3 (6440.3-9443.5) | 8025.4 (6542.5-9685.1) | 8742 (7126.8-10550) | 0.254 (0.140-0.369) |
| Estonia | 10728.1 (9019.8-12618) | 9041.6 (7601.8-10634.3) | 23876.8 (20422.4-27503.6) | 12478.4 (10673.1-14373.8) | 1.485 (1.349-1.620) |
| Ethiopia | 77706.6 (63357.8-93453) | 10054.5 (8197.9-12092) | 209859 (172767.6-246406.9) | 10644.2 (8762.9-12498) | 0.084 (-0.033-0.201) |
| Federated States of Micronesia | 388.3 (318.1-461.7) | 16531.4 (13544-19658.4) | 362.8 (299.1-435.1) | 14360.2 (11839.1-17224.4) | -0.393 (-0.425--0.361) |
| Fiji | 1912.6 (1567.1-2284.2) | 14143.3 (11588.2-16891.6) | 3587.8 (2941.3-4275.6) | 11151.3 (9142-13289.2) | -0.861 (-0.924--0.799) |
| Finland | 67964 (60082.8-76803.6) | 14860.2 (13137-16792.9) | 164591.9 (140917.6-192592) | 17568.4 (15041.4-20557.1) | 0.487 (0.418-0.555) |
| France | 951069.7 (802020.4-1092618.6) | 18278.4 (15413.8-20998.8) | 1878768.5 (1651633.4-2154498) | 18915.4 (16628.6-21691.4) | 0.321 (0.213-0.429) |
| Gabon | 2421.9 (1954-2869.6) | 8581.5 (6923.5-10167.6) | 4249 (3450.9-5111.3) | 10486.3 (8516.5-12614.4) | 0.681 (0.534-0.829) |
| Georgia | 43052.5 (35761.9-50537.9) | 13041.8 (10833.3-15309.4) | 63734.8 (52827-75186.2) | 17086.8 (14162.5-20156.8) | 0.983 (0.800-1.166) |
| Germany | 1860461.9 (1598766-2127405.4) | 23013.1 (19776-26315.1) | 3302061.2 (2909873.4-3687975.4) | 24359.6 (21466.4-27206.6) | 0.261 (0.054-0.468) |
| Ghana | 19845.3 (16397.3-23821.8) | 7831.4 (6470.8-9400.7) | 68223.3 (56058.2-81408.6) | 9910.3 (8143.2-11825.6) | 0.839 (0.785-0.893) |
| Greece | 190306.8 (159255.8-221303.3) | 20088.9 (16811.1-23360.9) | 407483.8 (355607.8-466824.2) | 23536.6 (20540.2-26964.2) | 0.517 (0.414-0.620) |
| Greenland | 274.7 (242.3-307) | 22132.9 (19523.4-24735.2) | 630.7 (556.2-707.5) | 20972.4 (18492.4-23525.2) | -0.157 (-0.179--0.135) |
| Grenada | 453.5 (378-539.6) | 9115.1 (7597.8-10845.5) | 627.3 (527-729.3) | 10612.1 (8914.5-12337.8) | 0.274 (0.131-0.416) |
| Guam | 350 (276.6-431.5) | 12572.2 (9935.4-15498.8) | 1646.1 (1358.8-1939.7) | 13452.7 (11104.8-15852.3) | 0.393 (0.341-0.444) |
| Guatemala | 18749.9 (15449-22301.2) | 12779.2 (10529.4-15199.5) | 95624.3 (79721.4-112129.2) | 16222.2 (13524.3-19022.2) | 0.846 (0.821-0.871) |
| Guinea | 14462.2 (11752.3-17228.2) | 8721.9 (7087.6-10390) | 28093.9 (22838.5-33164.3) | 10825.4 (8800.4-12779.2) | 0.833 (0.743-0.922) |
| Guinea-Bissau | 1362.5 (1123.3-1659) | 8330.8 (6867.9-10143.7) | 2402.8 (1929.6-2870.3) | 9465.6 (7601.4-11307.2) | 0.433 (0.311-0.555) |
| Guyana | 1375 (1123.4-1633.9) | 7835.2 (6401.8-9310.5) | 2836.4 (2323.6-3319.1) | 9802.7 (8030.5-11471.2) | 0.733 (0.712-0.753) |
| Haiti | 14567.1 (11838.1-17381) | 11015.3 (8951.7-13143.1) | 36573.2 (30044.9-43230.1) | 12868.3 (10571.3-15210.5) | 0.466 (0.408-0.524) |
| Honduras | 15224.3 (12413.5-17955.1) | 15602.9 (12722.3-18401.7) | 62270.8 (51719.8-73226.1) | 19759.9 (16411.8-23236.3) | 0.715 (0.660-0.769) |
| Hungary | 154377.6 (127444.8-180608.5) | 18209.5 (15032.7-21303.6) | 305779.5 (265011.9-345879.5) | 22704.5 (19677.5-25682) | 0.908 (0.848-0.969) |
| Iceland | 4565.7 (3988.4-5149.7) | 24988.5 (21829.2-28185.1) | 8973.7 (7733.5-10139.7) | 23719 (20441-26801) | -0.193 (-0.358--0.029) |
| India | 3817123.6 (3340953.4-4271306.1) | 21192.4 (18548.7-23714) | 13871313.1 (12096354.3-15354507.7) | 23280.3 (20301.4-25769.6) | 0.361 (0.337-0.385) |
| Indonesia | 486701.8 (397693.5-574942.3) | 12780.8 (10443.4-15098) | 1487122.2 (1204091.8-1749065.6) | 15417.1 (12482.9-18132.7) | 0.609 (0.594-0.625) |
| Iran | 95274.2 (78635.2-112843.7) | 9819 (8104.2-11629.7) | 592441.7 (497588.2-695729.5) | 16223.9 (13626.4-19052.4) | 1.912 (1.827-1.997) |
| Iraq | 44417.8 (36362.1-52634.3) | 11119 (9102.4-13175.8) | 138773.7 (116070.2-164536.1) | 13991.9 (11702.8-16589.4) | 0.861 (0.785-0.936) |
| Ireland | 56992.2 (48423.5-63981) | 21209.9 (18021-23810.8) | 111395.6 (96633.2-126986.7) | 21428.4 (18588.7-24427.5) | 0.06 (0.011-0.109) |
| Israel | 59256.4 (50487.7-68359.2) | 19491.2 (16606.9-22485.4) | 157314.4 (132688-181793.3) | 19054.1 (16071.3-22019) | 0.07 (-0.002-0.142) |
| Italy | 1040605.9 (864207.7-1210173.9) | 18786.5 (15601.9-21847.8) | 2227962.2 (1894875.2-2553690) | 21185.3 (18018-24282.5) | 0.465 (0.428-0.503) |
| Jamaica | 12037.8 (9991.9-14094.6) | 10347.6 (8589-12115.5) | 24076 (20872.6-27892.5) | 13237.3 (11476.1-15335.8) | 0.865 (0.774-0.956) |
| Japan | 1424946.8 (1171156.1-1684406) | 14426.6 (11857.1-17053.4) | 4059349.4 (3369218.2-4822024.7) | 14045.2 (11657.3-16684) | 0.031 (-0.036-0.098) |
| Jordan | 6470.4 (5357.3-7583.9) | 13192.6 (10923.1-15462.9) | 47200 (39429.7-55373.6) | 15369.2 (12839-18030.7) | 0.493 (0.436-0.550) |
| Kazakhstan | 104102.5 (85926-124428.3) | 17351.2 (14321.7-20739) | 170712 (145796.3-195887.9) | 20990.1 (17926.6-24085.6) | 0.923 (0.637-1.210) |
| Kenya | 31039 (25507.3-37044.8) | 8376.8 (6883.9-9997.6) | 87927.5 (72017.4-104241.4) | 9423.2 (7718.1-11171.5) | 0.254 (0.158-0.350) |
| Kiribati | 218.4 (176.7-263.6) | 15147.3 (12256.6-18278.5) | 370.2 (304.1-440.3) | 14876.1 (12219.3-17694.6) | -0.007 (-0.053-0.040) |
| Kuwait | 2240.7 (1880-2642) | 11221.7 (9414.9-13231) | 16621 (13777.2-19465.3) | 16834.5 (13954.2-19715.3) | 1.485 (1.348-1.622) |
| Kyrgyzstan | 32410.5 (27808.5-36767.1) | 22412.1 (19229.8-25424.7) | 40320.9 (33701.7-46682.1) | 21007 (17558.4-24321.2) | -0.139 (-0.300-0.022) |
| Laos | 13695.4 (11350.9-16184.3) | 15433.3 (12791.2-18237.9) | 32303 (26799.3-38193.9) | 16769.4 (13912.3-19827.5) | 0.231 (0.191-0.272) |
| Latvia | 25566.8 (20904.6-30448.9) | 12322.6 (10075.6-14675.7) | 39759 (33587-46278.6) | 14119.9 (11928-16435.3) | 0.499 (0.247-0.751) |
| Lebanon | 11949.9 (9992.1-14247.7) | 12495.3 (10448.1-14898) | 77540.8 (64916.9-91635.3) | 20164.7 (16881.8-23830) | 1.792 (1.713-1.872) |
| Lesotho | 5697.5 (4646.6-6760.7) | 13281.9 (10832.1-15760.5) | 6862.6 (5583.4-8210.5) | 14273.9 (11613.3-17077.5) | 0.165 (0.076-0.255) |
| Liberia | 4814.3 (3936.2-5730.2) | 8091.6 (6615.7-9630.9) | 8906.7 (7351.1-10535.8) | 11168.4 (9217.7-13211.1) | 0.959 (0.888-1.029) |
| Libya | 10314.9 (8508.1-12265.4) | 11868.9 (9789.9-14113.2) | 35990.3 (29533.2-42643.2) | 16423.2 (13476.7-19459.1) | 1.144 (1.108-1.180) |
| Lithuania | 38145.1 (31458.5-45020.5) | 15029.3 (12394.8-17738.2) | 59308.9 (51343-67917.4) | 14766.7 (12783.3-16910) | -0.07 (-0.279-0.139) |
| Luxembourg | 7119 (6207-8105.8) | 20846.5 (18175.9-23735.9) | 14816 (12789.7-16975.4) | 22292.8 (19243.8-25541.8) | 0.246 (0.217-0.274) |
| Macedonia | 15919.2 (13181.1-18887.7) | 18063.4 (14956.5-21431.8) | 37915.4 (31778.5-44663.4) | 19956.2 (16726.1-23507.9) | 0.531 (0.443-0.620) |
| Madagascar | 19661.3 (16044.5-23574.2) | 9158.8 (7474-10981.6) | 40199.2 (33367.6-47835.8) | 11051.9 (9173.7-13151.4) | 0.551 (0.515-0.588) |
| Malawi | 10884.5 (8974.5-13040.7) | 6998.4 (5770.3-8384.7) | 27289.4 (22657.5-32090.5) | 8772.2 (7283.3-10315.5) | 0.704 (0.657-0.752) |
| Malaysia | 55405.4 (45771.6-65783.5) | 12732.6 (10518.7-15117.5) | 181186.7 (149157.1-215582.7) | 12813 (10548-15245.4) | -0.109 (-0.168--0.049) |
| Maldives | 473.3 (383.5-564.8) | 15776.3 (12783.3-18823.9) | 2591.4 (2145.1-3005.2) | 19052.8 (15771.6-22095.8) | 0.513 (0.372-0.654) |
| Mali | 12714.8 (10487.6-15284.4) | 7824.8 (6454.1-9406.2) | 35964.2 (29605.5-42793.6) | 9741.5 (8019.2-11591.4) | 0.714 (0.611-0.818) |
| Malta | 4393.6 (3765.6-4968.2) | 17696.5 (15167-20010.8) | 12326.5 (10492.1-14240.7) | 17169.8 (14614.6-19836.2) | -0.128 (-0.194--0.062) |
| Marshall Islands | 108.4 (87.8-130.9) | 14764.4 (11956.9-17827.3) | 137.6 (111.9-166.1) | 11986 (9747.1-14469.3) | -0.703 (-0.740--0.666) |
| Mauritania | 3709.4 (3031.3-4450) | 7109.2 (5809.8-8528.8) | 9367 (7720.9-11213.4) | 9201.6 (7584.6-11015.4) | 0.808 (0.718-0.899) |
| Mauritius | 3509 (2909.3-4075.1) | 10655.8 (8834.6-12374.7) | 12342.5 (10623.2-14223.7) | 12308.2 (10593.6-14184.1) | 0.417 (0.386-0.448) |
| Mexico | 335283.4 (276380.9-393824.4) | 16144.3 (13308.1-18963.2) | 1332906.5 (1125773.3-1547069.4) | 19727 (16661.4-22896.6) | 0.665 (0.573-0.758) |
| Moldova | 34099.7 (28820.8-39460.9) | 16234.4 (13721.2-18786.8) | 54206.7 (45220.1-64275.1) | 15555.1 (12976.3-18444.3) | -0.149 (-0.282--0.016) |
| Monaco | 1017.9 (867.8-1187.6) | 20543.7 (17515.9-23970.5) | 1447.4 (1231.3-1692.9) | 20331.6 (17296.4-23780.1) | 0.053 (-0.073-0.180) |
| Mongolia | 7822.2 (6446.9-9490.9) | 15222 (12545.7-18469.2) | 14068.9 (11695.9-16631) | 16721.4 (13901.1-19766.6) | 0.293 (0.189-0.397) |
| Montenegro | 4464.7 (3722.2-5282.3) | 13950.7 (11630.6-16505.4) | 9308.7 (7711.3-10818.2) | 16001.1 (13255.3-18595.8) | 0.697 (0.587-0.808) |
| Morocco | 77867.5 (63521.4-92359.4) | 10977.1 (8954.7-13020.1) | 276901.1 (228095.6-327361.2) | 16755.9 (13802.6-19809.4) | 1.375 (1.333-1.418) |
| Mozambique | 19094.6 (15593.3-23142.8) | 7856.8 (6416.1-9522.5) | 38901.3 (31810.5-45819.8) | 9049.3 (7399.8-10658.7) | 0.324 (0.258-0.390) |
| Myanmar | 181600.8 (152524.3-210513.8) | 18567.3 (15594.5-21523.5) | 445102.3 (375575.1-506434.3) | 19917.8 (16806.6-22662.4) | 0.299 (0.258-0.339) |
| Namibia | 3171.5 (2599.8-3812.2) | 11943.5 (9790.6-14356.3) | 8003.8 (6594.8-9538.2) | 13369.6 (11015.9-15932.6) | 0.411 (0.318-0.505) |
| Nauru | 22.8 (18.5-27.4) | 14429.4 (11735.8-17338) | 30.6 (24.6-37.2) | 13510.4 (10859.6-16463.3) | -0.229 (-0.250--0.209) |
| Nepal | 76293.4 (67761.1-83656.3) | 20924 (18583.9-22943.3) | 261227.8 (242581.3-277064.3) | 22422.6 (20822-23781.9) | 0.329 (0.269-0.390) |
| Netherlands | 218963.2 (190422.9-246601.9) | 17069 (14844.1-19223.5) | 441955.6 (384505.3-503254.8) | 17995.2 (15656-20491.1) | 0.586 (0.374-0.799) |
| New Zealand | 39923.6 (32353-47972.8) | 16135.3 (13075.6-19388.4) | 83464.4 (66736.9-101937.6) | 14918.7 (11928.8-18220.6) | -0.142 (-0.290-0.007) |
| Nicaragua | 10886.5 (9030-12920.7) | 14792.1 (12269.6-17556.1) | 50242.3 (43485.5-57704.4) | 20122.4 (17416.3-23111) | 1.048 (0.952-1.143) |
| Niger | 8948.4 (7308.9-10767) | 8704.3 (7109.4-10473.3) | 38180.5 (31353.8-45516.7) | 11205.6 (9202-13358.7) | 0.952 (0.857-1.047) |
| Nigeria | 182493.9 (150657.1-216602.3) | 8710.3 (7190.7-10338.3) | 421054.3 (347560.3-491858.9) | 11245.2 (9282.4-13136.2) | 0.898 (0.842-0.954) |
| Niue | 24 (19.4-28.2) | 16346.7 (13237.2-19209.5) | 16.8 (13.8-20.6) | 14180.7 (11594.3-17321.6) | -0.507 (-0.612--0.403) |
| North Korea | 151092 (135229.6-166670.5) | 23076 (20653.3-25455.2) | 369265.3 (318570.2-422178.9) | 21100 (18203.2-24123.5) | -0.353 (-0.377--0.329) |
| Northern Mariana Islands | 64.6 (51.5-78.1) | 13444.3 (10725.6-16260.8) | 271.5 (220.4-329.8) | 12820 (10408.8-15572.2) | -0.03 (-0.147-0.086) |
| Norway | 95757.9 (81129.9-110208.9) | 19598.7 (16604.8-22556.4) | 167363.8 (142331.8-190203.4) | 24175.6 (20559.8-27474.8) | 0.822 (0.593-1.052) |
| Oman | 3810.5 (3134.4-4558.1) | 13203.6 (10860.8-15793.8) | 11912.1 (9799.4-14105.6) | 17252.4 (14192.5-20429.3) | 0.771 (0.705-0.837) |
| Pakistan | 549059.5 (460568.8-638718.2) | 19421.8 (16291.6-22593.3) | 1015788.4 (842567.6-1175472.2) | 20060.1 (16639.3-23213.6) | 0.132 (0.081-0.183) |
| Palau | 68.5 (55.3-82.1) | 14325.2 (11563.3-17189.2) | 114.3 (92.9-137.9) | 12541.3 (10192.2-15123.5) | -0.394 (-0.499--0.288) |
| Palestine | 5401.8 (4448.3-6441.9) | 12808.2 (10547.4-15274.4) | 16051 (13402.8-18947.7) | 15461.6 (12910.7-18252.1) | 0.706 (0.605-0.808) |
| Panama | 10629.8 (8762.4-12521.7) | 13172.6 (10858.5-15517.1) | 46023.4 (39981.8-52016.4) | 17635.1 (15320.1-19931.5) | 0.803 (0.689-0.918) |
| Papua New Guinea | 11177.1 (9737.2-12540.8) | 18616.4 (16218-20887.8) | 31958.3 (27706.2-36115) | 18363.7 (15920.4-20752.2) | -0.024 (-0.094-0.047) |
| Paraguay | 17259.3 (14220-20411.9) | 14884.2 (12263.1-17603) | 54206.8 (45921.8-62877.9) | 17955.8 (15211.4-20828.1) | 0.645 (0.619-0.670) |
| Peru | 64417.1 (53437.4-77619.3) | 10744.6 (8913.2-12946.7) | 257041.9 (215474.9-303443.6) | 13653 (11445.1-16117.7) | 1.007 (0.895-1.119) |
| Philippines | 223202.7 (184873.4-260147) | 17223 (14265.4-20073.7) | 553063.8 (460074.6-644945) | 15288.7 (12718.2-17828.7) | -0.671 (-0.772--0.571) |
| Poland | 391878.2 (325090-456494.9) | 16285.4 (13509.8-18970.7) | 851826.3 (716673.4-978411.9) | 18237.5 (15343.9-20947.7) | 0.501 (0.420-0.582) |
| Portugal | 167484.9 (148736.6-184180.5) | 19483.9 (17302.8-21426.1) | 365492.5 (318629.7-418411.6) | 20654.5 (18006.2-23645) | 0.191 (0.162-0.220) |
| Puerto Rico | 25513.9 (21241.1-29693.8) | 11414.9 (9503.3-13285) | 82122 (69464.9-93104.9) | 15591.6 (13188.5-17676.8) | 0.909 (0.785-1.034) |
| Qatar | 335.8 (278.2-396.6) | 13911.1 (11525.6-16430.7) | 3210.4 (2719.4-3763.3) | 18281.2 (15485.2-21429.4) | 0.972 (0.894-1.050) |
| Romania | 266949.5 (222268-317339.8) | 18707.6 (15576.4-22238.9) | 478912.7 (410026.3-550010.7) | 18918.6 (16197.3-21727.2) | 0.149 (0.021-0.277) |
| Russian Federation | 1583507.5 (1305529.7-1856822.7) | 16399.6 (13520.7-19230.1) | 2211893 (1858485.3-2555694.1) | 15459.9 (12989.8-17862.9) | -0.217 (-0.372--0.062) |
| Rwanda | 10316.4 (8316-12379.7) | 9342.8 (7531.2-11211.4) | 27363.6 (22830-32019.8) | 11074.3 (9239.5-12958.6) | 0.485 (0.396-0.574) |
| Saint Kitts and Nevis | 206.3 (170.6-240.7) | 7632.6 (6310.7-8905.7) | 299.5 (248.1-350.9) | 10778.2 (8929.4-12627.1) | 1.205 (1.002-1.408) |
| Saint Lucia | 546.9 (452.6-650.3) | 10462.2 (8657.9-12440.5) | 1809.9 (1499.8-2094.2) | 13324.6 (11041.3-15417.5) | 0.844 (0.764-0.924) |
| Saint Vincent and the Grenadines | 330.2 (269.9-388.5) | 7568.8 (6185-8903.5) | 790.4 (668.7-920.8) | 9934.9 (8404.8-11572.9) | 0.948 (0.929-0.967) |
| Samoa | 675.7 (568-793.5) | 17831 (14989-20941.7) | 1117.7 (915.7-1318.4) | 16359.1 (13402.8-19296.8) | -0.2 (-0.234--0.167) |
| San Marino | 446.7 (376.7-520.3) | 19124.3 (16129.9-22276) | 1045.1 (877.4-1218.6) | 20060.5 (16840.9-23390) | 0.183 (0.108-0.258) |
| Sao Tome and Principe | 327 (270.3-388.2) | 9896.8 (8181.5-11751.5) | 576.7 (480.4-678.3) | 12580.7 (10478.4-14795.8) | 0.806 (0.783-0.828) |
| Saudi Arabia | 24212.1 (19818.7-29015.4) | 9753.3 (7983.5-11688.2) | 72472.5 (60732.3-85518) | 13964.6 (11702.4-16478.3) | 1.328 (1.239-1.418) |
| Senegal | 12720.3 (10483.1-14921) | 8411.5 (6932.2-9866.8) | 35548.9 (29380.8-42112.4) | 10040.1 (8298-11893.8) | 0.667 (0.573-0.761) |
| Serbia | 82643.1 (70248.4-96409.4) | 16639.7 (14144.1-19411.5) | 237726.6 (201652.4-271494.6) | 21987.8 (18651.2-25111) | 1.198 (1.115-1.281) |
| Seychelles | 372.9 (303.1-449.8) | 10900.2 (8858.4-13146.4) | 750 (615.5-905.9) | 13580.9 (11145.1-16403.6) | 0.595 (0.549-0.641) |
| Sierra Leone | 9472.1 (7796.3-11368.9) | 8767.9 (7216.7-10523.6) | 17763.5 (14735.6-21374.4) | 10359.7 (8593.8-12465.6) | 0.479 (0.415-0.543) |
| Singapore | 12455.3 (10220.9-14835.1) | 11899.7 (9764.9-14173.2) | 40700.2 (34116.1-48007.4) | 8423.3 (7060.7-9935.6) | -1.208 (-1.313--1.102) |
| Slovakia | 46023.6 (38459.2-54674.7) | 13922.2 (11634-16539.2) | 99410.7 (85008.9-115867.8) | 16535.1 (14139.6-19272.4) | 0.799 (0.723-0.874) |
| Slovenia | 23119.7 (19458.7-26887.2) | 16882.6 (14209.3-19633.8) | 58496.4 (50108.6-68175.9) | 19467.9 (16676.4-22689.3) | 0.621 (0.528-0.714) |
| Solomon Islands | 819.4 (693.2-960.5) | 17454.8 (14766.2-20461.4) | 2214.2 (1850.8-2611.4) | 16707.5 (13965.5-19704.8) | -0.106 (-0.136--0.076) |
| Somalia | 7210.1 (5859.7-8683.7) | 10274.1 (8349.8-12373.9) | 22923.9 (18796-27350.9) | 12032.4 (9865.7-14356) | 0.426 (0.316-0.536) |
| South Africa | 152388.6 (128031.7-177621.3) | 15019.9 (12619.2-17506.9) | 343193.8 (285582.8-398980.9) | 15511.8 (12907.9-18033.3) | 0.092 (0.062-0.122) |
| South Korea | 214609.3 (178397.7-250903.8) | 16906.7 (14054-19765.9) | 1082247.4 (958960.7-1202029.3) | 19534.5 (17309.2-21696.5) | 0.571 (0.399-0.744) |
| South Sudan | 10304 (8523.1-12322.3) | 8199.4 (6782.3-9805.5) | 13528.3 (10905.7-16169.7) | 9718.7 (7834.6-11616.2) | 0.387 (0.317-0.457) |
| Spain | 749068.4 (662800.6-845161.8) | 21733.2 (19230.2-24521.2) | 1626246.1 (1429387.8-1836589.8) | 24033.2 (21123.9-27141.7) | 0.38 (0.342-0.418) |
| Sri Lanka | 58217.4 (47094.1-70186.6) | 11805.4 (9549.8-14232.5) | 213074.4 (175450.8-254322.8) | 14004.3 (11531.5-16715.3) | 0.468 (0.414-0.522) |
| Sudan | 65832.1 (54231.5-78986.5) | 13968.6 (11507.1-16759.8) | 144389 (120227.1-170565.7) | 16864.3 (14042.3-19921.7) | 0.505 (0.437-0.573) |
| Suriname | 1199.6 (1007.9-1404.9) | 9821.1 (8251.5-11501.3) | 3899 (3241.9-4647.6) | 11718.7 (9743.5-13968.5) | 0.541 (0.521-0.561) |
| Swaziland | 1373.8 (1122.1-1645.7) | 11620 (9491-13919.9) | 2613.5 (2153.6-3127.2) | 11624.2 (9578.8-13909.1) | -0.141 (-0.269--0.014) |
| Sweden | 278605.6 (242595.4-316642.3) | 25524.1 (22225.1-29008.8) | 442574.4 (384168.8-496918.7) | 27750.7 (24088.5-31158.3) | 0.343 (0.161-0.525) |
| Switzerland | 151850.5 (131560.5-173019.2) | 21769.6 (18860.8-24804.4) | 278998 (243190.1-316573.7) | 22362.3 (19492.2-25374.1) | 0.011 (-0.106-0.128) |
| Syria | 28439.5 (23642-33508) | 12765.4 (10612-15040.4) | 99868 (83582-117896.8) | 16912.1 (14154.2-19965.2) | 1.059 (1.002-1.116) |
| Taiwan | 108702.7 (93248.4-124970.7) | 14904.9 (12785.9-17135.5) | 343729.6 (293044.7-409050.8) | 13610.3 (11603.4-16196.8) | -0.176 (-0.255--0.096) |
| Tajikistan | 22537.5 (18150.7-26952) | 17597.6 (14172.4-21044.6) | 37215.8 (30956.9-43891.3) | 18706.3 (15560.3-22061.7) | 0.348 (0.210-0.486) |
| Tanzania | 36814.1 (30245.7-44576.3) | 7556.5 (6208.3-9149.8) | 103241.8 (85368.3-123011.1) | 9295.3 (7686.1-11075.3) | 0.538 (0.429-0.648) |
| Thailand | 212650.7 (179290.3-243571.3) | 13911.6 (11729.2-15934.5) | 811749.5 (656256.1-968655.2) | 13029.4 (10533.6-15547.9) | -0.157 (-0.221--0.094) |
| The Bahamas | 614.1 (498.1-727.9) | 7596.6 (6161.3-9003.8) | 1911.8 (1604.5-2224) | 9864.2 (8278.6-11474.7) | 0.804 (0.709-0.900) |
| The Gambia | 1358.5 (1122-1628.5) | 8805.2 (7272.3-10554.9) | 4742.1 (3904.1-5663.7) | 10578.6 (8709.1-12634.4) | 0.613 (0.509-0.718) |
| Timor-Leste | 1339.4 (1114.1-1594.9) | 15697.8 (13057-18692.5) | 7110.2 (5920.3-8426.1) | 15723.6 (13092.2-18633.5) | -0.038 (-0.097-0.021) |
| Togo | 4297.4 (3480.6-5085.3) | 8476.8 (6865.7-10030.9) | 13930.1 (11430.6-16590.9) | 9638.3 (7908.9-11479.3) | 0.398 (0.319-0.477) |
| Tokelau | 11.1 (9.1-13.1) | 14693.4 (12086.1-17440.5) | 12.9 (10.3-15.2) | 14059.9 (11289-16644.4) | -0.063 (-0.156-0.029) |
| Tonga | 431 (356.1-503.4) | 16596.3 (13712.6-19382) | 656.6 (531.4-790) | 14986.5 (12128.8-18031.3) | -0.391 (-0.423--0.360) |
| Trinidad and Tobago | 4051.9 (3305.3-4794.3) | 8583.3 (7001.7-10155.9) | 11729.5 (9792.4-13866.8) | 10482.7 (8751.5-12392.8) | 0.616 (0.582-0.650) |
| Tunisia | 35768.6 (29752.7-42190.5) | 14795.5 (12307.1-17452) | 144429.1 (123028.5-166896.4) | 20743.8 (17670.1-23970.6) | 1.163 (1.118-1.208) |
| Turkey | 293510.9 (242583.3-351684) | 19567.1 (16172-23445.3) | 1204448.7 (1047207.1-1370875.8) | 23691.4 (20598.5-26965.1) | 0.748 (0.678-0.818) |
| Turkmenistan | 12254.1 (10078.2-14487.1) | 14473.6 (11903.6-17111.1) | 19998.2 (16762.6-23420) | 12766.5 (10700.9-14950.9) | -0.531 (-0.783--0.277) |
| Tuvalu | 40.9 (33.5-48.7) | 15156.5 (12408.4-18025.3) | 69.7 (56.3-83.2) | 13685.2 (11045.6-16332.8) | -0.321 (-0.382--0.261) |
| Uganda | 27927.9 (22667.5-33481.1) | 9542 (7744.7-11439.4) | 60798.8 (49992.5-72915) | 10104.3 (8308.3-12117.9) | 0.027 (-0.066-0.120) |
| Ukraine | 807219.8 (661711.2-965920.5) | 20138.2 (16508.1-24097.4) | 727380.8 (604316.7-865024.2) | 14915.6 (12392-17738.1) | -1.022 (-1.195--0.849) |
| United Arab Emirates | 1697.6 (1388.8-2014.5) | 13949.8 (11412-16554) | 7338.6 (6082.7-8726.6) | 15200.8 (12599.4-18076) | 0.146 (0.041-0.251) |
| United Kingdom | 1583543.2 (1374066.3-1783991) | 25448.5 (22082.1-28669.9) | 2610913.6 (2318031.1-2882524.7) | 28537.8 (25336.5-31506.5) | 0.487 (0.389-0.585) |
| United States | 5379060.1 (4738013-5992692) | 25362.3 (22339.7-28255.5) | 11405217.8 (10643073.6-12035490.3) | 29634.8 (27654.4-31272.4) | 0.703 (0.494-0.913) |
| Uruguay | 28390.7 (23409.5-33653.9) | 11566.1 (9536.8-13710.3) | 47422.2 (40472.4-55483.6) | 12620 (10770.6-14765.4) | 0.416 (0.311-0.521) |
| Uzbekistan | 90878.2 (74727.4-106541) | 15933.8 (13102.1-18680) | 140736 (116478.6-162326.7) | 14759.9 (12215.9-17024.2) | -0.225 (-0.398--0.052) |
| Vanuatu | 388 (319.8-455.4) | 16856.6 (13894-19785.2) | 1008.1 (819.4-1217.5) | 15142.5 (12308.5-18288.4) | -0.39 (-0.428--0.351) |
| Venezuela | 60742.8 (51654.9-71580.5) | 12795.6 (10881.2-15078.6) | 288545.3 (250404.8-339577) | 17967.4 (15592.5-21145.1) | 1.124 (0.979-1.270) |
| Vietnam | 285426.5 (234224.9-340362.8) | 13998.2 (11487.1-16692.5) | 784298 (657162.9-921683.8) | 17554.9 (14709.2-20629.9) | 0.733 (0.676-0.790) |
| Virgin Islands, U.S. | 325.7 (267.5-385.9) | 8128.5 (6677.5-9632.5) | 1432.3 (1185.9-1696.4) | 11106.5 (9196.2-13154.5) | 0.843 (0.764-0.921) |
| Yemen | 29382.4 (24261.9-35268.7) | 15061.5 (12436.7-18078.9) | 99836 (80777.7-119266.3) | 16783.4 (13579.5-20049.8) | 0.381 (0.302-0.459) |
| Zambia | 9133.6 (7458.8-10936.1) | 7885 (6439.1-9441) | 24529 (20070.2-28972.5) | 9115.7 (7458.7-10767.1) | 0.379 (0.280-0.479) |
| Zimbabwe | 18381.7 (15003.1-21962) | 10249.8 (8365.9-12246.2) | 29638.7 (24333.8-36116.9) | 11165.1 (9166.8-13605.6) | 0.259 (0.152-0.366) |

EAPC, estimated annual percentage change; UI, uncertainty interval; CI, confidence interval.

**S7 Table. DALYs and EAPC of chronic obstructive pulmonary disease among adults aged ≥70 years in 204 countries and territories, from 1990 to 2021**

|  | **DALYs (95% UI)** | | | |  |
| --- | --- | --- | --- | --- | --- |
|  | **No in 1990** | **Rate in 1990 (per 100,000)** | **No in 2021** | **Rate in 2021 (per 100,000)** | **EAPCs (95% CI)** |
| Afghanistan | 30715.6 (18965.7-41046.2) | 9883.6 (6102.8-13207.8) | 35307.7 (25689.1-45847.8) | 8727.8 (6350.2-11333.2) | -0.549 (-0.718--0.379) |
| Albania | 7847.2 (6688.4-9177.9) | 7861 (6700.1-9194.1) | 9456.6 (7376.9-12079.2) | 3487.1 (2720.2-4454.1) | -2.571 (-2.987--2.152) |
| Algeria | 22972.8 (18617.6-28603.4) | 3688.7 (2989.4-4592.8) | 69649.3 (57017.4-82936.5) | 4025.5 (3295.5-4793.5) | 0.225 (0.163-0.286) |
| American Samoa | 109.9 (95.5-125.2) | 12513.3 (10878.5-14256.2) | 185.1 (158.6-216.9) | 8358.7 (7162.7-9792.6) | -1.383 (-1.461--1.306) |
| Andorra | 204.4 (156.4-267.9) | 6382.8 (4883.5-8365.9) | 399.3 (306.9-492.7) | 4278.8 (3288-5279.6) | -1.024 (-1.305--0.742) |
| Angola | 11285.9 (8301.3-14563) | 8800.1 (6472.9-11355.5) | 22655 (17564.4-28828.7) | 5645.6 (4377-7184) | -1.741 (-1.838--1.645) |
| Antigua and Barbuda | 45.7 (41.9-49.6) | 1257 (1151.1-1364.7) | 94.5 (86.3-103.2) | 1682.2 (1536.5-1837.3) | 0.648 (0.447-0.849) |
| Argentina | 86287.9 (80688-91842.3) | 4666.1 (4363.3-4966.5) | 161413.6 (146171.4-172947.5) | 4529.4 (4101.7-4853.1) | 0.05 (-0.295-0.397) |
| Armenia | 9466.5 (8809-9990.2) | 7924.3 (7373.9-8362.7) | 8663.6 (7780.5-9693.2) | 3543.8 (3182.6-3965) | -2.205 (-2.691--1.716) |
| Australia | 70593.7 (66258.6-74785.1) | 5834.5 (5476.2-6180.9) | 121108.4 (107124.8-131143.4) | 3927.1 (3473.6-4252.5) | -1.291 (-1.465--1.116) |
| Austria | 28117.5 (26037.4-30154.1) | 3688 (3415.2-3955.2) | 46798.5 (41990-51114.4) | 3762.1 (3375.6-4109.1) | 0.254 (0.026-0.482) |
| Azerbaijan | 11311.5 (9704.8-13444.5) | 5035.3 (4320.1-5984.8) | 12940.4 (10745.5-15799) | 3377.1 (2804.3-4123.1) | -1.381 (-1.658--1.104) |
| Bahrain | 636.3 (566.6-712.3) | 11122.1 (9903.3-12450.8) | 1583.1 (1343.1-1849.4) | 6283.9 (5331.5-7340.9) | -2.287 (-2.688--1.885) |
| Bangladesh | 403678.7 (305178.7-502483.5) | 17741.7 (13412.6-22084.1) | 867013.7 (686639.7-1079227.2) | 11764.6 (9317.1-14644.2) | -1.584 (-1.837--1.331) |
| Barbados | 306.9 (277.8-335.8) | 1436.2 (1300-1571.5) | 531.3 (440.3-625.8) | 1658 (1373.9-1952.7) | 0.166 (-0.059-0.390) |
| Belarus | 62403.5 (58455.9-66129.8) | 8969.7 (8402.3-9505.3) | 16576.5 (14487.4-18989.2) | 1737.3 (1518.3-1990.1) | -6.167 (-6.638--5.693) |
| Belgium | 65856.8 (60937.1-69780.7) | 6853.6 (6341.6-7261.9) | 75449.9 (65497.5-81813.8) | 4692.5 (4073.5-5088.3) | -1.302 (-1.418--1.186) |
| Belize | 140 (127.3-151.3) | 2658.2 (2415.6-2872.6) | 544.9 (472.2-618.6) | 3902.5 (3382.2-4430.9) | 0.945 (0.405-1.488) |
| Benin | 6926.1 (5515.7-8256.7) | 6751.7 (5376.9-8048.8) | 10554.4 (8517-13349.1) | 4715.9 (3805.5-5964.6) | -0.937 (-1.169--0.705) |
| Bermuda | 81.7 (74.8-88.8) | 2351.8 (2153.2-2556.4) | 182.1 (157.6-213.3) | 2021.7 (1748.9-2367.9) | -0.566 (-0.767--0.364) |
| Bhutan | 1816.7 (1294.8-2386.6) | 20260.9 (14440.7-26617.1) | 5710.2 (4523.9-7276.3) | 17712.9 (14033.2-22570.9) | -0.495 (-0.598--0.392) |
| Bolivia | 9544.7 (7616.1-11566.7) | 6358.5 (5073.7-7705.5) | 22512.1 (17834.8-28288.8) | 4864.8 (3854.1-6113.2) | -0.583 (-0.674--0.491) |
| Bosnia and Herzegovina | 10751.6 (9385.1-12074.8) | 6412.7 (5597.7-7201.9) | 14641.7 (12125.8-17400.4) | 3767.9 (3120.5-4477.8) | -1.797 (-2.036--1.556) |
| Botswana | 2827.9 (2146.7-3486.8) | 12311.5 (9345.7-15179.9) | 4461.1 (3649.7-5590.9) | 7619.1 (6233.4-9548.7) | -1.58 (-1.685--1.475) |
| Brazil | 334066.3 (312364.9-350139.3) | 7859.8 (7349.2-8237.9) | 712717.4 (636493.2-760540) | 5068.6 (4526.5-5408.7) | -1.738 (-1.966--1.510) |
| Brunei | 562.4 (478.1-671.2) | 12627.6 (10734.7-15071.8) | 830.4 (712.4-966.3) | 6085.7 (5220.5-7081.4) | -2.184 (-2.335--2.032) |
| Bulgaria | 33766.3 (30453.7-37016.2) | 5120 (4617.7-5612.7) | 31156.7 (27082.4-35513.4) | 3026.6 (2630.8-3449.9) | -1.725 (-1.882--1.569) |
| Burkina Faso | 8289.9 (6848.3-10002.7) | 4222.6 (3488.3-5095.1) | 15644.8 (13168.1-19510) | 3714.4 (3126.3-4632) | -0.323 (-0.373--0.273) |
| Burundi | 11872.7 (8853.3-14592.2) | 10493.2 (7824.6-12896.7) | 13043.3 (9639.7-16535) | 7240.5 (5351.1-9178.8) | -1.585 (-1.792--1.378) |
| Cambodia | 17217.7 (13359.7-20791.3) | 9232.6 (7163.8-11148.9) | 44965 (36303.4-54364.5) | 8219.4 (6636.1-9937.6) | -0.371 (-0.423--0.320) |
| Cameroon | 12660.2 (10010.3-15473.2) | 6876.8 (5437.4-8404.7) | 23475.9 (18885-29645.7) | 4692.4 (3774.8-5925.6) | -1.083 (-1.246--0.921) |
| Canada | 106530 (98687.1-113635.4) | 5296.5 (4906.6-5649.8) | 203274 (181041-220857) | 4218.4 (3757-4583.2) | -0.817 (-0.984--0.650) |
| Cape Verde | 1023.3 (831.3-1212.1) | 6843.8 (5559.6-8106.4) | 724.1 (571.7-866.9) | 3535.2 (2790.9-4232.1) | -2.036 (-2.782--1.285) |
| Central African Republic | 4196.1 (2705.9-5570.9) | 11293.7 (7282.8-14993.9) | 6265.3 (3943.6-8936.9) | 9819.6 (6180.9-14006.8) | -0.512 (-0.558--0.466) |
| Chad | 9447 (7021.7-11790.2) | 6272.5 (4662.2-7828.3) | 13853.1 (10558.4-17735.5) | 5812.8 (4430.3-7441.9) | -0.09 (-0.200-0.019) |
| Chile | 24292 (22783.4-25768.5) | 4496 (4216.8-4769.3) | 49933.8 (44015.3-54098.1) | 3205.7 (2825.8-3473.1) | -0.617 (-0.978--0.255) |
| China | 13634189.5 (11795483-15073285.5) | 36329.1 (31429.8-40163.7) | 16279703.9 (13813867.7-19089562.9) | 13645.6 (11578.7-16000.8) | -3.607 (-3.788--3.426) |
| Colombia | 58604.5 (54566.6-61501.8) | 7006 (6523.3-7352.3) | 209840.6 (176435-238602.1) | 6584.7 (5536.5-7487.3) | -0.638 (-0.798--0.479) |
| Comoros | 620.3 (441.9-822.3) | 7655.5 (5454.4-10148.4) | 1164.4 (851.9-1534.6) | 5131.8 (3754.6-6763.5) | -1.458 (-1.671--1.245) |
| Congo | 4300.3 (2972.7-5852.3) | 9862.5 (6817.7-13421.9) | 6346.6 (5160.2-8045.9) | 6599.2 (5365.5-8366.1) | -1.494 (-1.584--1.404) |
| Cook Islands | 54.5 (44.7-64.1) | 9262 (7605.5-10901.3) | 78.1 (63.7-95.2) | 5015.8 (4093.5-6113) | -2.29 (-2.497--2.083) |
| Costa Rica | 4886 (4448.4-5211.8) | 5158.2 (4696.3-5502.1) | 13934.2 (11896.5-15428.2) | 4443.3 (3793.5-4919.7) | -0.718 (-1.184--0.249) |
| Cote d'Ivoire | 8695.5 (7163.1-10620.7) | 6498.7 (5353.5-7937.5) | 19595.4 (16093.6-24574.6) | 4699.6 (3859.7-5893.7) | -0.888 (-1.030--0.746) |
| Croatia | 11258.5 (10306.6-12206.9) | 3972.1 (3636.2-4306.6) | 27065.2 (24116.2-30183.1) | 4402.1 (3922.4-4909.2) | 0.788 (0.608-0.968) |
| Cuba | 18509.6 (17224.7-19926.5) | 2958 (2752.6-3184.4) | 54145.3 (47870.9-60474.2) | 4360.4 (3855.1-4870.1) | 1.295 (1.107-1.482) |
| Cyprus | 3899.4 (2818.4-4706.1) | 7561.2 (5465.1-9125.5) | 6243.9 (5300.7-7251) | 4612 (3915.3-5355.9) | -1.801 (-1.982--1.619) |
| Czech Republic | 29412.4 (27282.8-31937) | 3690.4 (3423.2-4007.2) | 56093.6 (50204.4-62332.4) | 3632.1 (3250.8-4036.1) | 1.29 (0.741-1.842) |
| Democratic Republic of the Congo | 50768.5 (36892.4-69841.1) | 8971.7 (6519.6-12342.2) | 111289.8 (73616.4-177389.2) | 8801.5 (5822-14029) | -0.076 (-0.117--0.035) |
| Denmark | 36347.4 (33580.4-38677.1) | 6502.3 (6007.3-6919) | 61702.3 (54788.2-66967.4) | 7177.3 (6373.1-7789.8) | -0.008 (-0.354-0.339) |
| Djibouti | 245 (178.5-323.9) | 5764.1 (4199.8-7621) | 887.9 (619.2-1215) | 4054.3 (2827.2-5547.5) | -1.275 (-1.449--1.101) |
| Dominica | 129 (111.6-147.1) | 3345.9 (2894.3-3815.1) | 156.5 (134.1-186) | 3467.5 (2972.6-4122.2) | -0.03 (-0.099-0.039) |
| Dominican Republic | 4619.4 (3949.8-5408.4) | 2505.3 (2142.2-2933.2) | 14426.6 (10994.1-20729.4) | 2599.8 (1981.2-3735.6) | 0.457 (0.197-0.717) |
| Ecuador | 13405.5 (12419.6-14255.7) | 4950.1 (4586.1-5264.1) | 30236.3 (25790.8-35383.7) | 3234.2 (2758.7-3784.8) | -0.765 (-1.008--0.522) |
| Egypt | 77360.2 (62357.6-89961.4) | 7870.4 (6344.1-9152.4) | 113677.7 (96034.3-135359.5) | 5007.3 (4230.1-5962.3) | -1.748 (-1.928--1.568) |
| El Salvador | 7562.9 (6635.1-8584.5) | 4773.3 (4187.7-5418) | 15290 (12343-18743.2) | 3992.9 (3223.3-4894.7) | -0.533 (-0.676--0.390) |
| Equatorial Guinea | 744.1 (522.6-995.6) | 9916.3 (6964.6-13267.8) | 983.8 (738.6-1339.2) | 5138.9 (3857.7-6995.3) | -2.411 (-2.689--2.132) |
| Eritrea | 2389 (1543.8-3143.8) | 8482.8 (5481.7-11163) | 6019.4 (4794.3-7497.3) | 6556.9 (5222.4-8166.8) | -0.87 (-0.963--0.776) |
| Estonia | 2048 (1887.3-2215.5) | 1726.1 (1590.6-1867.2) | 3010.5 (2654.5-3387.2) | 1573.3 (1387.3-1770.2) | -0.103 (-0.283-0.076) |
| Ethiopia | 63253.3 (46043-77241.4) | 8184.4 (5957.6-9994.3) | 102261.7 (84844.5-117429.5) | 5186.8 (4303.4-5956.1) | -1.776 (-1.912--1.640) |
| Federated States of Micronesia | 423.2 (311.2-531) | 18016.6 (13247.4-22606.9) | 270.2 (212.9-340.1) | 10695.9 (8428.3-13464) | -1.704 (-1.793--1.615) |
| Fiji | 1353.8 (1121-1617.5) | 10011.3 (8289.8-11961.4) | 2038.8 (1604.8-2542.2) | 6336.8 (4988-7901.5) | -2.076 (-2.360--1.790) |
| Finland | 12582 (11673.2-13609.6) | 2751 (2552.3-2975.7) | 24346.5 (21724.6-26720.9) | 2598.7 (2318.9-2852.2) | -0.271 (-0.451--0.090) |
| France | 203585.8 (184757.8-217137.3) | 3912.7 (3550.8-4173.1) | 226172.5 (195937.5-248931) | 2277.1 (1972.7-2506.2) | -1.955 (-2.420--1.487) |
| Gabon | 2271.6 (1790.5-2846.5) | 8049 (6344.3-10085.7) | 2093.1 (1630-2892) | 5165.7 (4022.7-7137.3) | -1.499 (-1.615--1.383) |
| Georgia | 7014 (6208.9-7854) | 2124.7 (1880.9-2379.2) | 9584.3 (8439.7-10743.6) | 2569.5 (2262.6-2880.3) | 2.424 (1.902-2.948) |
| Germany | 382051.8 (351046.9-409040.5) | 4725.8 (4342.3-5059.6) | 546515.9 (484238.5-593753.5) | 4031.7 (3572.3-4380.2) | -0.295 (-0.553--0.036) |
| Ghana | 7526.2 (6164.8-9058.6) | 2970 (2432.8-3574.8) | 21037 (16988.7-25488.1) | 3055.9 (2467.8-3702.5) | 0.494 (0.251-0.738) |
| Greece | 33652.9 (30616.2-36993.4) | 3552.4 (3231.9-3905.1) | 77008.3 (67579.9-85197.2) | 4448.1 (3903.5-4921.1) | 1.189 (0.689-1.691) |
| Greenland | 208.5 (168-240.7) | 16802.6 (13540.5-19393.1) | 253.2 (206.2-310.6) | 8420.2 (6856.2-10326.3) | -2.179 (-2.370--1.987) |
| Grenada | 110.2 (99.8-121.4) | 2215 (2005.7-2439.4) | 164.8 (145.4-183) | 2788.1 (2460.4-3096.1) | 0.199 (-0.555-0.959) |
| Guam | 198.6 (175.9-221.3) | 7133.1 (6316.2-7949.4) | 375 (322.3-431) | 3064.7 (2633.7-3522.1) | -2.578 (-2.823--2.332) |
| Guatemala | 6673.9 (6069.8-7268.7) | 4548.7 (4136.9-4954.1) | 20149.2 (17585.7-22788) | 3418.2 (2983.3-3865.9) | -0.861 (-1.286--0.435) |
| Guinea | 10485.6 (7490-13382.6) | 6323.7 (4517.1-8070.8) | 14539.2 (11116.6-18261.6) | 5602.4 (4283.6-7036.7) | 0.021 (-0.173-0.215) |
| Guinea-Bissau | 1451.1 (1071.1-1842.3) | 8872.1 (6548.7-11264.5) | 1610 (1176.7-2038.4) | 6342.5 (4635.3-8029.9) | -0.76 (-1.006--0.513) |
| Guyana | 397.3 (356-437.1) | 2264.2 (2028.5-2490.7) | 716.9 (587.3-861.8) | 2477.6 (2029.9-2978.3) | 0.334 (0.132-0.535) |
| Haiti | 8746.6 (4174.9-12600.4) | 6614 (3157-9528.1) | 17278.6 (8607.1-25025.2) | 6079.5 (3028.4-8805.1) | -0.16 (-0.218--0.102) |
| Honduras | 7232 (5954.4-9123.9) | 7411.9 (6102.5-9350.8) | 30174.8 (24117.9-37129.1) | 9575.1 (7653.1-11781.9) | 1.071 (0.852-1.292) |
| Hungary | 51531.6 (47927.8-55333.3) | 6078.4 (5653.3-6526.8) | 68392.9 (60382.9-76342.3) | 5078.3 (4483.5-5668.5) | -0.07 (-0.509-0.370) |
| Iceland | 869.5 (788.6-942.6) | 4758.8 (4316.1-5158.8) | 1667.6 (1439.4-1863.4) | 4407.7 (3804.5-4925.2) | -0.098 (-0.318-0.121) |
| India | 3909844.8 (3162248.8-4617906.6) | 21707.2 (17556.6-25638.3) | 12300925.9 (10911080.1-13697906) | 20644.7 (18312.1-22989.3) | -0.013 (-0.178-0.153) |
| Indonesia | 365230.3 (274657.7-421327.5) | 9591 (7212.5-11064.1) | 863264.1 (723984.4-997292.6) | 8949.5 (7505.6-10339) | -0.222 (-0.338--0.106) |
| Iran | 34975.4 (29424.1-39434.4) | 3604.6 (3032.5-4064.1) | 140030.2 (122555.9-153395.8) | 3834.7 (3356.2-4200.7) | 0.677 (0.507-0.847) |
| Iraq | 10449.4 (8481.4-12918.7) | 2615.8 (2123.1-3233.9) | 25979.3 (20747.9-31141.1) | 2619.4 (2091.9-3139.8) | -0.284 (-0.447--0.120) |
| Ireland | 26261.4 (24610.3-27811.7) | 9773.3 (9158.8-10350.2) | 26824.1 (23320.1-29559) | 5160 (4485.9-5686.1) | -2.232 (-2.501--1.963) |
| Israel | 14097.2 (12954.2-15021.4) | 4637 (4261-4941) | 23890.9 (20739-26316.5) | 2893.7 (2511.9-3187.5) | -1.138 (-1.359--0.917) |
| Italy | 265742.3 (243402.1-281468.5) | 4797.6 (4394.2-5081.5) | 391322.1 (333405.6-428388.2) | 3721 (3170.3-4073.5) | -0.652 (-0.818--0.485) |
| Jamaica | 2780 (2546.5-2988.9) | 2389.6 (2189-2569.2) | 5579.3 (4559.5-6793.4) | 3067.6 (2506.9-3735.1) | 0.672 (0.163-1.183) |
| Japan | 293658.1 (267218.3-317625.3) | 2973.1 (2705.4-3215.7) | 573660.9 (494774.8-639771.4) | 1984.8 (1711.9-2213.6) | -1.258 (-1.459--1.056) |
| Jordan | 2058.1 (1706.7-2469.9) | 4196.3 (3479.8-5036) | 7766.5 (6500.9-9145.5) | 2528.9 (2116.8-2977.9) | -2.113 (-2.353--1.873) |
| Kazakhstan | 46243.2 (41430.4-51154.1) | 7707.6 (6905.4-8526.1) | 67740 (58897.6-77765.3) | 8329.1 (7241.8-9561.7) | 0.187 (-0.430-0.809) |
| Kenya | 22675 (14405-35024) | 6119.5 (3887.6-9452.2) | 60330.6 (39024.5-103616.1) | 6465.6 (4182.2-11104.5) | 0.363 (0.264-0.463) |
| Kiribati | 235 (152.1-353.8) | 16299.4 (10544-24533.4) | 321.8 (240.3-495.3) | 12932.2 (9656.7-19903) | -0.835 (-0.910--0.761) |
| Kuwait | 331.2 (293.3-369) | 1658.7 (1469-1848.1) | 1442.7 (1221.6-1687.6) | 1461.3 (1237.3-1709.3) | -0.222 (-0.454-0.011) |
| Kyrgyzstan | 22131.5 (20300.2-23924.9) | 15304.1 (14037.7-16544.2) | 10488.1 (9016.6-11987) | 5464.2 (4697.6-6245.2) | -3.628 (-4.120--3.134) |
| Laos | 13355.4 (9339.4-16960) | 15050 (10524.4-19112.1) | 20342.3 (15699-25767.4) | 10560.3 (8149.8-13376.6) | -1.348 (-1.440--1.257) |
| Latvia | 5126.7 (4715.2-5601.8) | 2471 (2272.6-2700) | 4487.3 (3947.7-5112) | 1593.6 (1402-1815.4) | -1.209 (-1.593--0.822) |
| Lebanon | 5667.4 (3879.9-7323) | 5926.1 (4057-7657.3) | 18835.3 (16178-22012) | 4898.2 (4207.1-5724.3) | -0.22 (-0.405--0.034) |
| Lesotho | 5177.3 (3944-6738.1) | 12069.2 (9194.2-15708) | 6376.1 (4777.1-8051.9) | 13262.1 (9936.1-16747.7) | 0.598 (0.391-0.806) |
| Liberia | 3652.8 (2887.2-4388.2) | 6139.3 (4852.6-7375.4) | 4105.1 (3146.4-5326.4) | 5147.5 (3945.3-6679) | -0.484 (-0.637--0.329) |
| Libya | 3392.3 (2611.4-4337.7) | 3903.3 (3004.8-4991.2) | 9032.7 (7138.7-11393.1) | 4121.8 (3257.6-5198.9) | 0.597 (0.423-0.771) |
| Lithuania | 13112.9 (12248.5-13870.3) | 5166.5 (4826-5464.9) | 7949.8 (7060.3-8744.8) | 1979.3 (1757.9-2177.3) | -3.253 (-3.390--3.117) |
| Luxembourg | 1855.8 (1721.8-1977) | 5434.2 (5041.9-5789.3) | 2969.3 (2625.3-3291.2) | 4467.7 (3950.1-4952) | -0.566 (-0.757--0.375) |
| Macedonia | 5048.6 (4437.8-5814.6) | 5728.7 (5035.5-6597.8) | 7536.6 (5951.6-9837.6) | 3966.8 (3132.5-5177.9) | -1.261 (-1.372--1.149) |
| Madagascar | 22974.8 (18389-27614.8) | 10702.3 (8566.2-12863.8) | 34879.9 (26404.8-44403) | 9589.5 (7259.4-12207.7) | -0.495 (-0.565--0.424) |
| Malawi | 9175.6 (7446.2-11272.6) | 5899.6 (4787.6-7247.9) | 17346.8 (14181.3-21019) | 5576.1 (4558.6-6756.6) | -0.392 (-0.547--0.236) |
| Malaysia | 37028.9 (31730.8-42836.8) | 8509.5 (7292-9844.2) | 80987.7 (70123.8-92219.5) | 5727.2 (4959-6521.5) | -2.031 (-2.341--1.719) |
| Maldives | 459.1 (320.5-554.7) | 15302.2 (10683-18488.2) | 1070.8 (915.1-1272.9) | 7872.7 (6728.2-9359) | -2.587 (-2.804--2.369) |
| Mali | 13825.9 (11311.4-16504.9) | 8508.6 (6961.2-10157.3) | 28413.3 (22616.2-35921) | 7696.2 (6126-9729.8) | -0.043 (-0.194-0.108) |
| Malta | 1167.9 (1072.9-1261.4) | 4704 (4321.2-5080.6) | 1774.2 (1573.5-1996.6) | 2471.4 (2191.7-2781.1) | -2.157 (-2.351--1.962) |
| Marshall Islands | 114.9 (86.9-141.8) | 15650.7 (11841.6-19312.9) | 121.3 (90.1-157.5) | 10562.8 (7849.3-13721.5) | -1.175 (-1.255--1.094) |
| Mauritania | 3072.1 (2381.6-3734.9) | 5887.8 (4564.6-7158.2) | 3992 (3188.9-4916.5) | 3921.6 (3132.6-4829.7) | -1.168 (-1.510--0.825) |
| Mauritius | 2096.4 (1942.3-2251.2) | 6366.1 (5898.3-6836.3) | 3698.4 (3381.4-3979.7) | 3688.1 (3372-3968.6) | -1.654 (-1.881--1.426) |
| Mexico | 152992.9 (145947.8-158174.3) | 7366.8 (7027.6-7616.3) | 370085.9 (334100.8-405868.2) | 5477.3 (4944.7-6006.8) | -0.918 (-1.037--0.799) |
| Moldova | 15304 (14427.3-16227) | 7286 (6868.6-7725.4) | 8491.8 (7628.8-9379.4) | 2436.8 (2189.2-2691.5) | -4.01 (-4.544--3.474) |
| Monaco | 167.6 (136.8-199.5) | 3382.3 (2760.6-4027.5) | 219.2 (181.7-258.3) | 3079.3 (2552.3-3628.2) | -0.115 (-0.259-0.030) |
| Mongolia | 3501.5 (2858.5-4272.2) | 6814 (5562.6-8313.7) | 3389.9 (2804.9-4006.7) | 4029 (3333.7-4762.2) | -2.498 (-2.785--2.209) |
| Montenegro | 493.3 (426.2-578.7) | 1541.4 (1331.8-1808.1) | 939.8 (785.5-1117.5) | 1615.5 (1350.3-1920.9) | 0.352 (0.163-0.541) |
| Morocco | 28787.6 (20389.9-36496.6) | 4058.2 (2874.4-5145) | 78237 (64210.1-94749.9) | 4734.3 (3885.5-5733.5) | 0.68 (0.554-0.806) |
| Mozambique | 12365.8 (9665.9-15225) | 5088.1 (3977.2-6264.6) | 21845 (17310.1-26832.3) | 5081.6 (4026.7-6241.8) | 0.19 (0.060-0.321) |
| Myanmar | 223887.2 (171381-278385.2) | 22890.8 (17522.4-28462.8) | 422409.5 (329784.4-511305.1) | 18902.3 (14757.5-22880.3) | -0.773 (-0.848--0.698) |
| Namibia | 3304.4 (2513.1-4201.8) | 12443.9 (9464.2-15823.6) | 6162.7 (5033.1-7763) | 10294.2 (8407.2-12967.3) | -0.685 (-0.817--0.553) |
| Nauru | 25.7 (19.6-40.3) | 16289.9 (12408.2-25563.7) | 27.4 (19.2-51.1) | 12126.5 (8480.4-22603.4) | -0.915 (-1.121--0.708) |
| Nepal | 111401.4 (80978.7-137865.5) | 30552.6 (22208.9-37810.5) | 304368.2 (246225.4-374251.3) | 26125.5 (21134.8-32123.9) | -0.351 (-0.548--0.154) |
| Netherlands | 86684.9 (80132.9-91542.6) | 6757.4 (6246.6-7136.1) | 138132.6 (121541-150251.3) | 5624.4 (4948.8-6117.8) | -0.695 (-0.855--0.534) |
| New Zealand | 15936.2 (14813.9-16847.7) | 6440.7 (5987.1-6809.1) | 25197.3 (22040-27351.2) | 4503.8 (3939.5-4888.8) | -1.396 (-1.606--1.186) |
| Nicaragua | 2568.5 (2252.4-2914.4) | 3490 (3060.5-3960) | 9576.6 (7969.9-11157.3) | 3835.5 (3192-4468.6) | 0.799 (0.385-1.215) |
| Niger | 6940 (4791.8-9022.8) | 6750.7 (4661-8776.6) | 18414.2 (13468.3-23682.2) | 5404.4 (3952.8-6950.5) | -0.373 (-0.600--0.146) |
| Nigeria | 99583.6 (80457-119282.8) | 4753 (3840.2-5693.3) | 152433.8 (131577.6-175381.9) | 4071.1 (3514.1-4684) | -0.449 (-0.526--0.372) |
| Niue | 20.3 (17.3-23.6) | 13839.4 (11803.1-16098.5) | 10.6 (8.8-12.8) | 8947.5 (7409.9-10775.5) | -1.587 (-1.689--1.486) |
| North Korea | 181973 (132064-248080.9) | 27792.3 (20169.8-37888.8) | 346423.3 (261904-473751.4) | 19794.8 (14965.3-27070.3) | -1.114 (-1.164--1.065) |
| Northern Mariana Islands | 51.6 (44.1-61.9) | 10729.3 (9172.2-12876.7) | 165.6 (140.8-204.7) | 7819.9 (6648-9664.6) | -0.889 (-0.991--0.786) |
| Norway | 14103.9 (13106.4-15130) | 2886.6 (2682.5-3096.7) | 38279.5 (34182.4-41131.1) | 5529.5 (4937.6-5941.4) | 2.148 (1.676-2.622) |
| Oman | 1093.9 (834.3-1361.9) | 3790.5 (2891-4719.1) | 2337.2 (1987-2793.7) | 3385 (2877.8-4046.1) | 0.034 (-0.240-0.310) |
| Pakistan | 452792.3 (364740.6-524723.2) | 16016.6 (12901.9-18561) | 732162.2 (605166-909939.3) | 14459 (11951-17969.8) | -0.551 (-0.815--0.286) |
| Palau | 61.3 (50.6-74.4) | 12826 (10596.1-15574.6) | 92.9 (74.5-113.3) | 10190.4 (8168.6-12426.7) | -0.465 (-0.628--0.301) |
| Palestine | 2248.5 (1745.8-2742.1) | 5331.5 (4139.5-6501.9) | 3592.6 (3135.6-4156.2) | 3460.7 (3020.4-4003.6) | -1.488 (-1.685--1.290) |
| Panama | 2925.7 (2681.2-3142.6) | 3625.6 (3322.6-3894.4) | 9433.4 (7658.6-10997.8) | 3614.7 (2934.6-4214.1) | -0.386 (-0.745--0.025) |
| Papua New Guinea | 17938.3 (13461.4-23250.3) | 29877.8 (22421-38725.2) | 44720.5 (35608.3-55344.9) | 25697.1 (20461.1-31802) | -0.422 (-0.473--0.372) |
| Paraguay | 3895.7 (3349.8-4468.7) | 3359.6 (2888.8-3853.7) | 11515.2 (9396.9-14121.3) | 3814.4 (3112.7-4677.6) | 0.779 (0.645-0.912) |
| Peru | 15746.7 (12036.3-19345.5) | 2626.5 (2007.6-3226.8) | 42583.5 (31847-54907) | 2261.9 (1691.6-2916.4) | -0.206 (-0.361--0.051) |
| Philippines | 105841.2 (96233.4-116696.6) | 8167 (7425.6-9004.6) | 244643.6 (214191.2-279037.8) | 6762.9 (5921-7713.6) | -0.668 (-0.743--0.592) |
| Poland | 118097.6 (112294.5-123814) | 4907.8 (4666.6-5145.4) | 132998.5 (121238.1-145753) | 2847.5 (2595.7-3120.6) | -1.488 (-1.616--1.360) |
| Portugal | 45763.1 (42744.8-48805.8) | 5323.7 (4972.6-5677.7) | 73957.4 (64791.3-80664.5) | 4179.4 (3661.4-4558.5) | -0.917 (-1.138--0.696) |
| Puerto Rico | 8963.4 (8347.1-9592.1) | 4010.2 (3734.5-4291.5) | 19264.7 (16188.9-22162.6) | 3657.6 (3073.6-4207.8) | -1.158 (-1.527--0.787) |
| Qatar | 142.7 (119.3-173.7) | 5914.2 (4942-7198.1) | 556.3 (459.4-649.1) | 3167.7 (2615.7-3696.1) | -2.116 (-2.617--1.612) |
| Romania | 135554.7 (126860.9-143845.9) | 9499.6 (8890.3-10080.6) | 86115.9 (77609.3-95267.5) | 3401.9 (3065.8-3763.4) | -3.118 (-3.445--2.791) |
| Russian Federation | 502308 (477213.3-522712.4) | 5202.1 (4942.3-5413.5) | 380733.3 (349290.2-412341.8) | 2661.1 (2441.3-2882) | -2.398 (-2.672--2.123) |
| Rwanda | 12213 (9003-14849.2) | 11060.4 (8153.4-13447.9) | 16361.7 (12235.1-21665.8) | 6621.7 (4951.6-8768.3) | -2.397 (-2.725--2.068) |
| Saint Kitts and Nevis | 70.5 (64.5-75.9) | 2609 (2387-2807.4) | 88.5 (77-98.7) | 3186.2 (2770.7-3552.4) | 0.734 (0.512-0.957) |
| Saint Lucia | 220.8 (206.6-239.7) | 4225 (3951.9-4586.5) | 569.9 (476.4-664) | 4195.6 (3507.3-4888.7) | -0.626 (-0.922--0.329) |
| Saint Vincent and the Grenadines | 60.3 (54.5-66) | 1381.9 (1249-1512) | 170.8 (152.8-190.4) | 2147 (1920.4-2392.6) | 1.268 (1.006-1.531) |
| Samoa | 653.2 (506.6-840.3) | 17237.8 (13369.3-22175.3) | 823.7 (659.4-1001.1) | 12056.2 (9650.7-14652.1) | -1.094 (-1.176--1.013) |
| San Marino | 72.4 (61.5-83.6) | 3098.5 (2635.2-3580.5) | 107.9 (85-132) | 2070.4 (1631.8-2532.8) | -0.554 (-0.844--0.264) |
| Sao Tome and Principe | 373.1 (305.3-441.4) | 11292.4 (9240.7-13360.1) | 493.6 (385.9-601.7) | 10766.5 (8417.1-13125.7) | -0.04 (-0.156-0.076) |
| Saudi Arabia | 15063.2 (11362.5-18797.7) | 6067.8 (4577.1-7572.2) | 20817.7 (17439.4-24944.7) | 4011.3 (3360.4-4806.6) | -1.48 (-1.545--1.413) |
| Senegal | 9350.5 (7583.4-11341.2) | 6183.2 (5014.6-7499.5) | 17860.8 (14211.9-22004.4) | 5044.4 (4013.9-6214.7) | -0.344 (-0.729-0.042) |
| Serbia | 28933.1 (24750.3-34426.6) | 5825.5 (4983.3-6931.6) | 46411.8 (39560.4-53519.1) | 4292.7 (3659-4950.1) | -0.871 (-1.010--0.732) |
| Seychelles | 225.7 (195.7-251.8) | 6595.6 (5719.5-7359.4) | 278.8 (241.7-316.5) | 5049 (4376.8-5730.6) | -1.003 (-1.161--0.844) |
| Sierra Leone | 6795.2 (5391.9-8214.4) | 6290 (4991.1-7603.7) | 8458.6 (6364.4-10984.3) | 4933.1 (3711.7-6406.1) | -0.559 (-0.699--0.420) |
| Singapore | 8522.7 (8033.6-8940.2) | 8142.4 (7675.2-8541.4) | 7318.9 (6408.1-8067.7) | 1514.7 (1326.2-1669.7) | -5.454 (-5.606--5.301) |
| Slovakia | 8941.2 (7750.9-10070.5) | 2704.7 (2344.6-3046.4) | 13558.1 (11699.1-15902) | 2255.1 (1945.9-2645) | -0.213 (-0.359--0.067) |
| Slovenia | 7487.5 (6944-7994.8) | 5467.6 (5070.7-5838) | 8979.5 (7885.3-9922.2) | 2988.4 (2624.3-3302.2) | -2.396 (-2.712--2.079) |
| Solomon Islands | 657.2 (521.9-806.5) | 13999.6 (11116.4-17180.4) | 1432.5 (1177.3-1747.5) | 10809 (8883.6-13186) | -0.826 (-0.899--0.752) |
| Somalia | 7309 (4666.1-9774.1) | 10415 (6649.1-13927.8) | 14938.2 (9501.8-20439.3) | 7840.8 (4987.3-10728.2) | -0.913 (-1.013--0.813) |
| South Africa | 72966.9 (63906.9-89143.1) | 7191.8 (6298.9-8786.2) | 155337.9 (141806.6-169481) | 7021 (6409.4-7660.3) | -0.317 (-0.668-0.036) |
| South Korea | 70742.2 (58774.8-81947) | 5573 (4630.2-6455.7) | 180001.6 (153388-211507.8) | 3249 (2768.6-3817.7) | -1.873 (-2.016--1.729) |
| South Sudan | 10790.3 (7330-14295.8) | 8586.4 (5832.9-11376) | 9732.1 (6652.3-13466.4) | 6991.5 (4779-9674.2) | -0.898 (-1.070--0.725) |
| Spain | 239572.8 (222017.4-254237.8) | 6950.9 (6441.5-7376.4) | 364383.4 (317192.6-398964.4) | 5385 (4687.6-5896) | -0.903 (-1.059--0.747) |
| Sri Lanka | 65592 (57035.9-73940.9) | 13300.9 (11565.8-14993.8) | 132006 (94575.6-169525.7) | 8676.1 (6216-11142) | -0.884 (-1.182--0.584) |
| Sudan | 27904.1 (14315.3-43125.2) | 5920.9 (3037.5-9150.5) | 42642.1 (30516.6-57836.8) | 4980.5 (3564.3-6755.2) | -0.666 (-0.741--0.591) |
| Suriname | 452.3 (394.1-511.7) | 3703.3 (3226.1-4189.5) | 929.8 (721.5-1200.1) | 2794.4 (2168.6-3607) | -0.608 (-0.801--0.414) |
| Swaziland | 1534.8 (1068.8-1930.1) | 12981.4 (9040-16324.7) | 2263.3 (1695.1-2984.5) | 10066.8 (7539.6-13274.7) | -0.602 (-0.939--0.264) |
| Sweden | 31303.9 (28574.8-34666.5) | 2867.9 (2617.8-3175.9) | 59730.2 (52401.2-66224.3) | 3745.3 (3285.7-4152.5) | 0.921 (0.568-1.276) |
| Switzerland | 29572.9 (27099.8-32162.1) | 4239.6 (3885.1-4610.8) | 38356.8 (33451.6-42629.9) | 3074.4 (2681.2-3416.9) | -0.996 (-1.073--0.919) |
| Syria | 10502 (8095-13032.8) | 4713.9 (3633.5-5849.9) | 27295.1 (22336-33523.6) | 4622.3 (3782.5-5677) | -0.227 (-0.383--0.072) |
| Taiwan | 42698.1 (39776.1-45855.8) | 5854.6 (5454-6287.6) | 105773.7 (93892.7-116041.2) | 4188.2 (3717.8-4594.8) | -1.055 (-1.219--0.891) |
| Tajikistan | 10726.3 (8652.2-13210.5) | 8375.3 (6755.8-10315) | 11062.1 (8264.5-14339.4) | 5560.3 (4154.1-7207.6) | -1.456 (-1.718--1.193) |
| Tanzania | 26482.4 (21090.5-31430) | 5435.8 (4329-6451.4) | 48866.8 (39282.3-60679.1) | 4399.7 (3536.8-5463.2) | -0.923 (-1.012--0.835) |
| Thailand | 159792.1 (133147-186431.9) | 10453.6 (8710.5-12196.4) | 276104.9 (220636.8-336703.1) | 4431.8 (3541.4-5404.4) | -3.402 (-3.605--3.199) |
| The Bahamas | 135.9 (123.6-148.1) | 1681.5 (1529.1-1831.4) | 391.9 (332.8-457.4) | 2022.2 (1716.9-2359.9) | 0.402 (0.280-0.523) |
| The Gambia | 997 (767.6-1245.1) | 6461.6 (4974.9-8070.1) | 2604.9 (1934.3-3378) | 5810.9 (4315-7535.6) | -0.194 (-0.460-0.073) |
| Timor-Leste | 960.5 (659.1-1206.5) | 11257.5 (7724.7-14139.8) | 4231.6 (3208.4-5398.2) | 9357.9 (7095.1-11937.6) | -0.48 (-0.622--0.338) |
| Togo | 3230.4 (2556.6-3900.8) | 6371.9 (5043-7694.5) | 7488.8 (5759.7-9915.4) | 5181.5 (3985.2-6860.5) | -0.516 (-0.676--0.355) |
| Tokelau | 8.9 (6.6-11.6) | 11881 (8781.6-15434.3) | 7.8 (5.8-11.4) | 8505.9 (6352.7-12392.8) | -1.06 (-1.118--1.002) |
| Tonga | 301.8 (247.2-352.1) | 11618.5 (9517.7-13558.1) | 412.4 (339.1-478.5) | 9412.6 (7740.6-10921.1) | -0.672 (-0.827--0.518) |
| Trinidad and Tobago | 1235.3 (1152.1-1311) | 2616.8 (2440.6-2777) | 2350.1 (1906-2848.5) | 2100.3 (1703.4-2545.7) | -0.958 (-1.129--0.786) |
| Tunisia | 9077.9 (7555.1-10821.7) | 3755 (3125.1-4476.3) | 28158.8 (21730.8-37153.3) | 4044.3 (3121.1-5336.2) | 0.249 (0.189-0.309) |
| Turkey | 163804.9 (130264.4-190969.6) | 10920.2 (8684.2-12731.1) | 418413.1 (356197.9-491184.1) | 8230.2 (7006.4-9661.6) | -0.405 (-0.750--0.058) |
| Turkmenistan | 5615.6 (5193.6-6033.4) | 6632.7 (6134.3-7126.2) | 3370.1 (2871.8-3950.1) | 2151.4 (1833.3-2521.7) | -4.316 (-5.031--3.596) |
| Tuvalu | 40.6 (29.8-50.5) | 15030.5 (11048.9-18691.7) | 45.4 (34.8-57.8) | 8919.1 (6827.8-11348.9) | -1.713 (-1.747--1.679) |
| Uganda | 24178.8 (17017.4-31483) | 8261.1 (5814.3-10756.7) | 34262.1 (24564.1-44196.5) | 5694.1 (4082.4-7345.1) | -1.59 (-1.725--1.454) |
| Ukraine | 337327.1 (315943.8-355960.3) | 8415.5 (7882-8880.4) | 98167 (81841.4-116962.1) | 2013 (1678.2-2398.4) | -5.596 (-5.905--5.285) |
| United Arab Emirates | 790.2 (598.5-1016.7) | 6493 (4917.8-8354.9) | 2669.3 (2117.4-3327.5) | 5529.1 (4385.8-6892.5) | 1.522 (0.831-2.219) |
| United Kingdom | 425684.2 (403299-443263.4) | 6841 (6481.3-7123.5) | 539528.6 (490332.3-573320.7) | 5897.1 (5359.4-6266.5) | -0.336 (-0.494--0.177) |
| United States | 1301177.5 (1200637.3-1385311.2) | 6135 (5661-6531.7) | 2776564 (2496468-2965750) | 7214.5 (6486.7-7706.1) | 0.563 (0.296-0.831) |
| Uruguay | 12834.2 (11984.8-13569.4) | 5228.5 (4882.5-5528) | 22388.6 (20359.2-23872.3) | 5958.1 (5418-6352.9) | 0.28 (0.079-0.481) |
| Uzbekistan | 26411.2 (23061.6-31134.3) | 4630.7 (4043.4-5458.8) | 20374.1 (17836.3-23142) | 2136.8 (1870.6-2427.1) | -3.25 (-3.935--2.559) |
| Vanuatu | 405.9 (268.7-526.2) | 17634.7 (11675-22861.2) | 861.1 (573.9-1092.9) | 12935.3 (8620-16416.6) | -1.177 (-1.257--1.097) |
| Venezuela | 17128.9 (15625.8-18364.2) | 3608.2 (3291.6-3868.5) | 71914.2 (58060.2-87634.4) | 4478 (3615.4-5456.9) | 0.597 (0.281-0.914) |
| Vietnam | 204329 (156119.5-251536.7) | 10020.9 (7656.6-12336.2) | 389162.1 (317225.8-466827.5) | 8710.6 (7100.4-10448.9) | -0.309 (-0.396--0.222) |
| Virgin Islands, U.S. | 92.3 (77.2-107.9) | 2304.5 (1927.3-2692.2) | 201.4 (161.6-250.1) | 1561.5 (1253.2-1939.1) | -1.559 (-1.830--1.287) |
| Yemen | 12739.9 (7580.6-17426.4) | 6530.5 (3885.9-8932.9) | 34395.1 (26011.3-43971.9) | 5782.2 (4372.7-7392.1) | -0.385 (-0.456--0.314) |
| Zambia | 7432.8 (5741.3-9079.5) | 6416.6 (4956.4-7838.2) | 15361.1 (12535.5-18676.4) | 5708.7 (4658.6-6940.7) | -0.626 (-0.754--0.499) |
| Zimbabwe | 10388.7 (8389.1-12497.1) | 5792.8 (4677.8-6968.5) | 16499 (13230.1-20058.8) | 6215.3 (4983.9-7556.3) | 0.467 (0.238-0.696) |

EAPC, estimated annual percentage change; UI, uncertainty interval; CI, confidence interval.
