# Supplementary material for: Burden of chronic obstructive pulmonary disease in adults aged 70 years and older, 1990–2021: Findings from the Global Burden of Disease Study 2021
Source: PLoS One. 2025 Jan 8;20(1):e0316135. doi: 10.1371/journal.pone.0316135 (PMC11709235; doi:10.1371/journal.pone.0316135)
Supplement: Attachment — Submitted filename: Response to Reviewers.docx [file pone.0316135.s002.docx]

Dear editor and reviewers:

Thank you very much for all the nice and insightful comments, which dramatically improved the manuscript quality. We have responded to all the comments point-by-point as shown below and have made revisions in the manuscript accordingly. The revisions in the lines of the revised manuscript have been provided.

**Responses to journal requirements:**

1. When submitting your revision, we need you to address these additional requirements.

Please ensure that your manuscript meets PLOS ONE's style requirements, including those for file naming.

**Reply:** Thanks for your comments. Thanks for your comments. I will make sure the manuscript meets PLOS ONE's style requirements, including those for file naming.

2. Thank you for stating in your Funding Statement: 
This study was supported by the Scientific Research Project of Fuyang City, Anhui Province (FY2020xg01).

Please provide an amended statement that declares *all* the funding or sources of support (whether external or internal to your organization) received during this study, Please also include the statement “There was no additional external funding received for this study.” in your updated Funding Statement. 
Please include your amended Funding Statement within your cover letter. We will change the online submission form on your behalf.

**Reply:** Thanks for your comments. We have included the amended Funding Statement within our cover letter as follows：

This study was supported by the Scientific Research Project of Fuyang City, Anhui Province (FY2020xg01). There was no additional external funding received for this study.

3. PLOS requires an ORCID iD for the corresponding author in Editorial Manager on papers submitted after December 6th, 2016.

**Reply:** Thanks for your comments. I appreciate the reminder regarding the ORCID iD requirement for the corresponding author. I will ensure that the ORCID iD is added to the submission in Editorial Manager as per PLOS guidelines.

4. We note that Figures 4 and 5 in your submission contain map images which may be copyrighted. All PLOS content is published under the Creative Commons Attribution License (CC BY 4.0), which means that the manuscript, images, and Supporting Information files will be freely available online, and any third party is permitted to access, download, copy, distribute, and use these materials in any way, even commercially, with proper attribution. For these reasons, we cannot publish previously copyrighted maps or satellite images created using proprietary data, such as Google software (Google Maps, Street View, and Earth).

**Reply:** Thanks for your thoughtful comments.

Thank you for your questions regarding the map images in Fig 4 and 5. Please find our responses below:

a) Source of Map Images:

The map images in Fig 4 and 5 were created using the rnaturalearth R package, which provides access to Natural Earth map data. This data is open-source and available at https://github.com/ropensci/rnaturalearth.

b) Copyright Status:

To our knowledge, the map data from Natural Earth provided through the rnaturalearth package is in the public domain and therefore not subject to copyright restrictions. Natural Earth map data is widely used for world mapping and is explicitly designed for open, unrestricted use. We believe no additional copyright permission is required.

Please let us know if any further clarification is required, or if additional details or documentation are needed to satisfy PLOS ONE’s requirements.

**Responses to** **Reviewer 1:**

1.Abstract: The method is too brief and more details are required. The authors can check previous GBD studies to get idea for improving this section.

**Reply:** Thanks for the reviewer's comments. We agree with the reviewer's opinion and have expanded description of our methodology in the revised manuscript (lines 34-35).

2. Abstract: There is no mention of risk factors in the aim of the study.

**Reply:** Thanks for your thoughtful consideration. We agree with the reviewer's opinion and have added the description of risk factors to the aim of the study (lines 29-30).

3. The reason for increased prevalence can be attributed to higher life expectancies. Thus, we cannot use it to highlight the problem as the death rate is also decreasing. The authors should mention the trend for incidence rate as well. Also, it is important to discuss the results by its incidence throughout the manuscript.

**Reply:** Thanks a lot for your professional advice. We agree with your opinion, the incidence of COPD among older adults has also increased over the past 3 decades. We have added the description of incidence (S1 and S5 Tables in File 1; and the corresponding part in Fig1-2, Fig4-5; S2, S4-5, S13 Figs) and discussed the results involving incidence throughout the revised manuscript.

4. -line 93: abbreviations needs to be defined

**Reply:** Thanks a lot for your thoughtful consideration. We have added the abbreviations of FEV1, FVC in the revised manuscript (lines 469-470).

5. There was no mention of COVID-19 in this paper, which needs to be discussed in GBD2021 results, especially in respiratory diseases.

**Reply:** Thanks a lot for your professional comment. The COVID-19 pandemic had a substantial impact on COPD globally, which caused a huge disruption to healthcare services, lockdowns, and reduced access to diagnostic and treatment facilities. This may lead to delays in COPD diagnoses and treatments, which in turn resulted in an increase in COPD cases during and after the pandemic. In addition, COVID-19 itself contributed to COPD-related complications, exacerbations, and mortality, either directly or indirectly, which might lead to a temporary rise in the observed burden of elderly COPD. We have added the part of COVID-19 in the discussion in the revised manuscript (lines 348-353; lines 388-391).

6. The authors have not discussed about the policymaking and interventions. The can suggest new ones and also discuss the previous ones that led to reducing the burden of disease.

**Reply:** Thanks a lot for your professional comment. We agree with your opinion and added the policymaking and interventions in the part of the discussion.

(a). In high SDI regions, where the aging population contributes to increased COPD prevalence, policies should focus on geriatric care, chronic disease management, and preventive healthcare. In low SDI regions, policies must focus on strengthening primary healthcare systems and increasing the availability of basic diagnostic tools for COPD, such as spirometers. These regional inequalities highlight the need for a multifaceted approach to COPD management in older adults (lines 373-377).

(b). We acknowledged the impact of widespread tobacco control measures, environmental pollution management, enhanced access to disease-modifying treatments, and management of comorbidities in the general population. And on this basis, we emphasized that ambient ozone pollution remains a challenge for this population, especially in low and low-middle SDI regions (lines 394-398; lines 416-418).

**Responses to Reviewer 2:**

The manuscript is well-written and the overall quality of the introduction and discussion is acceptable. However, some minor modifications are suggested:
1. - The Methods section is too brief. Please provide additional details regarding the models used in the GBD analysis.

**Reply:** Thanks a lot for your professional advice. We agree with your opinion, and we have added the description of DisMod-MR 2.1 (Prevalence and incidence estimates) and CODEm (Cause of death estimates) in the part of ‘Overview and data sources’ (lines 93-98).

2. - In the GBD 2021 methodology, 500 iterations were used to obtain the 95% uncertainty intervals (UI). Please correct this in the Methods section.

**Reply:** Thanks a lot for your professional comment, and we have corrected it in the part of ‘Statistical analysis’ (line 137).

3. - Please mention that LOESS regression was used to assess the association between SDI and prevalence rates, as presented in Figure 3.

**Reply:** Thanks a lot for your professional comment, and we have added the LOESS regression in the part of ‘Statistical analysis’ (lines 143-144).

4. - It is recommended to report the prevalence and DALY numbers for 2021 in the results of the abstract, in addition to the rates already provided.

**Reply:** Thanks a lot for your professional comment, and we have added the numbers of COPD in 2021 in the abstract.

We appreciate your warm work earnestly and hope that the revisions will meet the requirements. Once again, we are really grateful for the help from the editorial team and the reviewers. Hope everything is going well.

Yours sincerely，

Jing Xu.
